# N-Acylated Ciprofloxacin Derivatives: Synthesis and In Vitro Biological Evaluation as Antibacterial and Anticancer Agents

**DOI:** 10.1021/acsomega.3c00554

**Published:** 2023-05-18

**Authors:** Marta Struga, Piotr Roszkowski, Anna Bielenica, Dagmara Otto-Ślusarczyk, Karolina Stępień, Joanna Stefańska, Anna Zabost, Ewa Augustynowicz-Kopeć, Michał Koliński, Sebastian Kmiecik, Alina Myslovska, Małgorzata Wrzosek

**Affiliations:** † Chair and Department of Biochemistry, Medical University of Warsaw, ul. Banacha 1, 02-097 Warsaw, Poland; ‡ Faculty of Chemistry, University of Warsaw, Pasteura 1, 02-093 Warsaw, Poland; § Department of Pharmaceutical Microbiology, Centre for Preclinical Research, Medical University of Warsaw, 02-097 Warsaw, Poland; ∥ Department of Microbiology, National Tuberculosis and Lung Diseases Research Institute, 01-138 Warsaw, Poland; ⊥ Bioinformatics Laboratory, Mossakowski Medical Research Institute, Polish Academy of Sciences, 5 Pawinskiego Street, 02-106 Warsaw, Poland; # Biological and Chemical Research Centre, Faculty of Chemistry, University of Warsaw, 02-089 Warsaw, Poland; ¶ Department of Biochemistry and Pharmacogenomics, Faculty of Pharmacy, Medical University of Warsaw, 02-097 Warsaw, Poland

## Abstract

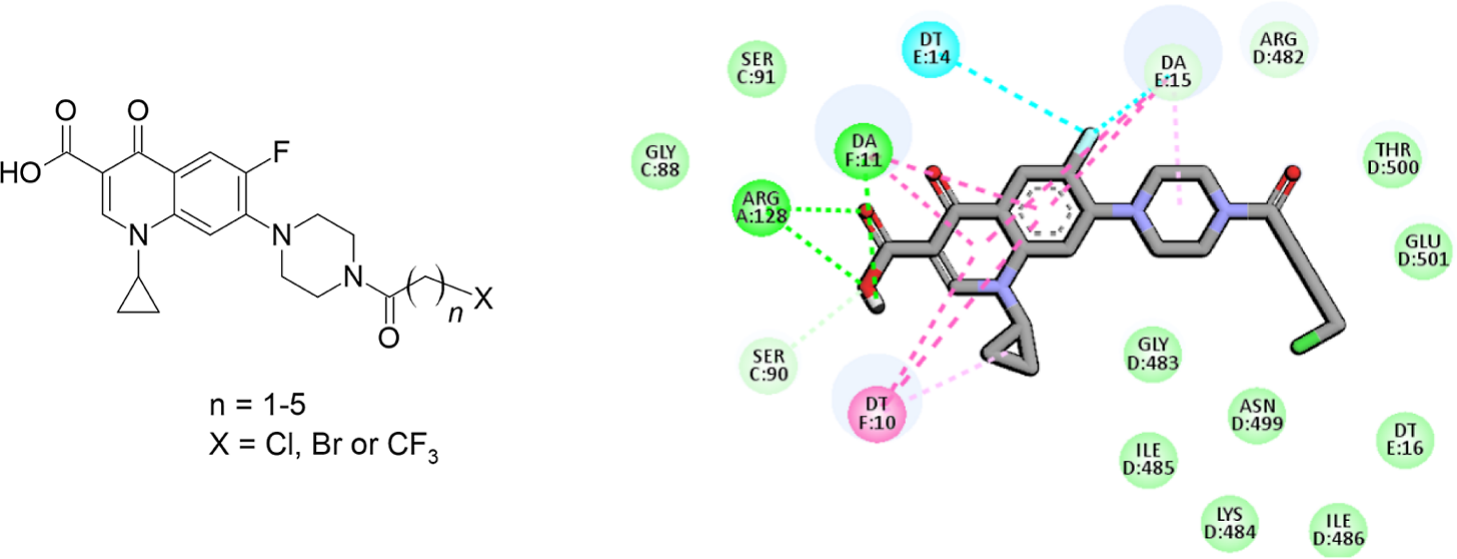

A novel series of N-acylated ciprofloxacin (CP) conjugates **1–21** were synthesized and screened as potential antimicrobial agents. Conjugates **1** and **2** were 1.25–10-fold more potent than CP toward all *Staphylococci* (minimal inhibitory concentration 0.05–0.4 μg/mL). Most of the chloro- (**3–7**), bromo- (**8–11**), and CF_3_-alkanoyl (**14–16**) derivatives expressed higher or comparable activity to CP against selected Gram-positive strains. A few CP analogues (**5**, **10**, and **11**) were also more effective toward the chosen clinical Gram-negative rods. Conjugates **5**, **10**, and **11** considerably influenced the phases of the bacterial growth cycle over 18 h. Additionally, compounds **2**, **4–7**, **9–12**, and **21** exerted stronger tuberculostatic action against three *Mycobacterium tuberculosis* isolates than the first-line antitubercular drugs. Amides **1**, **2**, **5**, **6**, **10**, and **11** targeted gyrase and topoisomerase IV at 2.7–10.0 μg/mL, which suggests a mechanism of antibacterial action related to CP. These findings were confirmed by molecular docking studies. In addition, compounds **3** and **15** showed high antiproliferative activities against prostate PC3 cells (IC_50_ 2.02–4.8 μM), up to 6.5–2.75 stronger than cisplatin. They almost completely reduced the growth and proliferation rates in these cells, without a cytotoxic action against normal HaCaT cell lines. Furthermore, derivatives **3** and **21** induced apoptosis/necrosis in PC3 cells, probably by increasing the intracellular ROS amount, as well as they diminished the IL-6 level in tumor cells.

## 1 Introduction

Bacterial inflammations, evoked mainly by Gram-positive and Gram-negative isolates, represent a serious health threat, being responsible for the majority of healthcare-associated infections that lead to health service overload and high mortality. The latest is caused by the growing fluoroquinolone resistance of pathogens to the current therapeutic options, involving methicillin-resistant or vancomycin-resistant *Staphylococci*, as well as *Streptococci*.^1^ The resistance develops mostly by amino acid alterations at various locations on subunit A of bacterial gyrase, located at the binding site of the enzyme, that diminishes the drug affinity to the gyrase–DNA complex.^1^ This mechanism of mutation was observed for *Streptococcus pneumoniae* isolates.^2,3^ The second reason of the drug resistance is the mutation in gene coding membrane proteins, responsible for the transport of a drug into the bacterial cell.^4^ That alteration reduces the level of transmembrane protein molecules or stimulates quinolone removal through efflux pumps. The above types of resistance were observed for some Gram-negative rods and *Staphylococcus aureus* strains,^1,3^ as well as for cancer cells.^5^

Fluoroquinolones, including ciprofloxacin (CP), moxifloxacin (MXF), and gatifloxacin, are a group of synthetic antibiotics that either inhibit the action of bacterial DNA gyrase, adenosine triphosphate-hydrolyzing topoisomerase II, and/or prevent from the detachment of gyrase from DNA.^6,7^ Topoisomerase II allows the relaxation of supercoiled DNA, by breaking, crossing over, and finally resealing both strands of the DNA chain.^8^

Quinolone chemotherapeutics are also able to inhibit bacterial topoisomerase IV, which participates in chromosomal DNA partition during cell division.^9,10^

The structural manipulations of the second-generation quinolones are a successful way of searching for new and more effective antibacterial drug candidates. A key advance in the development of CP-derived compounds is the enhanced activity against the isolates of *S. aureus*, *S. pneumoniae,* and group A streptococci.^11,12^ Several essential positions of the quinolone skeleton are subjects for modifications. Among them, fixed substituents at C-3 and C-6 are necessary for enhanced inhibition of DNA gyrase. The pharmacophore of CP necessary for powerful antimicrobial properties is the 4-pyridone nucleus with a carboxylic group attached to the C-3 position. Modification of this area, e.g., reduction to aldehyde or esterification, gave compounds with decreased antimicrobial potency, although an alteration of the 3-carboxylic functionality can be used for switching its activity to anticancer.^13^ On the other hand, when the carboxylic group is replaced with an isothiazole moiety, active antibacterial agents with weak solubility or improved toxicity were obtained.^14^ Interestingly, copper(II)–CP complexes, derived from hydroxamic acid, enhanced the permeability of *S. aureus* cells and affected proteins involved in virulence, the synthesis of nucleotides, and DNA repair mechanisms.^15^ The highly electronegative fluorine atom at C-6 seems to be optimal and crucial for the binding with bacterial DNA topoisomerase. Moreover, it was shown that the insubstantial aliphatic (e.g., ethyl) or alicyclic (cyclopropyl) functionality at the N-1 position of the quinolone is responsible for improved growth inhibitory properties.^16^ The same beneficial effect against Gram-positive isolates in comparison with CP was achieved by introduction of difluorophenyl or an oxetane ring, as well as the closure of both N-1 and C-8 atoms by an alkoxyamino moiety.^9,17^ Alkylation of the C-8 location favors in vivo parameters of the drug and raises its activity toward anaerobic rods.^18^ Addition of a methoxy group here, with concurrent alteration of the C-7 cyclic substituent, resulted in fourth-generation antibiotics, such as MXF. However, the newer generation of fluoroquinolones is not as potent as CP against *Pseudomonas aeruginosa* isolates.^12^ Similarly, the examples of third-generation quinolones (grepafloxacin and sparfloxacin) show that small substituents at C-5, such as methyl or amino functionalities, resulted in markedly increased activity.^17,19^ However, the most typical and reasonable strategy for the modification of the CP structure is the substitution of its C-7 position. The presence of an amino cyclic group at this location is the main feature responsible for improved action toward Gram-negative organisms and favorable pharmacokinetics.^20,21^ The chemical adjustments of unsubstituted piperazine in the CP skeleton included attachments of at least one methyl group (third-generation grepafloxacin and sparfloxacin), amino pyrrolidines (fourth-generation trovafloxacin), or a bicyclic ring system (MXF)^21^ but also N-acylation of the ring. Just among the amide analogues of CP, isatin,^22^ and sulfonamide,^23^ the C-7 derivatives exhibited excellent activity against standard and clinical Gram-positive and Gram-negative isolates. It was proved that the mentioned sulfonamide-based fluoroquinolones targeted both bacterial DNA gyrase and topoisomerase IV, exerting low or even no side effects on the central nervous system in mice.^23^ The modification of the piperazine nitrogen, in order to increase the lipophilicity and change the biological potency, was also described. The positive effects of the substitution of the piperazinyl ring by (hetero)aryl-triazole^24−27^ and alkylaryl^28^ fragments, its alkylation or acylation with alicyclic, alkyl, or halogenoalkyl moieties,^29−31^ on the overall antibacterial profile of CP were investigated. Moreover, conjugates of CP with cell-penetrating peptides were found to be inhibitors of the yeast type II DNA topoisomerase.^32^

Additionally, CP has been recommended as the second-line antitubercular agent, especially when resistance or intolerance to the first-line drugs appeared.^10,33^ However, when used as monotherapy or as the only effective tuberculostatic in a multidrug series, the risk of fluoroquinolone resistance rapidly develops.^34^ Recently, it was described that amide-tethered CP–isatin hybrids are severalfold more effective against multi-drug-resistant *Mycobacterium tuberculosis*(35) or standard H_37_Rv^22^ pathogens when compared with the parent drug. The modification of the piperazine moiety via a benzodiazepine moiety gave a new nontoxic agent, exerting antitubercular activity comparable to the quinolone antibiotic, with gyrase inhibition as a possible mechanism of action.^36^

What is more, the anticancer activities of fluoroquinolones are an effect of restraining of eukaryotic topoisomerase IIα, the analogue of DNA gyrase. It results in the formation of double-strand breaks in nucleic acids and incomplete DNA synthesis, which lead to S-phase arrest in a cancer cell.^37^ Since the affinity of CP to the eukaryotic enzyme is weaker than to the prokaryotic homologue, an anticancer treatment requires higher drug dosages in comparison with quantities used in bacterial infections. Therefore, the apoptosis-inducing dose of this quinolone, effective in antitumor therapies, equals at least 200–300 μg/mL.^38^ The anticancer properties of CP have been studied previously against various types of mammalian pathological cells, including bladder, colorectal, leukemia, and human prostate cancer cell lines. Cancerous cells exhibited different susceptibilities to this chemotherapeutic.^39−41^ Hepatocellular cancer cells (Hep G2) remained quite resistant, whereas colon carcinomas (SW-403) were found to be more sensitive.^39^ It has been estimated recently that the fluoroquinolone derivative induces oxidative stress in hepatoma-derived cells, increases activation of caspases involved in different pathways of cell death, and imposes double-strand breakages in the cellular DNA.^37^ Moreover, CP triggers apoptosis of human triple-negative breast cancer (MDA-MB-231) cells by leading to a loss of the mitochondrial transmembrane potential and stimulation of the cell cycle arrest at the S-phase.^6^ Interestingly, the effect of this antibiotic in prostate cancer (PC3) cells was mediated by the cell cycle arrest at the S-G2/M phase^41^ and by inhibition of NF-κB binding to DNA.^42^ It also gives a sensitizing effect and inhibits the efflux function of the ATP-binding cassette (ABC) transporter, decreasing the IC_50_ values of known chemotherapeutics in ABCB-1 overexpressing cells.^5^ Methylnaphthalenyl derivatives of CP induced the percentage of cells at G2/M phases, the percentage of cells in early apoptosis, and the level of the active caspase-3 in prostate cancer (PC-3) cells.^28^ Similar outcomes, correlated with topoisomerase I/II inhibition, were observed for renal cancer cell cultures treated with hydrazinyl hybrids^43^ and for leukemia cells incubated with urea-linked CP–chalcone compounds.^44^ An apoptosis-inducing effect and significant reduction in IL-6 levels were also observed after incubation of PC3 cells with CP fatty acid conjugates^45^ or its alkanoyl connections.^46^

We have initiated a screening program to search for new fluoroquinolone-derived compounds as potential antimicrobial agents with enhanced antitumor activity.^45,47^ In this study, the homogeneous series based on the CP pharmacophore was synthesized, by single modification on its C-7 position, and tested for their antibacterial, antitubercular, and cytotoxic properties, with determination of possible mechanisms of action.

## 2 Results and Discussion

### 2.1 Chemistry

**Scheme 1:**
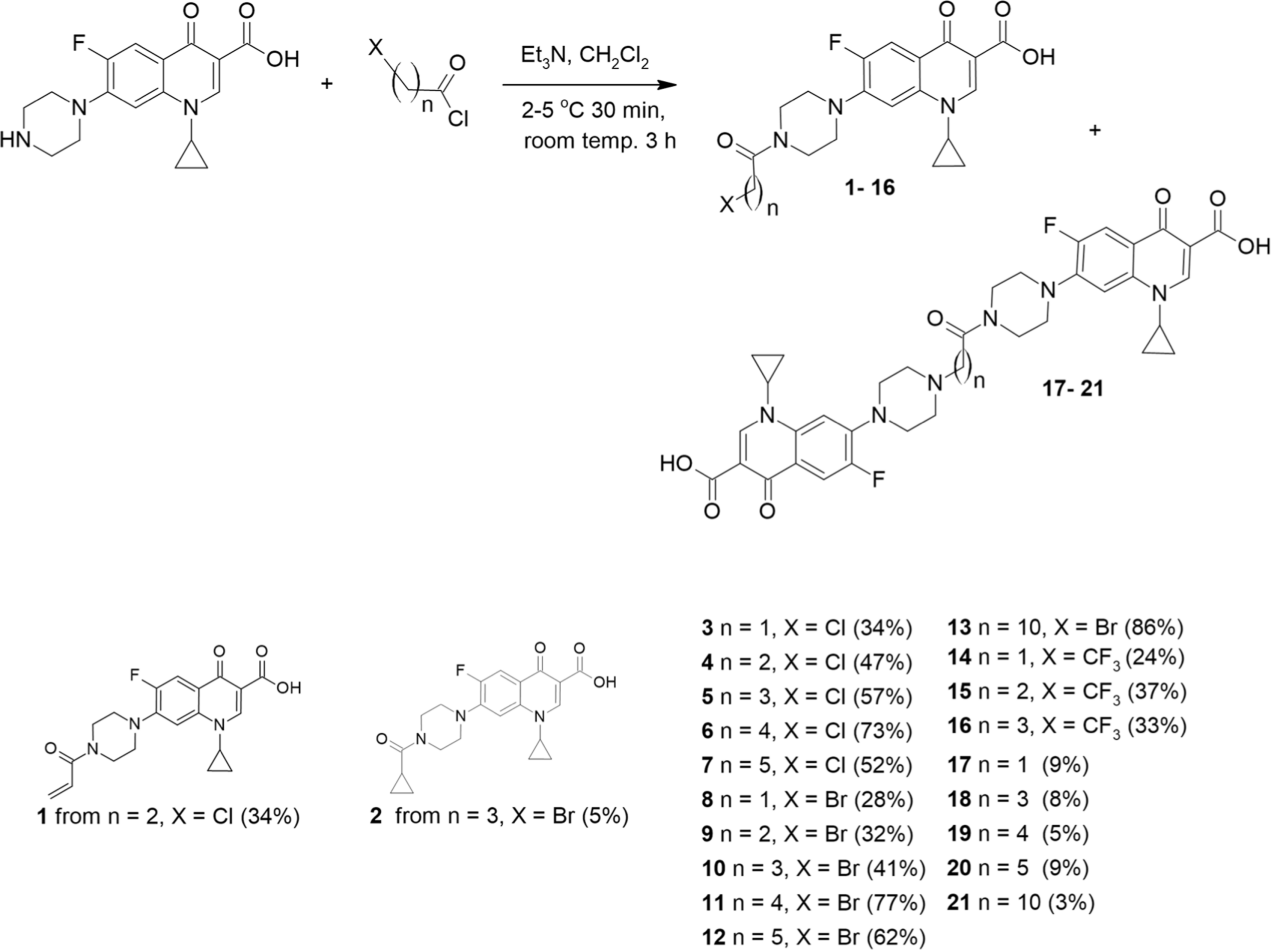
Synthesis of CP Amide Conjugates **1–21**

The aim of the research was to synthesize a versatile series of amide derivatives of CP by coupling its C-7 piperazinyl group with halogenated acyl chlorides, differing in hydrocarbon moiety and the type of halogen (Scheme 1). The conjugated acyl residues were unsaturated (**1**), alicyclic (**2**), or saturated (**3–21**). Aliphatic derivatives possessed either short (**3–12** and **14–20**) or medium (**13** and **21**) hydrocarbon chains. The carbon linker ended with chlorine (**3–7**), bromine (**8–13**), or trifluoromethyl (**14–16**) residue. The synthesis of presented compounds was conducted in a one-step reaction under mild conditions. After addition of acyl chloride to CP and triethyl amine suspension (at 2–5 °C) in dichloromethane, the resulting solution was stirred at room temperature for 3 h. The low yields of synthesis of short-chain amides **3** and **8** (28–34%) resulted from their poor solubility in the solvent and problems with separation and purification. Derivatives with a longer aliphatic chain (**4–7** and **10–13**) were obtained with higher yields (41–86%). The TLC analysis of reactions showed that side products were formed. After separation by column chromatography, the MS and NMR analyses confirmed that double-condensation products **17–21** were synthesized, with yields below 10%. On the other hand, the reaction of CP with 3-chloropropionyl chloride yielded the unsaturated derivative **1**, instead of the dimeric product. Amide **2** was formed with a yield of 5% as a product of degradation of compound **10** during the purification process by column chromatography.

### 2.2 Biological Studies

#### 2.2.1 In Vitro Antibacterial Activity

The antibacterial properties of the synthesized fluoroquinolone conjugates **1–21** were investigated by employing standard Gram-positive bacteria (*S. aureus* NCTC 4163, ATCC 25923, ATCC 6538, and ATCC 29213 and *Staphylococcus epidermidis* ATCC 12228 and ATCC 35984) and Gram-negative rods (*P. aeruginosa* ATCC 15442 and *Escherichia coli* ATCC 25922). As presented in Table 1, derivatives **1** and **2** showed great antibacterial properties, being 1.25–10-fold more active than the reference CP toward all *Staphylococci* [minimal inhibitory concentration (MIC) 0.05–0.4 μg/mL]. The acryloyl compound **1** exerted a 5–10 times higher potency than the fluoroquinolone antibiotic against two *S. aureus* pathogens (NCTC 4163 and ATCC 25923). In addition, majority of the halogenoalkyl derivatives (**3–11** and **14–16**) were equally or more potent than CP toward the studied Gram-positive cocci. For molecules with a linker of up to five carbons, no clear dependence between the properties of chloro- and bromoalkanoyl analogues was found; all of them were comparably highly active. Achieving MICs of 0.1–0.4 μg/mL, they were 1.25–2.5 times more effective than unbound CP against most of the Staphylococcal pathogens.

**Table 1:**
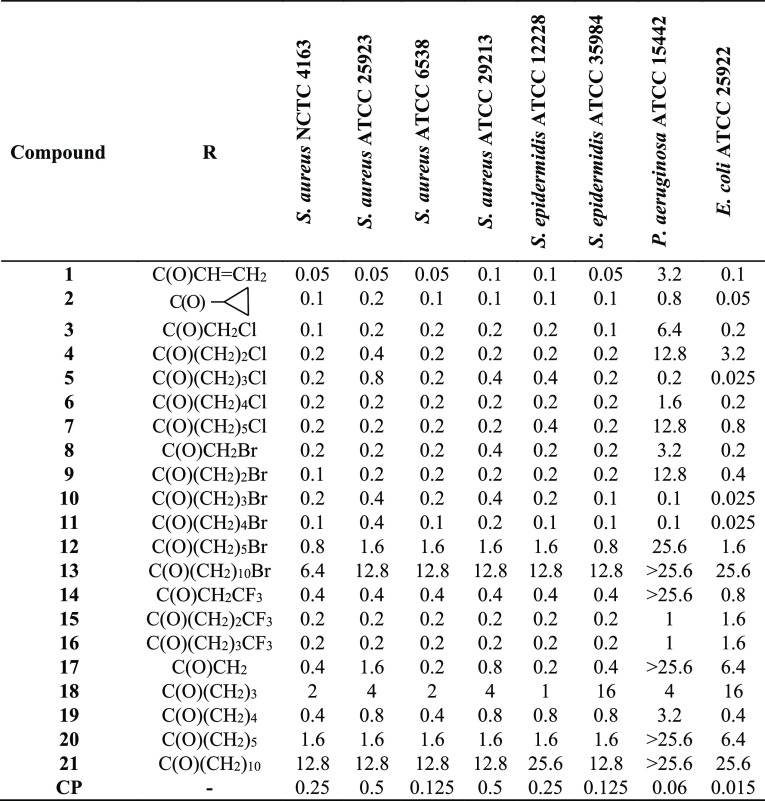
Activity of Compounds **1–21** against Standard Bacterial Strains— MICs (μg/mL)

The antimicrobial potential of substituted chlorohydrocarbon moieties toward Gram-positive rods is decreasing as follows: acetyl > pentanoyl > propanoyl > hexanoyl ≫ butanoyl, however without sharp differences in their overall biological strength. The chloroacetyl conjugate (**3**) was the most powerful; however, the growth inhibitory properties of longer alkanoyls (**4**, **6**, and **7**) were similarly strong against all strains (MIC 0.1–0.8 μg/mL). The chlorobutanoyl derivative (**5**) seemed to be a stronger inhibitor than CP, but only toward two selected pathogens. Within the bromine-containing analogues, the pentanoyl linker (**11**) was the limiting value of the strong antimicrobial activity. That compound was 1.25–2.5 more active than CP against all tested strains (MIC 0.1–04 μg/mL). Other bromoalkanoyls (**8–10**) assigned the same level of biological potency as the reference drug toward four *Staphylococci*. In contrast to the chlorohexanoyl (**7**) derivative, more effective toward half of the Gram-positive rods (MIC 0.2–0.4 μg/mL) as compared to CP, the bromohexanoyl analogue (**12**) possessed moderate activities (MIC 0.8–1.6 μg/mL). Thus, the group of compounds with bromoalkanoyl linkers can be ranked as follows (in decreasing order): pentanoyl > butanoyl > propanoyl > acetyl ≫ hexanoyl > undecanoyl. The 3-trifluoromethylalkanoyl series (**14–16**) was a little less potent than the above-mentioned monohalogenoalkanoyl counterparts. Whereas both butanoyl (**15**) and pentanoyl (**16**) derivatives were equally active (MIC 0.2 μg/mL) toward all Gram-positive strains, the propanoyl compound (**14**) assigned half of the activity of its close analogues, being still more potent against two *Staphylococci*, as compared to CP. The dimeric compounds (**17–21**) constituted a group with the weakest antimicrobial properties, and the longer the spacer, the greater decrease in antimicrobial properties was observed. The most potent acetyl derivative **17**, with MIC equaled 0.2–1.6 μg/mL against four strains, was less active than the reference drug. Considering the susceptibility of standard Gram-negative pathogens to the studied conjugates, two bromo derivatives, butanoyl (**10**) and pentanoyl (**11**), at a MIC of 0.025–0.1 μg/mL maintained 60% of CP potency against both studied standard rods. Less powerful chlorobutanoyl (**5**) and cyclopropanecarbonyl (**2**) analogues kept 30–60% of the drug efficiency (MIC 0.025–0.2 μg/mL). Furthermore, other chloro- (**1**, **3**, **6**, and **7**), bromo- (**8** and **9**), trifluoromethylalkanoyl (**14–16**), and dimeric (**19**) derivatives, applied at a MIC of ≤ 1 μg/mL, exhibited considerable antimicrobial properties, mainly against the more susceptible strain of *E. coli*.

The activity of all derivatives was next tested against an expanded panel of clinical isolates (Table 2). The MICs of the most potent amides toward Staphylococcal pathogens were in the range from 0.1 to 0.5 μg/mL. Studied *S. aureus* strains revealed to be more sensitive as compared to *S. epidermidis*. The strongest growth inhibitory potency toward *S. aureus* 180 and 5595 isolates was observed for the chloropentanoyl (**6**) derivative, which at 0.1 μg/mL acted 5-fold more effective than the reference. Similarly, its chloropropanoyl analogue (**4**) was 2.5 times more active than CP. Amides bearing cyclopropanecarbonyl (**2**), chloroacetyl (**3**), and chlorobutanoyl (**5**) fragments inhibited bacterial growth at 0.4 μg/mL, stronger than the reference antibiotic. The same level of activity against the *S. aureus* 5595 isolate was observed for acetyl conjugates **8** and **17**. The hexanoyl (**7** and **12**), propanoyl (**9** and **14**), and pentanoyl (**15**) derivatives were as potent as CP against two mentioned Staphylococcal strains (180 and 5595). Moreover, compounds **1**, **10**, **11**, and **19** gained 62.5% of CP inhibitory activity. Concerning other *S. aureus* isolates (T5595 and T5591), amides **2**, **3**, **6**, **9**, and **14** acted effectively at 0.1–0.2 μg/mL, being 1.25–2.5 times more powerful as CP. Compounds **4**, **5**, **7**, **15**, and **16** with MICs of 0.4 μg/mL had 62.5% of the reference activity. Among *S. epidermidis* rods, the KR4243/1 strain was the most susceptible to the presence of the studied amides. Conjugates **2–7**, **9**, **14**, **15**, and **17** inhibited its growth at a concentration of 0.1–0.2 μg/mL, which gave 1.25–2.5 of the strength of CP alone. Similarly, it was observed that derivatives **2–6** and **8** exerted at 6.4–12.8 μg/mL a stronger activity than the standard drug toward the more resistant *S. epidermidis* 4341 strain. Several amides (**2**, **4**, **6**, **8**, and **17**) were as potent as the reference against the *S. epidermidis* 5253 isolate (MICs 12.8–25.6 μg/mL). Additionally, conjugates **2–11**, **14**, and **15** expressed 65% of CP activity against the *Staphylococcus pasteuri* strain. Gram-negative isolates of clinical origin appeared to be considerably less vulnerable to the presence of synthesized quinolone derivatives. The highest activity was observed for the 3-chlorobutanoyl compound (**5**), followed by bromoalkanoyl substances with four-carbon (**10**) and five-carbon (**11**) atoms in their amide fragment. The growth of two *E. coli* rods was inhibited by derivative **5** at 0.025 μg/mL, which was 2.4–1.2 times lower than the inhibitory concentration observed for CP. Moreover, the mentioned compounds applied at 0.05 and 0.8 μg/mL were 1.2 times more potent than the standard antibiotic against *Escherichia cloacae* and *P. aeruginosa* 37 strains, respectively. The same doses of these compounds were effective toward other selected *E. coli* and *P. aeruginosa* pathogens, gaining at least 60% of the reference drug activity. Among other tested substances, the cyclopropanecarbonyl conjugate **2** was highly potent against the *E. cloacae* isolate, at MIC 1.2 times lower than CP alone. Only amides **5**, **10**, and **11** at 6.4 μg/mL exhibited the growth inhibitory potency against *Klebsiella pneumoniae* rods, achieving 62.5% of the reference activity. Moreover, compounds **2–4**, **6**, **8**, **9**, **12**, **14**, and **18** acted against selected *E. coli* and *E. cloacae* strains at low concentrations of 0.1–0.8 μg/mL.

**Table 2:** Activity of Compounds **1–21** against Clinical Bacterial Strains— MICs (μg/mL)

compound	S. aureus 180	S. aureus 5595	S. aureus T5595	S. aureus T5591	S. epidermidis 4341	S. epidermidis 5253	S. epidermidis KR4243/1	S. pasteuri KR 4358	P. aeruginosa 37	P. aeruginosa 659	E. coli 951	E. coli 520	E. coli 600	E. cloacae 8	K. pneumoniae 28	K. pneumoniae 510
1	0.8	0.8	0.4	0.8	>25.6	>25.6	0.4	0.4	>25.6	>25.6	>25.6	1.6	>25.6	3.2	>25.6	>25.6
2	0.4	0.4	0.2	0.2	12.8	25.6	0.2	0.2	12.8	6.4	6.4	0.2	0.2	0.05	>25.6	25.6
3	0.4	0.4	0.2	0.1	12.8	>25.6	0.2	0.2	>25.6	>25.6	>25.6	0.4	0.4	0.8	>25.6	25.6
4	0.2	0.2	0.4	0.4	6.4	25.6	0.2	0.2	>25.6	>25.6	25.6	0.8	0.8	0.8	>25.6	>25.6
5	0.4	0.4	0.4	0.4	12.8	>25.6	0.2	0.2	0.8	0.8	0.8	0.025	0.025	0.05	>25.6	6.4
6	0.1	0.1	0.2	0.2	6.4	12.8	0.2	0.2	6.4	12.8	12.8	0.4	0.4	0.8	>25.6	>25.6
7	0.5	0.5	0.4	0.4	25.6	>25.6	0.2	0.2	>25.6	>25.6	>25.6	6.4	6.4	16	>25.6	>25.6
8	0.8	0.4	0.8	1.6	12.8	25.6	0.4	0.2	12.8	6.4	6.4	0.2	0.2	0.8	>25.6	25.6
9	0.5	0.5	0.2	0.1	16	128	0.1	0.2	128	>25.6	64	0.8	–0.4	2	>25.6	>25.6
10	0.8	0.8	0.8	0.8	25.6	>25.6	0.4	0.2	0.8	0.8	0.4	0.1	0.05	0.05	>25.6	6.4
11	0.8	0.8	0.8	0.8	25.6	>25.6	0.4	0.2	0.8	0.8	0.4	0.1	0.05	0.05	>25.6	6.4
12	0.5	0.5	0.8	0.8	64	>25.6	0.8	0.8	>25.6	>25.6	>25.6	0.8	0.4	>25.6	>25.6	>25.6
13	>25.6	>25.6	>25.6	>25.6	>25.6	>25.6	>25.6	>25.6	>25.6	>25.6	>25.6	>25.6	>25.6	>25.6	>25.6	>25.6
14	0.5	0.5	0.2	0.2	>25.6	>25.6	0.1	0.2	>25.6	>25.6	>25.6	0.4	0.8	2	>25.6	>25.6
15	0.5	0.5	0.4	0.4	>25.6	>25.6	0.2	0.2	>25.6	>25.6	>25.6	3.2	3.2	>25.6	>25.6	>25.6
16	1	1	0.4	0.4	25.6	>25.6	0.4	0.4	>25.6	>25.6	>25.6	3.2	6.4	8	>25.6	>25.6
17	1.6	0.4	0.8	1.6	25.6	25.6	0.2	1.6	>25.6	>25.6	>25.6	3.2	12.8	25.6	>25.6	>25.6
18	6.4	6.4	6.4	6.4	>25.6	>25.6	3.2	3.2	12.8	>25.6	6.4	1.6	0.4	0.8	>25.6	>25.6
19	1.6	0.8	1.6	0.8	>25.6	>25.6	1.6	1.6	>25.6	25.6	25.6	3.2	3.2	3.2	>25.6	>25.6
20	1	1	0.8	0.8	>25.6	>25.6	0.8	0.8	>25.6	>25.6	>25.6	12.8	12.8	>25.6	>25.6	>25.6
21	>25.6	>25.6	>25.6	>25.6	>25.6	>25.6	>25.6	>25.6	>25.6	>25.6	>25.6	>25.6	>25.6	>25.6	>25.6	>25.6
CP	0.5	0.5	0.25	0.25	16	>25.6	0.25	0.13	1	0.5	0.5	0.06	0.03	0.06	>25.6	4

To sum up, all chloroalkanoyl derivatives were highly active against Gram-positive hospital isolates, independently of the type of bacterial strain. Their growth inhibitory strength slightly and gradually got lower from that of chloropentanoyl (**6**), cyclopropanecarbonyl (**2**), acetyl (**3**), propanoyl (**4**), butanoyl (**5**), and hexanoyl derivative (**7**). Within the group of CF_3_-containing compounds, the longer was the alkanoyl linker, the weaker activity was assigned. Only CF_3_-propanoyl amide (**14**) was as potent as the most active monohalogen conjugates (**3** and **9**). A similar tendency was noticed for bromoalkanoyl derivatives: as the hydrocarbon chain got longer, the biological properties have decreased. Bromopropanoyl (**9**), acetyl (**8**), but also inexpertly hexanoyl (**12**) amides were the most effective in this group. Among dimeric derivatives (**17–21**), the most active was that with the shortest linker (**17**), however severalfold weaker in comparison with CP-derived monomers. As compared with the susceptibility of standard strains, the group of compounds active toward hospital isolates was restricted. Spontaneous mutation and horizontal gene transfer observed for clinically isolated bacteria mostly reduced their sensitivity to antimicrobial agents. Derivatives highly effective against both collections of Gram-positive bacteria included mainly the cyclic analogue (**2**), chloroalkanoyls (**3–6**), and shorter CF_3_-anlanoyl conjugates (**14** and **15**). The same compounds (**5**, **10**, and **11**) were active against Gram-negative isolates of different origin.

#### 2.2.2 Bacterial Growth Curve Assay

**Figure 1:**
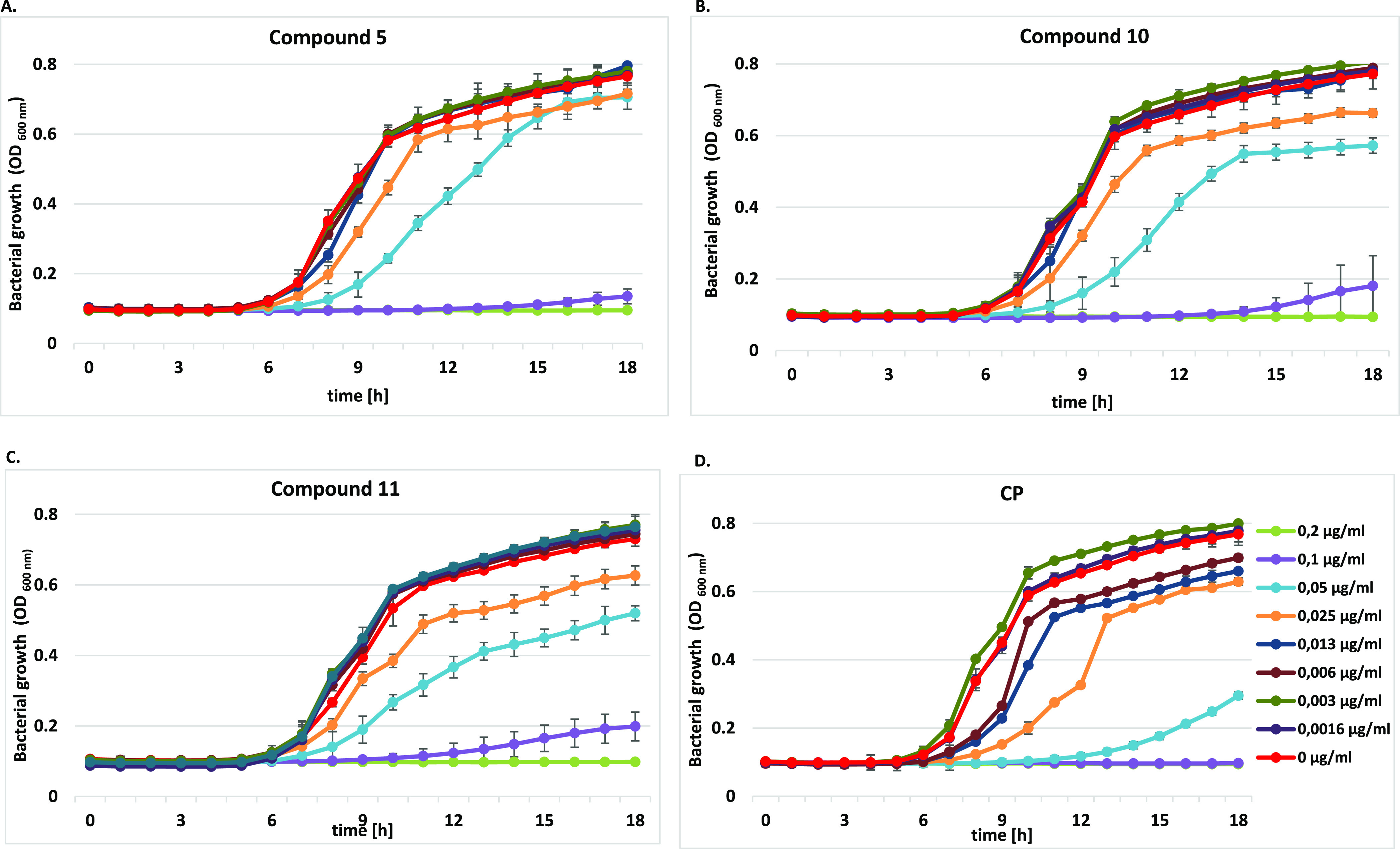
Growth curve analysis of *P. aeruginosa* ATCC 15442 at an absorbance of 600 nm (OD_600_) with or without different concentrations of (A) compound **5**, (B) compound **10**, (C) compound **11**, and (D) CP for 18 h. The growth curve data were plotted as average values with standard deviations of *n* = 3.

**Figure 2:**
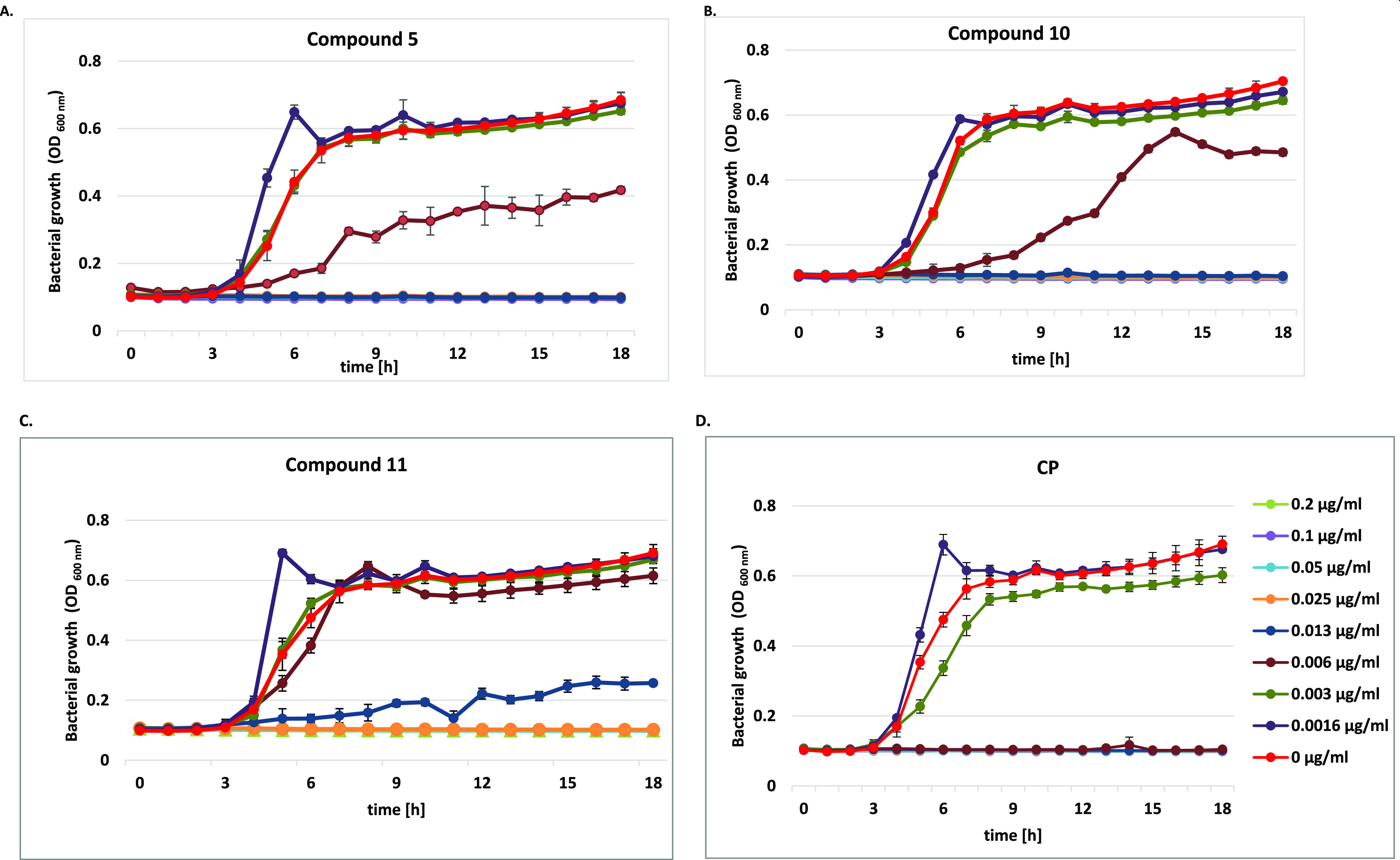
Growth curve analysis of *E. coli* ATCC 25922 at an absorbance of 600 nm (OD_600_) with or without different concentrations of (A) compound **5**, (B) compound **10**, (C) compound **11**, and (D) CP for 18 h. The growth curve data were plotted as average values with standard deviations of *n* = 3.

The growth curves of *P. aeruginosa* ATCC 15442, *E. coli* ATCC 25922, and *S. aureus* ATCC 6538 strains, representing changes in the number of bacterial populations over 18 h in culture, were generated (Figures 1, 2, and S1). Conjugates **5**, **10**, and **11** in the concentration range between 0.8 and 0.0016 μg/mL were selected for these tests, as they exerted both the strongest potency toward Gram-negative isolates and low MIC values against *Staphylococci*. The obtained results supplemented MICs presented previously in Table 1, as well as illustrated the impact of compounds on the three distinct phases of the bacterial growth cycle, lag, exponential (log), and stationary, compared with controls and CP alone.

As shown in Figure 1, the clearest concentration—activity dependence was found for *P. aeruginosa* isolates. All tested compounds visibly influenced the phases of bacterial cell growth (Figure 1A–C). CP conjugates at doses ≥0.2 μg/mL fully inhibited bacterial growth. Their concentration of 0.1 μg/mL caused an extension of the initial (lag) phase; thus, the cells did not enter the exponential phase. It was also noticed that the studied amides dosed at 0.03 and 0.05 μg/mL still limited bacterial cell metabolism. In the presence of derivative **5**, the exponential phase was shifted to 7–12 and 8–15 h, respectively, of the experiment, as compared with the control (Figure 1A). The reference chemotherapeutic CP, applied at ≥0.1 μg/mL, completely inhibited *P. aeruginosa* cell growth (Figure 1D). At doses of 0.03–0.006 μg/mL, it influenced cell metabolism but did not suppress cell divisions. An elongation of the exponential phase to 9 h was noticed then. When used at 0.05 μg/mL, it elongated the lag phase up to 14 h.

Moreover, the studied derivatives influenced *E. coli* strains in a different way (Figure 2). Unbound CP inhibited bacterial cell growth at ≥0.006 μg/mL, while derivatives **5** and **10** applied at ≥0.013 μg/mL. The inhibitory concentration of compound **11** was the highest (≥0.025 μg/mL). A sharp increase in the absorbance within 6 h of the experiment, following by an acute decrease after 7 h, was observed in the presence of CP alone or their conjugates **10** and **11**, used at the lowest doses of 0.0016 μg/mL. It corresponded with a reduction of the live bacteria population. CP at 0.003 μg/mL disturbed cell metabolism, as the absorbance values of the experimental phase were lower in comparison with the control. The lag phases in the presence of conjugates **5** and **10** applied at 0.006 μg/mL were elongated to 6–9 h, respectively. In contrast to the control, the growth rate of the bacteria co-cultured with **10** was extended between 9 and 14 h of the test, whereas the short exponential phase in the presence of compound **5** passed into the prolonged stationary phase. Compound **11** strongly altered cell metabolism already at a concentration of 0.013 μg/mL, as the bacterial cells did not reach its exponential phase.

The impact of CP conjugates on the growth of *S. aureus* ATCC 6538 isolate was also remarkable (Figure S1), which correlated with the observed MIC values. All tested fluoroquinolones fully suppressed the cell growth at ≥0.2 μg/mL. CP, when applied at 0.1 μg/mL, limited the bacterial growth up to 12 h of the experiment, after which the cells doubled the number. The same concentrations of all CP derivatives shifted the doubling time and elongated the exponential phases to 8–9 h, as compared to controls. What is more, for compound doses ranging between 0.05 and 0.006 μg/mL, the maximal growth rate of bacteria reached lower values than for control experiments.

#### 2.2.3 Biofilm Eradication Activity

**Figure 3:**
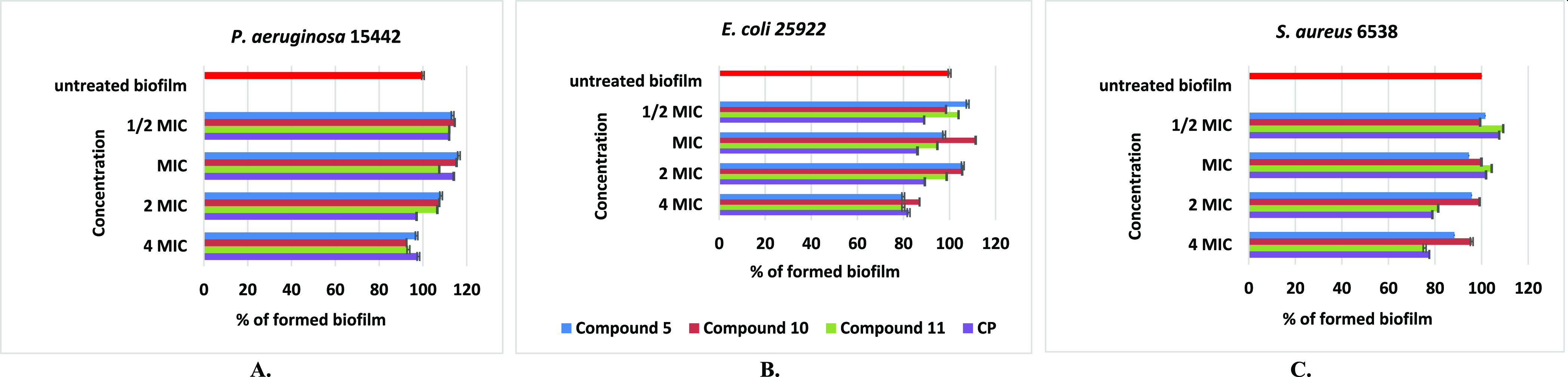
Effect of different doses of compounds **5**, **10**, **11**, and CP on the biofilm formed by *P. aeruginosa* ATCC 15442 (A), *E. coli* ATCC 25922 (B), and *S. aureus* 6538 (C) strains.

Preliminary antibacterial studies revealed that derivatives **5**, **10**, and **11** are the most potent against free-floating planktonic cells of standard Gram-negative strains of *P. aeruginosa* ATCC 15442 and *E. coli* ATCC 25922 and strongly active toward *S. aureus* isolates; thus, they were further studied for their ability to eradicate the mature biofilm of the same pathogens. They were tested at doses ranging from 1/2 MIC to 8 MIC (according to Table S1), compared to the untreated biofilm biomass as the positive control. None of the tested compounds, even CP, have achieved minimum biofilm eradication concentration (MBEC), which eradicates ≥50% of the biofilm structure (Figure 3). A moderate activity was observed for thiourea derivatives **5** and **11** and unbound CP, which at a concentration of 4 MIC eradicated the biofilm formed by the *E. coli* strain by approx. 20%. Moreover, at doses of 4–2 MIC, conjugate **11** and CP alone eliminated the mature biofilm of *S. aureus* ATCC 6538 isolates by 19–25%.

#### 2.2.4 In Vitro Antimycobacterial Activity

Considering the importance of CP and its analogues in the second-line antitubercular therapy, a total of 21 new CP-based conjugates were screened for in vitro activity against *M. tuberculosis* H_37_Rv, Spec. 192, and Spec. 210 strains. The results are given in Table 3. Most of the synthesized derivatives were found to be active at the low micromolar range (0.25–2 μg/mL). A set of cyclic (**2**) and aliphatic (**5–7** and **10–12**) amides were effective at ≤0.25 μg/mL toward the most sensitive *M. tuberculosis* Spec. 210, being equipotent to CP and 64–128 more effective than all reference tuberculostatics. Other compounds (**1**, **3**, **4**, **9**, **13**, **19**, and **21**), with MICs ranging from 1 to 2 μg/mL, displayed 8–32-fold stronger growth inhibitory properties than the antimycobacterial drugs used. Also, conjugates **8** and **18** were 2–8-fold more potent as compared to standards.

**Table 3:** Activity of the Synthesized Compounds against Selected *Mycobacterium tuberculosis* Strains—MICs (μg/mL)

compound	M. tuberculosis H_37_Rv	M. tuberculosis Spec. 192 (sensitive to tuberculostatics)	M. tuberculosis Spec 210 (multidrug-resistant)
1	4	4	2
2	1	0.5	≤0.25
3	4	2	2
4	2	1	2
5	0.5	0.5	≤0.25
6	0.5	0.5	≤0.25
7	0.5	0.5	≤0.25
8	32	32	8
9	2	2	1
10	1	0.5	≤0.25
11	0.5	0.5	≤0.25
12	1	1	≤0.25
13	2	4	1
14	64	64	32
15	64	64	32
16	64	64	32
17	32	32	16
18	8	16	4
19	8	8	1
20	32	16	16
21	2	2	1
CP	0.5	0.5	0.25
isoniazid (INH)	0.125	0.125	16
rifampicin (RMP)	1	1	32
streptomycin (SM)	1	1	16
ethambutol(EMB)	2	2	32

Similarly, the most promising derivatives mentioned above (**2**, **5–7**, **10**, and **11**) exerted high activity (at 0.5 μg/mL) against *M. tuberculosis* Spec. 192, being 2–4-fold more potent in comparison with RMP, SM, or EMB. Most of these compounds (**5–7** and **11**) were also equipotent to Spec. H_37_Rv. The growth inhibitory properties against this isolate of other derivatives, applied at a concentration of 1–2 g/mL, were as good as those of both RMP and SM (compounds **2**, **10**, and **12**) or EMB (**4**, **9**, **13**, and **21**). Additionally, derivatives **3**, **4**, **9**, **12**, and **21** expressed biological effects toward Spec. 192 comparable to the used tuberculostatics. Unlike others, amides **14–16** bearing a 3-CF_3_-alkanoyl moiety were poorly potent against studied mycobacteria (MICs 32–64 μg/mL).

To sum up, the longer was the alkanoyl chain, the higher antimycobacterial activity was observed. Short-chain chloroalkanoyls represented the most potent group and were followed by bromoalkanoyl derivatives, then moderately effective dimers, and finally weakly active CF_3_-derived amides. Chlorine-containing compounds with four to six carbon linkers (**5–7**), as well as the bromopentanoyl derivative (**11**), were comparably active, more than also similarly potent cyclopropanecarbonyl (**2**) and bromobutanoyl (**10**) conjugates. Equally good antituberculostatic activities were found for bromohexanoyl (**12**), chloropropanoyl (**4**), undecanoyl (**21**), and bromopropanoyl (**9**) compounds. In contrast, the antitubercular action of chloroalkanoyls of the shortest chains (**1** and **3**) was only moderate.

#### 2.2.5 Inhibition of Bacterial DNA Topoisomerases

The antibacterial properties of fluoroquinolones are the result of the inhibition of type II topoisomerases—first of the subunits gyrA and gyrB of DNA gyrase, which is responsible for replication, recombination, and repair of DNA. The second target is topoisomerase IV, accounted for the decatenation of the linked daughter chromosomes at the terminal stages of DNA replication. Quinolone drugs bind to the complex formed between DNA and the topoisomerase enzyme, forming a three-component complex, which inhibits bacterial cell growth.^23,24^

**Figure 4:**
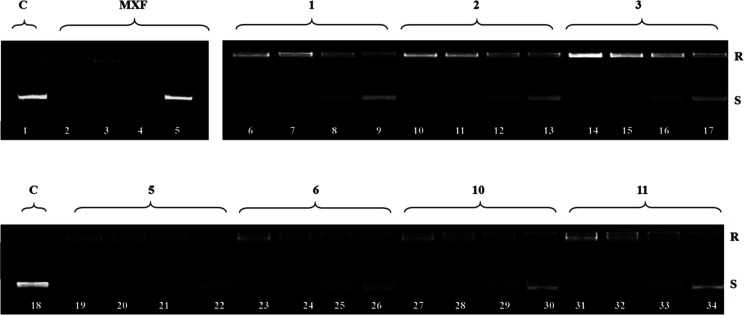
Electrophoretic analysis of *S. aureus* DNA gyrase supercoiling assay showing relaxed (R) and supercoiled DNA (S) bands at concentrations of 64, 32, 8, and 2 μg/mL of tested conjugates **1–3**, **5**, **6**, **10**, and **11** and the effect of tested compounds on gyrase activity.

**Figure 5:**
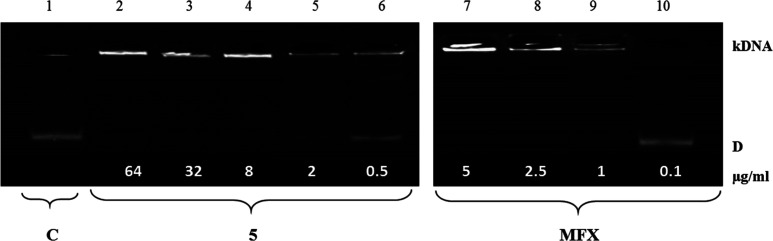
Electrophoretic analysis of *S. aureus* topoisomerase IV decatenation assay showing catenated kDNA and the decatenated mini circles (D) and the effect of tested compounds on topoisomerase IV activity. Decreasing amounts of compounds were incubated with 200 ng of kinetoplast DNA and run on agarose gel. Lane 1: *S. aureus* topoisomerase IV assay with dilution buffer (control, C). Lanes 2–6: compound **5** at concentrations 64, 32, 8, 2, and 0.5 μg/mL, respectively. Lanes 7–10: MFX at concentrations 5, 2.5, 1, and 0.1 μg/mL, respectively.

To compare the enzyme inhibitory properties of the most active antimicrobial agents (**1–3**, **5**, **6**, **10**, and **11**) with parental quinolones, their impact on *S. aureus* DNA gyrase and topoisomerase IV was evaluated (Table 4 and Figures 4 and 5). The amide **5** inhibited DNA supercoiling by gyrase and topo IV, with IC_50_ values of the same order of activity as the references (3.2 and 2.7 μg/mL, respectively), which suggests a related mechanism of action. Compounds **1**, **2**, **6**, **10**, and **11** showed almost no difference in activities (IC_50_ ≤ 10 μg/mL), and the results obtained for them indicate that the synthesized CP-derived amides may act similarly by suppression of bacterial DNA topoisomerases.

**Table 4:** IC_50_ Values for the Inhibition of Catalytic Activities of *S. aureus* Topoisomerases^a^

	IC_50_ (μg/mL)	
compound	S. aureus DNA gyrase	S. aureus topoisomerase IV
**1**	9.2 ± 0.3	10.0 ± 1.2
**2**	8.9 ± 0.2	9.5 ± 0.5
**3**	25 ± 0.5	32 ± 1.5
**5**	3.2 ± 0.4	2.7 ± 0.2
**6**	5.4 ± 0.6	8.0 ± 1.2
**10**	7.9 ± 0.5	5.0 ± 0.4
**11**	8.0 ± 0.5	6.0 ± 0.3
**CP**	3.0 ± 0.1	1.9 ± 0.1
**MXF**	2.0 ± 0.5	1.5 ± 0.2

a IC_50_; concentration of the compound that inhibits the enzyme activity by 50%.

Decreasing amounts of compounds were incubated with 500 ng of relaxed pBR322 and run on agarose gel. Lanes 1 and 18: *S. aureus* DNA gyrase with solvent (control). Lanes 2–5: MFX at concentrations 5, 2.5, 1, and 0.1 μg/mL, respectively. Lanes 6–9: compound **1**, lanes 10–13: compound **2**, lanes 14–17: compound **3**, lanes 19–22: compound **5**, lanes 23–26: compound **6**, lanes 27–30: compound **10**, and lanes 31–24: compound **11**.

#### 2.2.6 Molecular Docking Studies

**Figure 6:**
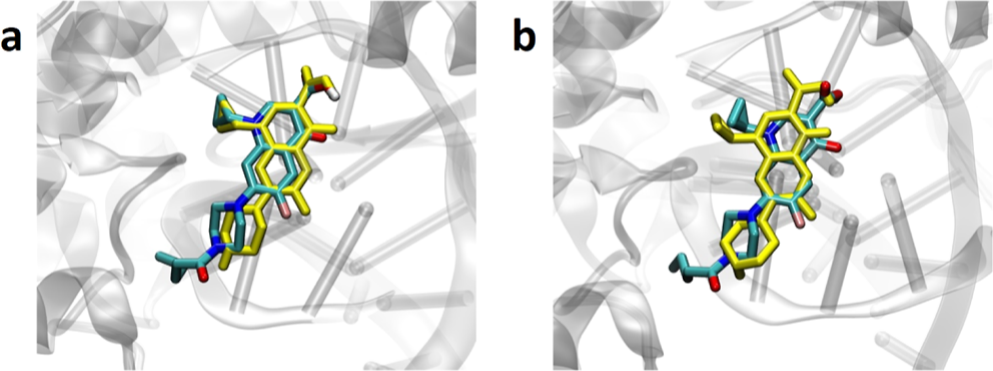
Binding modes of compound **5** docked to (a) DNA gyrase structure (PDB ID: 5BTC)^48^ and (b) DNA topoisomerase IV structure (PDB ID: 3RAD).^49^ For comparison, the structure of docked **CP** is shown in yellow.

**Figure 7:**
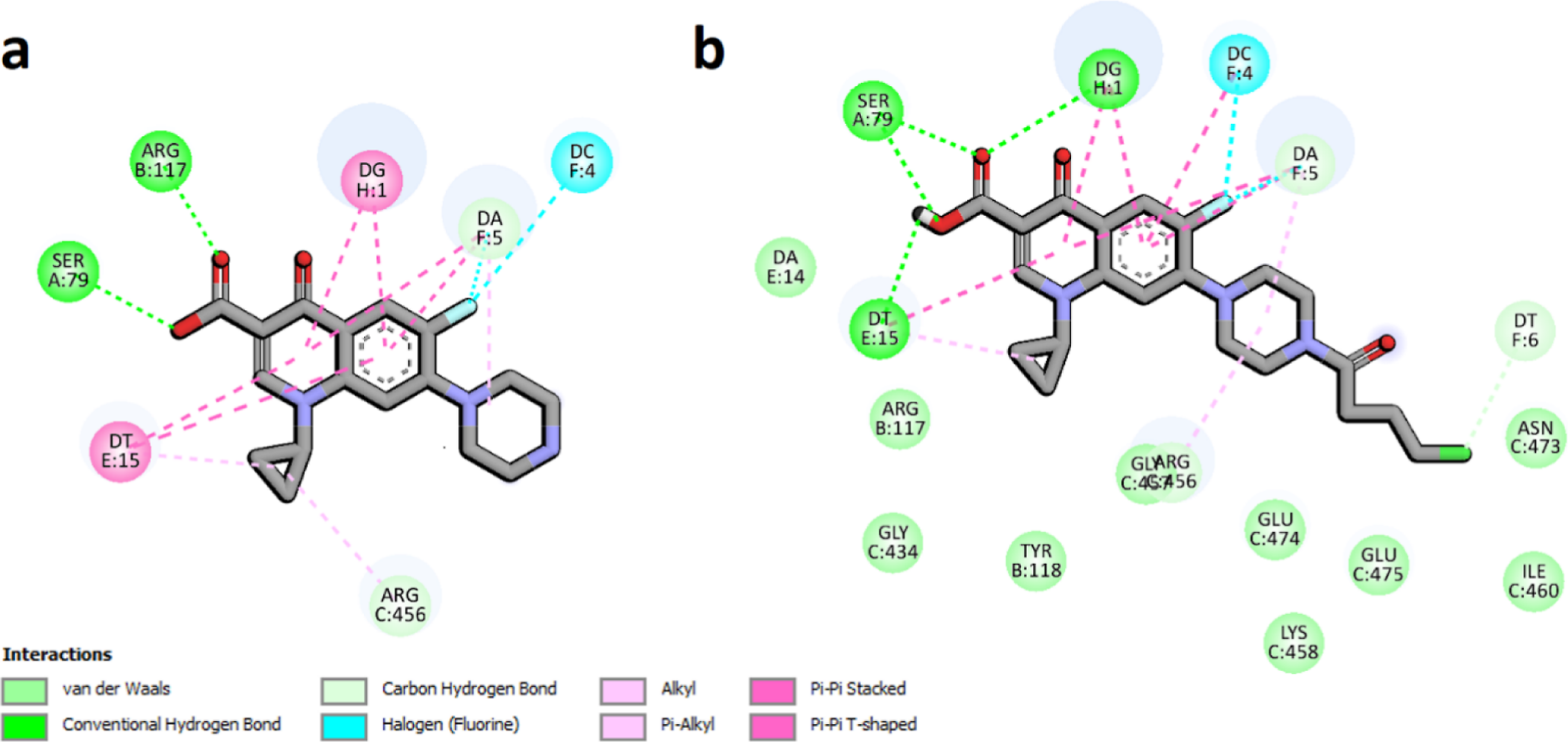
Interaction schemes generated for DNA gyrase and bound CP—panel (a) and compound **5**—panel (b). Colors presenting different interaction types are shown at the bottom. Figure generated using the BIOVIA Discovery Studio.

**Figure 8:**
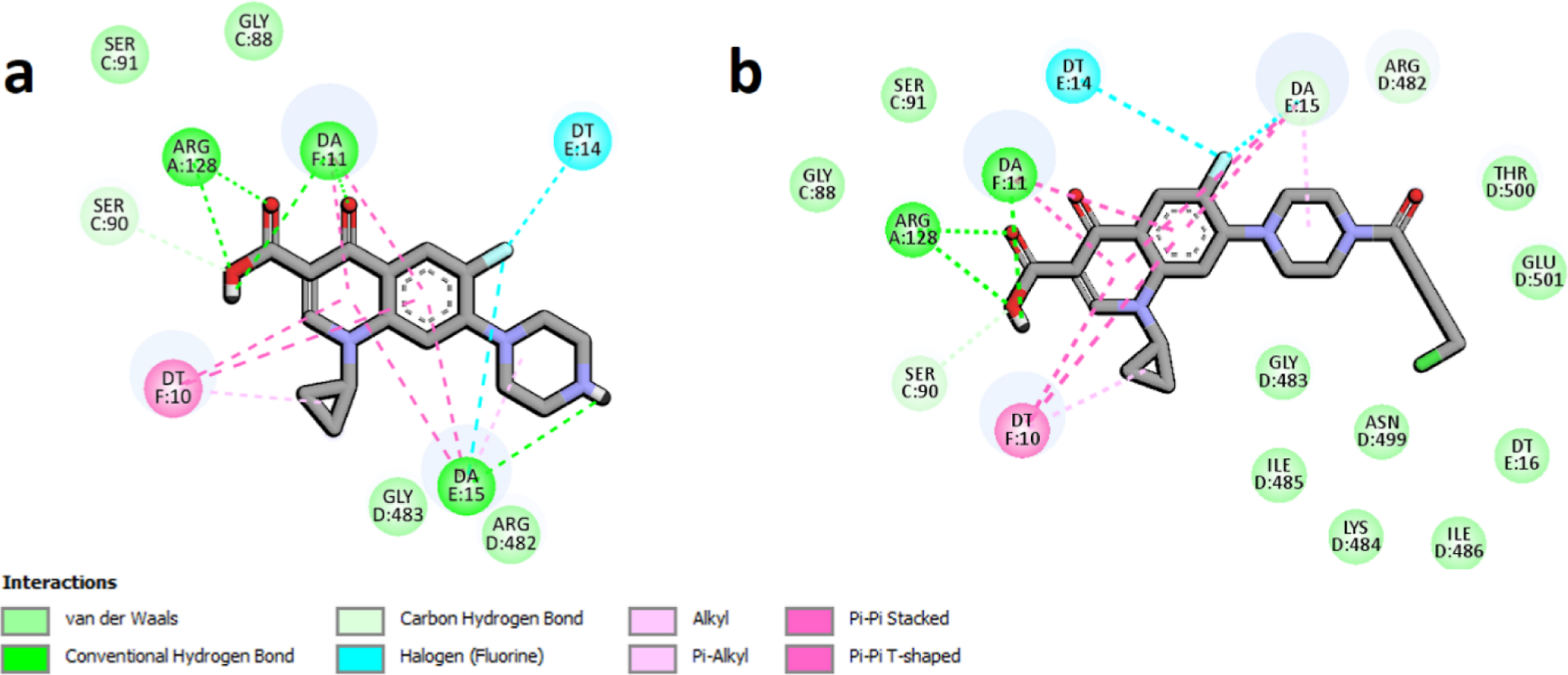
Interaction schemes generated for DNA topoisomerase IV and bound CP—panel (a) and compound **5**—panel (b). Colors presenting different interaction types are shown at the bottom. Figure generated using the BIOVIA Discovery Studio.

The binding modes of the most active antibacterial series **1–13** to both DNA gyrase and topoisomerase IV was investigated using molecular docking. The docking result analyses indicate that, for all the studied compounds, the binding of the CP scaffold is essential and very similar to that in the known CP–protein complexes.^48,49^ There are differences between particular compounds in the binding energy (and cluster sizes) but within the Autodock4 standard error (which is around 2.5 kcal/mol), see Table 5 for compound **5** and CP and Table S2 for all derivatives. Therefore, for the visualization purposes, we selected the chlorobutanoyl derivative (**5**), presenting the most potent inhibitory properties as compared to CP (see Table 4 showing IC_50_ values). Figure 6 shows the binding mode of compound **5** in comparison to that of CP. The interaction details are presented in Figures 7 and 8. As demonstrated in the figures, the chlorobutanoyl group of compound **5** forms several van der Waals interactions. Altogether, considering the general docking scores (Table 5) and interaction details (Figures 3–5), the effect of chlorobutanoyl group is weak (or negligible in comparison to CP) in light of the docking results.

**Table 5:** DNA Gyrase (PDB ID: 5BTC)^48^ and DNA Topoisomerase IV (PDB ID: 3RAD)^49^ Binding Data Based on the Docking Results for Compound **5** and **CP**^a^

	DNA gyrase		DNA topoisomerase IV	
compound	CS	BE (kcal/mol)	CS	BE (kcal/mol)
**5**	343	–9.12	662	–7.9100
**CP**	753	–7.26	698	–6.1700

a CS = number of members of the largest cluster calculated for 1000 docking runs using rmsd cutoff tolerance = 3 Å. BE = binding free energy values estimated using the AutoDock4 energy function for the representative ligand structure of the largest cluster.

#### 2.2.7 Cytotoxic Activity

To evaluate the chemotherapeutic profile of the synthesized series **1–21**, the in vitro antitumor activity has been determined for human prostate (PC3) cancer and normal HaCaT cell lines by using the MTT colorimetric assay (Table 6), including CP, doxorubicin, and cisplatin as reference drugs. While the unconjugated fluoroquinolone showed no activity toward PC3 cells as compared with almost all the synthesized compounds (IC_50_ > 100 μM), three derivatives exhibited high (2.02 μM) to moderate (15.7 μM) cytotoxic action. The most potent 2-chloroacetyl derivative **3** and the dimer **21**, bearing an undecanoyl linker, exhibited growth inhibitory activity at concentrations 2.02 and 4.8 μM, respectively. Their cytotoxic properties were then 6.5–2.75-fold stronger than those of cisplatin. Moreover, their selectivity indexes (SI) equaled 17.6 and 15.2, respectively. The cytotoxic activity of the butanoyl conjugate **15** was comparable to that of this chemotherapeutic (IC_50_ = 15.7 μM).

**Table 6:** Cytotoxic Activity (IC_50_, μM) of the Studied Compounds Estimated by the MTT Assay^a^

	PC3^d^		HaCaT^e^
	IC_50_^b^	SI^c^	IC_50_
**1**	68.7 ± 3.4	1.4	100.4 ± 1.2
**2**	85.2 ± 8.2	0.9	75.7 ± 7.5
**3**	2.02 ± 0.1	17.6	35.7 ± 8.2
**4**	51.6 ± 8.1	1.9	>100
**5**	97.2 ± 11.0	1.0	>100
**6**	107.9 ± 11.0	0.1	19.7 ± 2.8
**7**	>100	1	>100
**8**	39.6 ± 4.3	1.1	44.9 ± 9.0
**9**	69.1 ± 5.8	1.4	>100
**10**	84.1 ± 7.3	1.1	89.1 ± 2.1
**11**	81.7 ± 8.9	0.9	80.1 ± 8.1
**12**	29.7 ± 5.0	1.3	>100
**13**	33.7 ± 8.2	2.7	93.4 ± 10.1
**14**	63.4 ± 5.3	1.6	>100
**15**	15.7 ± 3.8	5.4	85.5 ± 9.2
**16**	68.8 ± 9.9	1.5	>100
**17**	88.4 ± 10.6	0.5	41.3 ± 5.2
**18**	>100	0.9	92.2 ± 8.7
**19**	71.4 ± 3.2	1.0	72.9 ± 5.7
**20**	69.9 ± 7.2	1.3	89.5 ± 5.9
**21**	4.8 ± 0.1	15.2	73.2 ± 10.1
CP	100.4 ± 3.6	2.2	222.1 ± 3.6
doxorubicin	0.3 ± 0.12	1.0	0.3 ± 0.11
cisplatin	13.2 ± 2.10	0.5	6.3 ± 0.70

a Data are expressed as mean SD.

b IC_50_ (μM)—the concentration of the compound that corresponds to a 50% growth inhibition of the cell line (as compared to the control) after 72 h of incubation with the individual compound.

c The SI (selectivity index) was calculated using the formula SI = IC_50_ for normal cell line/IC_50_ cancer cell line.

d Human metastatic prostate cancer (PC3).

e Human immortal keratinocyte cell line from adult human skin (HaCaT).

All microbiologically active conjugates, particularly **1–2**, **4**, **5**, **7–11**, and **14–16**, did not influence the growth of normal HaCaT cells. The results proved that the promising derivative **3** shares both strong antibacterial and cytotoxic properties, with the highest selectivity vs tumor cell lines.

#### 2.2.8 Antiproliferative Activity

**Figure 9:**
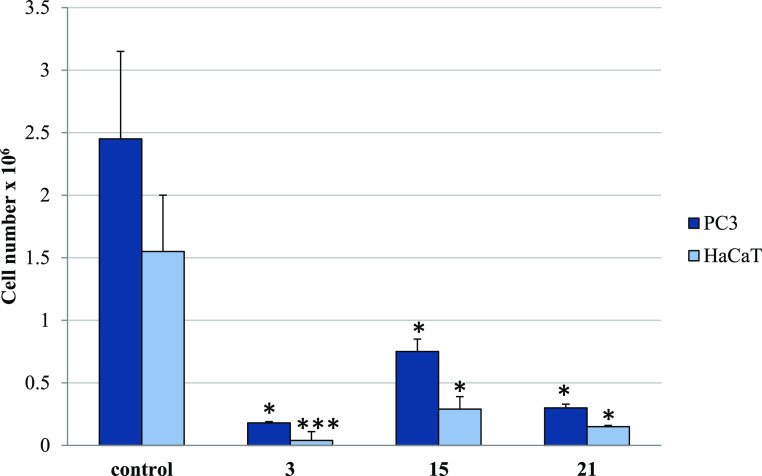
Trypan blue assay. The effect of compounds **3**, **15**, and **21** on cell number in PC3 and HaCaT cells. Cells were incubated for 72 h with tested compounds used in their IC_50_ concentrations; then, cells were harvested, stained with trypan blue, and analyzed using cell counter. Data are expressed as the mean ± SD. ***p ≤ 0.001, **p ≤ 0.002, and *p ≤ 0.01, as compared to the control.

**Figure 10:**
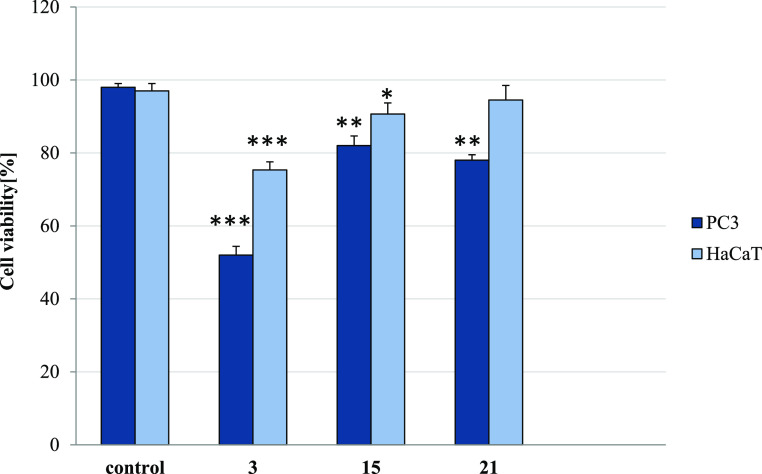
Trypan blue assay. The effect of compounds **3**, **15**, and **21** on viability in PC3 and HaCaT cells. Cells were incubated for 72 h with tested compounds used in their IC_50_ concentrations; then, cells were harvested, stained with trypan blue, and analyzed using the cell counter. Data are expressed as the mean ± SD. ***p ≤ 0.001, **p ≤ 0.01, and *p ≤ 0.05, as compared to the control.

To monitor cell proliferation in response to long-term conjugate treatment, the trypan-blue exclusion assay was further performed. The results obtained for derivatives **3**, **15**, and **21** have corresponded with their IC_50_ values measured by the MTT method. The compounds greatly reduced the number of live PC3 cells by 98.03–87.96% (Table S3 and Figure 9). Thus, they exerted a cytostatic effect on them, suppressing their growth and proliferation. However, the cytotoxic influence of derivative **3** on tested cancer cells was also observed, as it decreased the PC3 cell viability by 48% (Figure 10). Other CP-derived conjugates did not affect cell viability. In addition, while higher concentrations (35.7–85.5 μM) of the studied amides gave a moderate cytostatic effect in normal HaCaT cells (reduction of viable cells by 12.15–48.38%), they did not influence their viability. Moreover, compounds **3**, **15**, and **21** applied at their IC_50_ for PC3 cells (2.02, 15.7,, and 4.8 μM, respectively) had negligible impact on the number of live human keratinocytes (Figure S2).

#### 2.2.9 Apoptotic Activity

**Figure 11:**
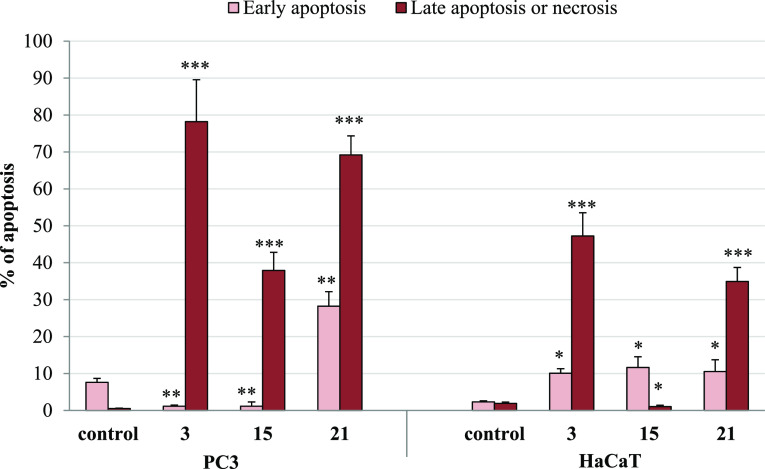
Effect of compounds **3**, **15**, and **21** on early and late apoptosis in PC3 and HaCaT cells. Cells were incubated for 72 h with the tested compounds applied in their IC_50_ concentrations; then, cells were harvested, stained with annexin V-FITC and PI, and analyzed using flow cytometry. Data are expressed as % of cells in the early stage of apoptosis, and as % of cells in the late stage of apoptosis or necrosis. Data are expressed as the mean ± SD. ***p ≤ 0.0001, **p ≤ 0.001, and *p ≤ 0.01 as compared to the control.

**Figure 12:**
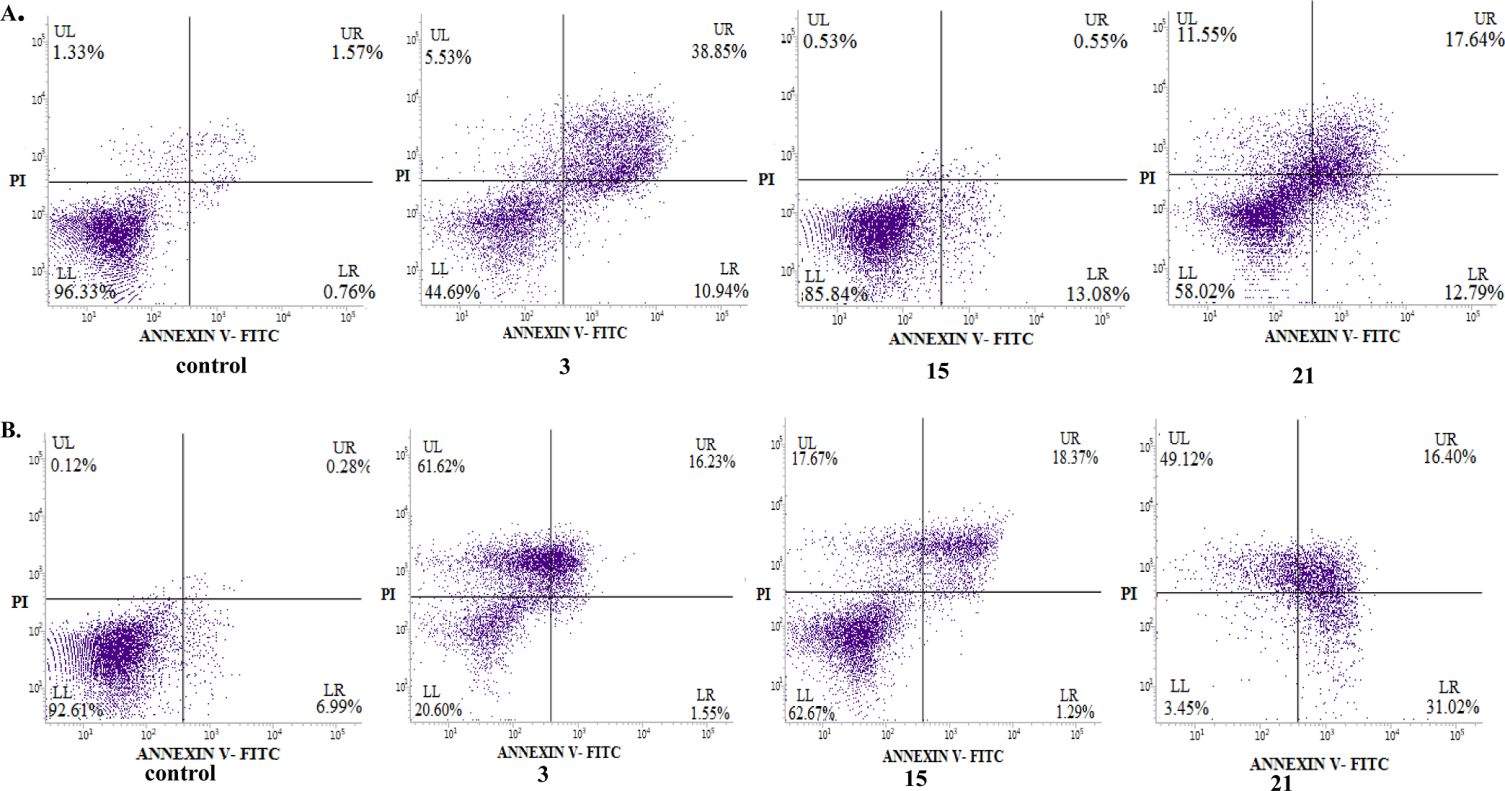
Effects of CP conjugates **3**, **15**, and **21** on early/late apoptosis or necrosis in (A) HaCaT and (B) PC3 cells detected with annexin V-FITC/PI by flow cytometry. Diagrams show the results of representative experiments. The lower right quadrant shows early apoptotic cells (annexin V-FITC-positive and PI-negative staining); the upper right and upper left quadrants represent the late stage of apoptotic or necrotic cells (annexin V-FITC-positive and PI-positive or annexin V-FITC-negative and PI-positive staining, respectively).

Apoptotic pathways have been currently important targets for the development of anticancer agents. To check the ability of new conjugates to induce apoptosis, the annexin V/PI double staining technique for analyzing PC3 cells treated with the most cytostatic compounds **3**, **15**, or **21** was used. As shown in Figures 11 and 12 and Table S4, after 72 h of treatment with conjugates **3** and **21**, the number of PC3 cells in late apoptosis or necrosis increased to 78.22 and 69.29%, respectively. In addition, the incubation with derivative **21** showed a significantly higher percentage of tumor cells in early apoptosis (28.22%), as compared to the control. The noticeable pro-apoptotic effect of the derivative **15** was also observed, with 37.64% of cells in late apoptosis or being necrotic. As expected, the test performed with HaCaT cells cultured with the studied compounds **3** and **21** revealed a late apoptosis/necrosis at the level of 47.23–34.95% as in untreated controls. The conjugate **15** did not indicate appreciable apoptosis-inducing action in normal cells.

#### 2.2.10 Inhibition of IL-6 Release

**Figure 13:**
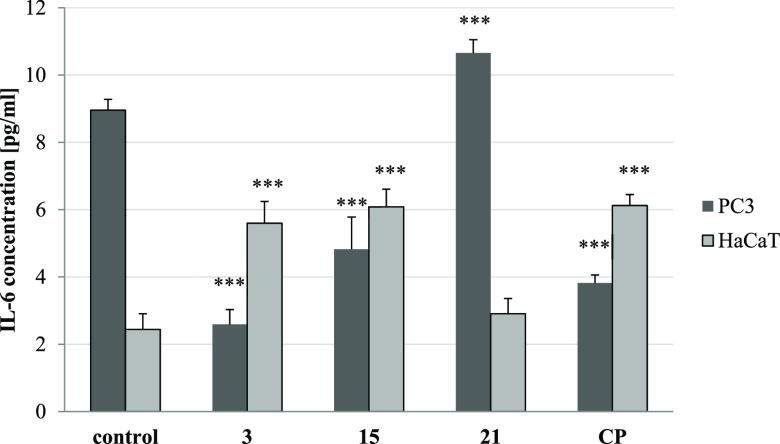
Effects of CP conjugates (**3**, **15**, and **21**) and CP alone on IL-6 levels measured by ELISA test. Data are expressed as the mean ± SD from three independent experiments performed in triplicate. ***p ≤ 0.001, **p ≤ 0.001, and *p ≤ 0.01, as compared to the control.

The predominant role of interleukin-6 (IL-6) in cancer is its key promotion of tumor growth. Since it was proved that CP and its derivatives can reduce the level of IL-6,^45^ both PC3 and HaCaT cells were treated with IC_50_ concentrations of the most cytotoxic derivatives of a series (**3**, **15**, and **21**). The strongest effect in PC3 cells was observed for the acetyl derivative **3**, which inhibited interleukin release 3.5 times, as compared to the control (Figure 13). That response was greater than the influence of CP alone, which diminished IL-6 secretion in these cells 2.4 times. Moreover, compound **15** decreased interleukin concentration 1.9 times. Similarly, as CP, conjugates **3** and **21** stimulated human keratinocytes to produce IL-6. No significant pro- or anti-inflammatory properties of derivative **15** was found.

#### 2.2.11 ROS Inducing Activity

Many chemotherapeutics increase the intracellular level of reactive oxygen species (ROS) and as a result disturb redox homeostasis and induce oxidative stress and ROS-mediated apoptosis in cancer cells. Doxorubicin and platinum complexes, such as cisplatin, target tumor mitochondria and elevate cellular ROS (particularly H_2_O_2_) production, which could lead to apoptosis and autophagy in cancer cells. The second reason of increased ROS generation is the inhibition of the cellular antioxidant system, including superoxide dismutase (SOD), peroxidases, and catalases.^50^ Therefore, we examined the ability of apoptosis-inducing agents (**3**, **15**, and **21**) to influence H_2_O_2_ production in both PC3 and HaCaT cell cultures.

**Figure 14:**
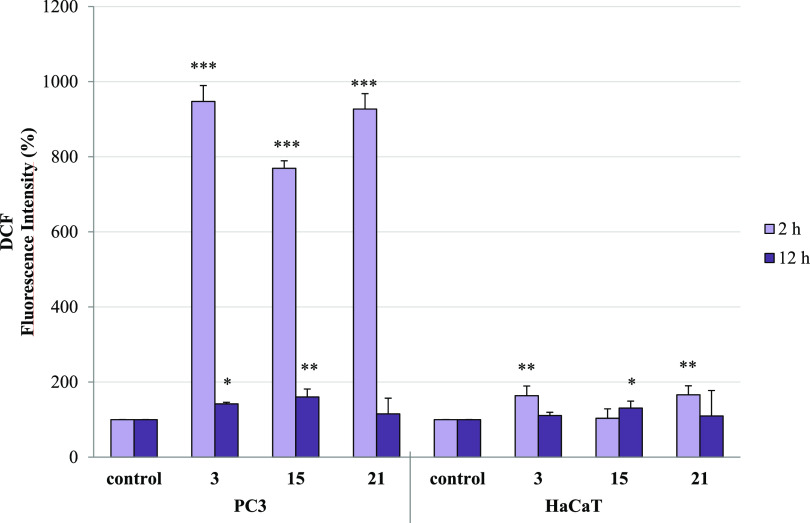
Effect of conjugates **3**, **15**, and **21** on ROS production in PC3 and HaCaT cells. Cells were incubated with tested compounds at their IC_50_ concentration for 2 and 12 h. Fluorescence intensity (FI) of the probe was measured by DCF (1 μM). The results are expressed as mean ± SD of three experiments, each of them performed in triplicate. ***p ≤ 0.0001, **p ≤ 0.001, and *p ≤ 0.01, as compared to the control.

As noticed, the conjugates applied in their IC_50_ concentrations affected differently ROS synthesis in both types of cells (Figures 14 and S3). After 2 h of compound treatment, the amount of ROS in PC3 cells increased 7.7–9.5 times and then measured after 12 h (or 72 h, data not shown), which was comparable to the control level. In contrast, normal HaCaT cells were less vulnerable to the presence of CP derivatives, and the intensity of ROS generation in these cells, independently of treatment time, was nearly equivalent to the control probe. One can conclude that the increased level of H_2_O_2_ may lead to activation of apoptosis or necrosis in PC3 cells. However, compounds **3** and **21** induced HaCaT cell death probably by a different, ROS-independent mechanism.

## 3 Conclusions

The current study describes the synthesis of novel CP derivatives (**1–21**), obtained by modification of the C-7 piperazinyl moiety with halogenated acyl chlorides. Conjugates were evaluated for their in vitro growth inhibitory activity against a panel of Gram-positive and Gram-negative bacteria. Many compounds (**1–11** and **14–16**) exhibited a potent antistaphylococcal action against standard strains, with IC_50_ values between 0.05 and 0.4 μM, which were 1.25–10 folds more effective than unconjugated CP. In addition, the MIC values of the most potent amides (**2–9**, **14**, and **15**) toward selected clinical *Staphylococci* were in the range from 0.1 to 0.5 μg/mL, being 1.25–5-fold lower than for the parental drug. Alkanoyl derivatives **5**, **10**, and **11** acted more effectively toward hospital *E. coli* and *E. cloacae* strains, as compared to CP alone. During 18 h of observation, conjugates **5**, **10**, and **11** fully inhibited *P. aeruginosa* and *E. coli* cell growth at doses of ≥0.2 and ≥0.025 μg/mL, respectively. At lower concentrations (0.1–0.003 μg/mL), the compounds significantly disturbed the bacterial cell metabolism, as they extended the lag and elongated/shifted the exponential phases or prolonged the stationary phase. Similarly as CP, compounds **5** and **11** at doses of 2–4 MIC moderately eradicated the biofilm formed by selected Gram-negative pathogens. The other advantage of the synthesized compounds (**2**, **5–7**, and **10–12**) is their antitubercular activity, being at least equipotent to CP and 64–128 higher than the first-line antimycobacterial agents, such as INH, RMP, SM, and EMB, especially against the multidrug-resistant *M. tuberculosis* Spec. 210. As shown, the antibacterial profile of compounds **1**, **2**, **5**, **6**, **10**, and **11** appears to correlate with their ability to inhibit both *S. aureus* DNA gyrase and topoisomerase IV. The binding mode of target compounds **1–13** to both topoisomerases is comparable to that of the known CP–protein complexes. Moreover, the MTT assay against PC3 cells indicated that derivatives **3** and **21** have exceptional cytotoxic activity (IC_50_ 2.02–4.8 μM), stronger than cisplatin, with high selectivity over normal HaCaT cells. These amides almost completely blocked the overall proliferation rate in PC3 cells (by 98.03–93.78%), significantly increasing their death rate, without reduction of HaCaT cell viability. The flow cytometry analysis demonstrated that both CP conjugates greatly induced apoptosis in prostate cancer cells, giving 78.22–69.29% cells in late apoptosis or necrosis. Conjugates **3** and **15** affected the PC3 cell growth by a 3.5–1.9-fold reduction of the interleukin level. As estimated, compounds **3**, **15**, and **21** also elevated intracellular ROS (particularly H_2_O_2_) generation, which could explain their pro-apoptotic influence on cancer cells. Modifications of the CP structure have increased its antibacterial spectrum and cytotoxic potency against cancer cells, retaining the relative safety against the normal cell line. The performed studies allowed to select derivative **3**, which shares both strong antibacterial and antitumor properties, with an in-depth mechanism of its cytotoxic activity.

## 4 Materials and Methods

### 4.1 Chemistry

Dichloromethane and methanol were supplied from Sigma-Aldrich. CP (98%), 3-chloropropionyl chloride (98%), and 3-bromopropionyl chloride (95%) were purchased from Acros Organics. Chloroacetyl chloride (98%), bromoacetyl chloride (≥95%), 4-chlorobutyryl chloride (99%), 5-chlorovaleric acid (≥98%), and 11-bromoundecanoic acid (99%) were purchased from Sigma-Aldrich. 5-Bromovaleryl chloride (98%) and 6-bromohexanoyl chloride (97%) were purchased from Alfa Aesar company. 6-Chlorohexanoic acid (95%), 3,3,3-trifluoropropionic acid (98%), 4,4,4-trifluorobutanoic acid (98%), and 5,5,5-trifluoropentanoic acid (97%) were purchased from Fluorochem Ltd. All other chemicals were of analytical grade and were used without any further purification. The NMR spectra were recorded on a Bruker AVANCE spectrometer operating at 500 MHz for ^1^H NMR and at 125 MHz for ^13^C NMR. The spectra were recorded in CDCl_3_, CDCl_3_/CD_3_OD, 9:1 mixture, or DMSO-*d*_6_ and are given as δ values (in ppm) relative to TMS. Mass spectral ESI measurements were carried out on an LCT Micromass TOF HiRes apparatus. TLC analyses were performed on silica gel plates (Merck Kiesegel GF254) and visualized using UV light or iodine vapor. Column chromatography was carried out at atmospheric pressure using Silica Gel 60 (230–400 mesh, Merck) and using dichloromethane/methanol (0–8%) mixture as an eluent.

### 4.2 General Procedure for the Synthesis of CP Conjugates **1–21**

To a magnetically stirred suspension of CP (1.00 g; 3.02 mmol) and triethylamine (0.84 mL; 6.04 mmol) in dry CH_2_Cl_2_ (120 mL), a solution of halogenated carboxylic acid chloride (3.02 mmol) in dry CH_2_Cl_2_ (5 mL) was dropped over 5 min at 2–5 °C. After 30 min, the cooling bath was removed, and the reaction mixture was stirred at room temperature for 3 h. Next, water (50 mL) and 3% HCl (aq) solution were added to get pH equaled 3–4. After separation of the phases, the water layer was extracted with CH_2_Cl_2_ (50 mL). The combined organic layers were washed with water (50 mL) and dried over anhydrous Na_2_SO_4_. Then, the solvent was evaporated under reduced pressure, and the product was isolated using column chromatography on silica gel, with the CH_2_Cl_2_/MeOH mixture (0–8% MeOH) as an eluent system. The monoamides were eluted using 1–4% of methanol, but the elution of dimers required a stronger mobile phase (5–10% of methanol in CH_2_Cl_2_). For the synthesis of compounds **6**, **7**, and **13–16**, the appropriate fatty acid chlorides were freshly synthesized from acids in the reaction with oxalyl chloride (1:2 molar ratio, CH_2_Cl_2_, 0–25 °C, 3 h).

#### 4.2.1 7-(4-Acryloyl-piperazin-1-yl)-1-cyclopropyl-6-fluoro-4-oxo-1,4-dihydro-quinoline-3-carboxylic Acid (**1**)

White solid, 0.40 g (34%). mp 248.5–249.8 °C). ^1^H NMR (CDCl_3_/CD_3_OD, 9:1 mixture, 500 MHz): δ (ppm) 1.22–1.25 (m, 2H), 1.42–1.46 (m, 2H), 3.41 (br s, 2H), 3.50 (br s, 2H), 3.59–3.63 (m, 1H), 3.86 (br s, 2H), 3.94 (br s, 2H)), 5.82 (dd, *J* = 10.5 Hz, 1.5 Hz, 1H), 6.34 (dd, *J* = 16.5 Hz, 10.5 Hz, 1H), 6.65 (dd, *J* = 16.5 Hz, 1.5 Hz, 1H), 7.42 (d, *J* = 7.5 Hz, 1H), 7.98 (d, *J* = 13.0 Hz, 1H), 8.75 (s, 1H). ^13^C NMR (CDCl_3_/CD_3_OD, 9:1 mixture, 125 MHz): δ (ppm) 8.0 (2 × C), 35.4, 41.5, 45.5, 49.0 (d, ^4^*J*_*C–F*_ = 5.0 Hz), 50.0 (d, ^4^*J*_*C–F*_ = 5.0 Hz), 105.2 (d, ^3^*J*_*C–F*_ = 3.8 Hz), 107.5, 112.2 (d, ^2^*J*_*C–F*_ = 22.5 Hz), 120.0 (d, ^3^*J*_*C–F*_ = 7.5 Hz), 126.7, 128.8, 138.9, 145.2 (d, ^2^*J*_*C–F*_ = 11.3 Hz), 147.6, 153.5 (d, ^1^*J*_*C–F*_ = 248.7 Hz), 165.9, 167.3, 176.9 (d, ^4^*J*_*C–F*_ = 2.5 Hz). HRMS (ESI) *m*/*z*: 386.1534 (calcd for C_20_H_21_FN_3_O_4_ [M + H]^+^, 386.1516).

#### 4.2.2 7-(4-Cyclopropanecarbonyl-piperazin-1-yl)-1-cyclopropyl-6-fluoro-4-oxo-1,4-dihydro-quinoline-3-carboxylic Acid (**2**)

White solid, 60 mg (5%). mp 280.0–280.7 °C. ^1^H NMR (CDCl_3_/CD_3_OD, 9:1 mixture, 500 MHz): δ (ppm) 0.84–0.88 (m, 2H), 1.02–1.05 (m, 2H), 1.21–1.25 (m, 2H), 1.41–1.45 (m, 2H), 1.80–1.85 (m, 1H), 3.34 (br s, 2H), 3.45 (br s, 2H), 3.57–3.67 (m, 1H), 3.88 (br s, 2H), 3.97 (br s, 2H), 7.39 (d, *J* = 7.0 Hz, 1H), 7.94 (d, *J* = 13.0 Hz, 1H), 8.71 (s, 1H). ^13^C NMR (CDCl_3_/CD_3_OD, 9:1 mixture, 125 MHz): δ (ppm) 7.7 (2 × C), 8.1 (2 × C), 10.1, 35.4, 41.6, 45.2, 49.2, 50.1, 105.0 (d, ^3^*J*_*C–F*_ = 3.8 Hz), 107.6, 112.2 (d, ^2^*J*_*C–F*_ = 22.5 Hz), 119.9 (d, ^3^*J*_*C–F*_ = 7.5 Hz), 138.9, 145.3 (d, ^2^*J*_*C–F*_ = 10.0 Hz), 147.5, 153.5 (d, ^1^*J*_*C–F*_ = 248.7 Hz), 167.0, 172.6, 176.8 (d, ^4^*J*_*C–F*_ = 2.5 Hz). HRMS (ESI) *m*/*z*: 386.1534 (calcd for C_20_H_21_FN_3_O_4_ [M + H]^+^, 386.1516).

#### 4.2.3 7-[4-(2-Chloroacetyl)-piperazin-1-yl]-1-cyclopropyl-6-fluoro-4-oxo-1,4-dihydro-quinoline-3-carboxylic Acid (**3**)

Pale beige solid, 0.42 g (34%). mp 258.7–259.3 °C. ^1^H NMR (CDCl_3_/CD_3_OD, 9:1 mixture, 500 MHz): δ (ppm) 1.22–1.26 (m, 2H), 1.42–1.46 (m, 2H), 3.35–3.38 (m, 2H), 3.44 (t, *J* = 5.0 Hz, 2H), 3.58–3.62 (m, 1H), 3.81 (t, *J* = 5.0 Hz, 2H), 3.89 (t, *J* = 5.0 Hz, 2H), 4.17 (s, 2H), 7.43 (d, *J* = 7.0 Hz, 1H), 8.05 (d, *J* = 12.5 Hz, 1H), 8.80 (s, 1H). ^13^C NMR (CDCl_3_/CD_3_OD, 9:1 mixture, 125 MHz): δ (ppm) 8.1 (2 × C), 35.4, 40.4, 41.7, 46.0, 49.0 (d, ^4^*J*_*C–F*_ = 5.0 Hz), 49.8 (d, ^4^*J*_*C–F*_ = 5.0 Hz), 105.3 (d, ^3^*J*_*C–F*_ = 2.5 Hz), 107.7, 112.5 (d, ^2^*J*_*C–F*_ = 23.7 Hz), 120.4 (d, ^3^*J*_*C–F*_ = 8.7 Hz), 138.9, 145.2 (d, ^2^*J*_*C–F*_ = 10.0 Hz), 147.8, 153.5 (d, ^1^*J*_*C–F*_ = 248.7 Hz), 165.6, 167.4, 177.0 (d, ^4^*J*_*C–F*_ = 2.5 Hz). HRMS (ESI) *m*/*z*: 400.1650 (calcd for C_21_H_23_FN_3_O_4_ [M + H]^+^, 400.1673).

#### 4.2.4 7-[4-(3-Chloropropionyl)-piperazin-1-yl]-1-cyclopropyl-6-fluoro-4-oxo-1,4-dihydro-quinoline-3-carboxylic Acid (**4**)

White solid, 0.60 g (47%). mp 257.6–258.2 °C. ^1^H NMR (CDCl_3_/CD_3_OD, 9:1 mixture, 500 MHz): δ (ppm) 1.22–1.25 (m, 2H), 1.42–1.46 (m, 2H), 2.91 (t, *J* = 6.5 Hz, 2H), 3.35 (t, *J* = 5.0 Hz, 2H), 3.41 (t, *J* = 5.0 Hz, 2H), 3.58–3.63 (m, 1H), 3.77 (t, *J* = 5.0 Hz, 2H), 3.87 (t, *J* = 7.0 Hz, 2H), 3.90 (t, *J* = 5.0 Hz, 2H), 7.41 (d, *J* = 7.0 Hz, 1H), 8.01 (d, *J* = 13.0 Hz, 1H), 8.77 (s, 1H). ^13^C NMR (CDCl_3_/CD_3_OD, 9:1 mixture, 125 MHz): δ (ppm) 8.0 (2 × C), 35.4, 35.7, 39.6, 41.3, 45.3, 49.2 (d, ^4^*J*_*C–F*_ = 5.0 Hz), 49.9 (d, ^4^*J*_*C–F*_ = 5.6 Hz), 105.2 (d, ^3^*J*_*C–F*_ = 3.8 Hz), 107.6, 112.4 (d, ^2^*J*_*C–F*_ = 23.8 Hz), 120.1 (d, ^3^*J*_*C–F*_ = 7.5 Hz), 138.9 (d, ^4^*J*_*C–F*_ = 0.6 Hz), 145.2 (d, ^2^*J*_*C–F*_ = 10.0 Hz), 147.7, 153.5 (d, ^1^*J*_*C–F*_ = 250.0 Hz), 167.3, 168.7, 176.9 (d, ^4^*J*_*C–F*_ = 2.5 Hz). HRMS (ESI) *m*/*z*: 422.1269 (calcd for C_20_H_22_ClFN_3_O_4_ [M + H]^+^, 422.1283).

#### 4.2.5 7-[4-(4-Chlorobutyryl)-piperazin-1-yl]-1-cyclopropyl-6-fluoro-4-oxo-1,4-dihydro-quinoline-3-carboxylic Acid (**5**)

White solid, 0.75 g (57%). mp 254.7–255.3 °C. ^1^H NMR (CDCl_3_/CD_3_OD, 9:1 mixture, 500 MHz): δ (ppm) 1.21–1.25 (m, 2H), 1.41–1.45 (m, 2H), 2.14–2.19 (m, 2H), 2.61 (t, *J* = 7.0 Hz, 2H), 3.33 (t, *J* = 5.0 Hz, 2H), 3.40 (t, *J* = 5.0 Hz, 2H), 3.57–3.62 (m, 1H), 3.68 (t, *J* = 6.0 Hz, 2H), 3.78 (t, *J* = 5.0 Hz, 2H), 3.88 (t, *J* = 5.0 Hz, 2H), 7.40 (d, *J* = 7.0 Hz, 1H), 7.99 (d, *J* = 13.0 Hz, 1H), 8.75 (s, 1H). ^13^C NMR (CDCl_3_/CD_3_OD, 9:1 mixture, 125 MHz): δ (ppm) 8.1 (2 × C), 27.6, 29.6, 35.7, 41.2, 44.6, 45.2, 49.2 (d, ^4^*J*_*C–F*_ = 3.8 Hz), 49.9 (d, ^4^*J*_*C–F*_ = 5.0 Hz), 105.1 (d, ^3^*J*_*C–F*_ = 2.5 Hz), 107.7, 112.3 (d, ^2^*J*_*C–F*_ = 27.5 Hz), 120.1 (d, ^3^*J*_*C–F*_ = 8.8 Hz), 138.9 (d, ^4^*J*_*C–F*_ = 0.6 Hz), 145.3 (d, ^2^*J*_*C–F*_ = 10.0 Hz), 147.6, 153.5 (d, ^1^*J*_*C–F*_ = 250.0 Hz), 167.2, 170.7, 176.9 (d, ^4^*J*_*C–F*_ = 2.5 Hz). HRMS (ESI) *m*/*z*: 458.1242 (calcd for C_21_H_23_ClFN_3_O_4_Na [M + Na]^+^, 458.1259).

#### 4.2.6 7-[4-(5-Chloropentanoyl)-piperazin-1-yl]-1-cyclopropyl-6-fluoro-4-oxo-1,4-dihydro-quinoline-3-carboxylic Acid (**6**)

White solid, 1.0 g (73%). mp 234.7–235.6 °C. ^1^H NMR (CDCl_3_/CD_3_OD, 9:1 mixture, 500 MHz): δ (ppm) 1.22–1.25 (m, 2H), 1.41–1.45 (m, 2H), 1.81–1.91 (m, 4H), 2.46 (t, *J* = 7.0 Hz, 2H), 3.33 (t, *J* = 5.0 Hz, 2H), 3.39–3.41 (m, 2H), 3.57–3.64 (m, 3H), 3.76 (t, *J* = 5.0 Hz, 2H), 3.87 (t, *J* = 5.0 Hz, 2H), 7.41 (d, *J* = 7.0 Hz, 1H), 7.98 (d, *J* = 13.0 Hz, 1H), 8.75 (s, 1H). ^13^C NMR (CDCl_3_/CD_3_OD, 9:1 mixture, 125 MHz): δ (ppm) 8.0 (2 × C), 22.3, 31.8, 32.2, 35.4, 41.1, 44.5, 45.3, 49.2 (d, ^4^*J*_*C–F*_ = 3.8 Hz), 50.0 (d, ^4^*J*_*C–F*_ = 6.3 Hz), 105.2 (d, ^3^*J*_*C–F*_ = 2.5 Hz), 107.6, 112.3 (d, ^2^*J*_*C–F*_ = 22.5 Hz), 120.0 (d, ^3^*J*_*C–F*_ = 7.5 Hz), 138.9, 145.3 (d, ^2^*J*_*C–F*_ = 11.3 Hz), 147.7, 153.5 (d, ^1^*J*_*C–F*_ = 250.0 Hz), 167.3, 171.5, 176.9 (d, ^4^*J*_*C–F*_ = 2.5 Hz). HRMS (ESI) *m*/*z*: 472.1433 (calcd for C_22_H_25_ClFN_3_O_4_Na [M + Na]^+^, 472.1415).

#### 4.2.7 7-[4-(6-Chlorohexanoyl)-piperazin-1-yl]-1-cyclopropyl-6-fluoro-4-oxo-1,4-dihydro-quinoline-3-carboxylic Acid (**7**)

White solid, 0.73 g (52%). mp 189.6–190.8 °C. ^1^H NMR (CDCl_3_, 500 MHz): δ (ppm) 1.20–1.23 (m, 2H), 1.39–1.43 (m, 2H), 1.50–1.56 (m, 2H), 1.68–1.75 (m, 2H), 1.80–1.86 (m, 2H), 2.42 (t, *J* = 7.5 Hz, 2H), 3.30 (t, *J* = 5.0 Hz, 2H), 3.37 (t, *J* = 4.5 Hz, 2H), 3.53–3.58 (m, 3H), 3.73 (t, *J* = 4.5 Hz, 2H), 3.88 (t, *J* = 4.5 Hz, 2H), 7.37 (d, *J* = 7.0 Hz, 1H), 8.03 (d, *J* = 13.0 Hz, 1H), 8.75 (s, 1H), 14.90 (s, 1H). ^13^C NMR (CDCl_3_, 125 MHz): δ (ppm) 8.0 (2 × C), 35.4, 35.4, 35.7 (2C), 39.6 (2C), 41.3, 45.3, 49.2, 49.9 (d, ^4^*J*_*C–F*_ = 6.3 Hz), 105.2 (d, ^3^*J*_*C–F*_ = 3.8 Hz), 107.6, 112.3 (d, ^2^*J*_*C–F*_ = 23.8 Hz), 120.1 (d, ^3^*J*_*C–F*_ = 7.5 Hz), 138.9, 145.2 (d, ^2^*J*_*C–F*_ = 10.0 Hz), 147.7, 153.5 (d, ^1^*J*_*C–F*_ = 250.0 Hz), 167.3, 168.7, 176.9 (d, ^4^*J*_*C–F*_ = 2.5 Hz). HRMS (ESI) *m*/*z*: 486.1550 (calcd for C_23_H_27_ClFN_3_O_4_Na [M + Na]^+^, 486.1572).

#### 4.2.8 7-[4-(2-Bromoacetyl)-piperazin-1-yl]-1-cyclopropyl-6-fluoro-4-oxo-1,4-dihydro-quinoline-3-carboxylic Acid (**8**)

White solid, 0.38 g (28%). mp 261.8–263.1 °C. ^1^H NMR (DMSO-*d*_6_, 500 MHz): δ (ppm) 1.16–1.20 (m, 2H), 1.30–1.34 (m, 2H), 3.39–3.41 (m, 4H), 3.70–3.73 (m, 4H), 3.80–3.84 (m, 1H), 4.22 (s, 2H), 7.58 (d, *J* = 7.0 Hz, 1H), 7.92 (d, *J* = 3.0 Hz, 1H), 8.66 (s, 1H). ^13^C NMR (DMSO-*d*_6_, 125 MHz): δ (ppm) 7.6, 7.6, 27.8, 35.9, 41.3, 41.6, 49.0 (d, ^4^*J*_*C–F*_ = 3.8 Hz), 49.3 (d, ^4^*J*_*C–F*_ = 5.0 Hz), 106.7, 106.7, 111.0 (d, ^2^*J*_*C–F*_ = 23.8 Hz), 118.9 (d, ^3^*J*_*C–F*_ = 7.5 Hz), 139.1, 144.7 (d, ^2^*J*_*C–F*_ = 10.0 Hz), 148.1, 152.9 (d, ^1^*J*_*C–F*_ = 248.8 Hz), 165.0, 165.9, 176.3 (d, ^4^*J*_*C–F*_ = 2.5 Hz). HRMS (ESI) *m*/*z*: 452.0645 (calcd for C_19_H_20_BrFN_3_O_4_ [M + H]^+^, 452.0621).

#### 4.2.9 7-[4-(3-Bromopropionyl)-piperazin-1-yl]-1-cyclopropyl-6-fluoro-4-oxo-1,4-dihydro-quinoline-3-carboxylic Acid (**9**)

White solid, 0.45 g (32%). mp 244.2–245.0 °C. ^1^H NMR (CDCl_3_/CD_3_OD, 9:1 mixture, 500 MHz): δ (ppm) 1.20–1.25 (m, 2H), 1.39–1.45 (m, 2H), 3.02 (t, *J* = 7.0 Hz, 2H), 3.35 (t, *J* = 5.0 Hz, 2H), 3.42 (t, *J* = 5.0 Hz, 2H), 3.58–3.62 (m, 1H), 3.69 (t, *J* = 7.0 Hz, 2H), 3.77 (t, *J* = 5.0 Hz, 2H), 3.90 (t, *J* = 5.0 Hz, 2H), 7.39 (d, *J* = 7.0 Hz, 1H), 7.96 (d, *J* = 12.5 Hz, 1H), 8.73 (s, 1H). ^13^C NMR (CDCl_3_/CD_3_OD, 9:1 mixture, 125 MHz): δ (ppm) 8.1 (2 × C), 26.9, 35.4, 35.9, 41.3, 45.2, 49.2, 49.9 (d, ^4^*J*_*C–F*_ = 5.0 Hz), 105.2 (d, ^3^*J*_*C–F*_ = 2.5 Hz), 107.6, 112.3 (d, ^2^*J*_*C–F*_ = 27.5 Hz), 120.1 (d, ^3^*J*_*C–F*_ = 7.5 Hz), 138.9, 145.2 (d, ^2^*J*_*C–F*_ = 10.0 Hz), 147.6, 153.5 (d, ^1^*J*_*C–F*_ = 250.0 Hz), 167.1, 168.9, 176.9 (d, ^4^*J*_*C–F*_ = 2.5 Hz). HRMS (ESI) *m*/*z*: 466.0792 (calcd for C_20_H_22_BrFN_3_O_4_ [M + H]^+^, 466.0778).

#### 4.2.10 7-[4-(4-Bromobutyryl)-piperazin-1-yl]-1-cyclopropyl-6-fluoro-4-oxo-1,4-dihydro-quinoline-3-carboxylic Acid (**10**)

White solid, 0.59 g (41%). mp 247.0–247.7 °C. ^1^H NMR (CDCl_3_/CD_3_OD, 9:1 mixture, 500 MHz): δ (ppm) 1.21–1.25 (m, 2H), 1.41–1.45 (m, 2H), 2.22–2.27 (m, 2H), 2.61 (t, *J* = 7.0 Hz, 2H), 3.33 (t, *J* = 5.0 Hz, 2H), 3.41 (t, *J* = 5.0 Hz, 2H), 3.55 (t, *J* = 6.0 Hz, 2H), 3.65–3.72 (m, 1H), 3.78 (t, *J* = 5.0 Hz, 2H), 3.88 (t, *J* = 5.0 Hz, 2H), 7.38 (d, *J* = 6.5 Hz, 1H), 7.95 (d, *J* = 13.0 Hz, 1H), 8.71 (s, 1H). ^13^C NMR (CDCl_3_/CD_3_OD, 9:1 mixture, 125 MHz): δ (ppm) 8.1 (2 × C), 27.7, 30.9, 33.7, 35.4, 41.2, 45.2, 49.3 (d, ^4^*J*_*C–F*_ = 3.8 Hz), 49.9 (d, ^4^*J*_*C–F*_ = 5.0 Hz), 105.1 (d, ^3^*J*_*C–F*_ = 3.8 Hz), 107.7, 112.3 (d, ^2^*J*_*C–F*_ = 22.5 Hz), 120.0 (d, ^3^*J*_*C–F*_ = 8.8 Hz), 138.9, 145.3 (d, ^2^*J*_*C–F*_ = 10.0 Hz), 147.6, 153.5 (d, ^1^*J*_*C–F*_ = 250.0 Hz), 167.0, 170.5, 176.8 (d, ^4^*J*_*C–F*_ = 2.5 Hz). HRMS (ESI) *m*/*z*: 480.0954 (calcd for C_21_H_24_BrFN_3_O_4_ [M + H]^+^, 480.0934).

#### 4.2.11 7-[4-(5-Bromopentanoyl)-piperazin-1-yl]-1-cyclopropyl-6-fluoro-4-oxo-1,4-dihydro-quinoline-3-carboxylic Acid (**11**)

White solid, 1.15 g (77%). mp 225.7–226.2 °C. ^1^H NMR (CDCl_3_, 500 MHz): δ (ppm) 1.22–1.24 (m, 2H), 1.40–1.44 (m, 2H), 1.81–1.87 (m, 2H), 1.93–1.98 (m, 2H), 2.45 (t, *J* = 7.5 Hz, 2H), 3.32 (t, *J* = 5.0 Hz, 2H), 3–39 (t, *J* = 5.0 Hz, 2H), 3.46 (t, *J* = 6.5 Hz, 2H), 3.56–3.60 (m, 1H), 3.75 (t, *J* = 5.0 Hz, 2H), 3.87 (t, *J* = 5.0 Hz, 2H), 7.37 (d, *J* = 7.5 Hz, 1H), 7.96 (d, *J* = 13.0 Hz, 1H), 8.71 (s, 1H). ^13^C NMR (CDCl_3_, 125 MHz): δ (ppm) 8.2 (2 × C), 23.6, 32.1, 32.1, 33.3, 35.3, 41.1, 45.3, 49.3 (d, ^4^*J*_*C–F*_ = 3.8 Hz), 50.1 (d, ^4^*J*_*C–F*_ = 5.0 Hz), 105.1 (d, ^3^*J*_*C–F*_ = 3.8 Hz), 107.8, 112.3 (d, ^2^*J*_*C–F*_ = 25.0 Hz), 120.1 (d, ^3^*J*_*C–F*_ = 7.5 Hz), 138.9, 145.3 (d, ^2^*J*_*C–F*_ = 10.0 Hz), 147.6, 153.5 (d, ^1^*J*_*C–F*_ = 250.0 Hz), 166.9, 171.1, 176.9 (d, ^4^*J*_*C–F*_ = 5.0 Hz). HRMS (ESI) *m*/*z*: 516.0931 (calcd for C_22_H_25_BrFN_3_O_4_Na [M + Na]^+^, 516.0910).

#### 4.2.12 7-[4-(6-Bromohexanoyl)-piperazin-1-yl]-1-cyclopropyl-6-fluoro-4-oxo-1,4-dihydro-quinoline-3-carboxylic Acid (**12**)

White solid, 0.95 g (62%). mp 187.3–189.1 °C. ^1^H NMR (CDCl_3_, 500 MHz): δ (ppm) 1.20–1.23 (m, 2H), 1.39–1.43 (m, 2H), 1.50–1.56 (m, 2H), 1.69–1.75 (m, 2H), 1.89–1.94 (m, 2H), 2.42 (t, *J* = 7.5 Hz, 2H), 3.32 (t, *J* = 5.0 Hz, 2H), 3.39 (t, *J* = 5.0 Hz, 2H), 3.44 (t, *J* = 8.0 Hz, 2H), 3.56–3.60 (m, 1H), 3.74 (t, *J* = 5.0 Hz, 2H), 3.88 (t, *J* = 5.0 Hz, 2H), 7.35 (d, *J* = 7.0 Hz, 1H), 7.91 (d, *J* = 12.5 Hz, 1H), 8.66 (s, 1H), 14.89 (s, 1H). ^13^C NMR (CDCl_3_, 125 MHz): δ (ppm) 8.2 (2 × C), 24.2, 27.9, 32.5, 32.8, 33.7, 35.3, 41.1, 45.2, 49.4 (d, ^4^*J*_*C–F*_ = 3.8 Hz), 50.1 (d, ^4^*J*_*C–F*_ = 5.0 Hz), 105.1 (d, ^3^*J*_*C–F*_ = 2.5 Hz), 107.9, 112.2 (d, ^2^*J*_*C–F*_ = 23.8 Hz), 119.9 (d, ^3^*J*_*C–F*_ = 8.8 Hz), 138.9, 145.3 (d, ^2^*J*_*C–F*_ = 10.0 Hz), 147.4, 153.5 (d, ^1^*J*_*C–F*_ = 250.0 Hz), 166.6, 171.3, 176.8 (d, ^4^*J*_*C–F*_ = 2.5 Hz). HRMS (ESI) *m*/*z*: 530.1046 (calcd for C_23_H_27_BrFN_3_O_4_Na [M + Na]^+^, 530.1067).

#### 4.2.13 7-[4-(11-Bromoundekanoyl)-piperazin-1-yl]-1-cyclopropyl-6-fluoro-4-oxo-1,4-dihydro-quinoline-3-carboxylic Acid (**13**)

Beige solid, 1.50 g (86%). mp 183.9–185.0 °C. ^1^H NMR (CDCl_3_, 300 MHz): δ (ppm) 1.18–1.24 (m, 2H), 1.30–1.47 (m, 14H), 1.62–1.71 (m, 2H), 1.80–1.90 (m, 2H), 2.39 (t, *J* = 7.5 Hz, 2H), 3.32 (t, *J* = 5.1 Hz, 2H), 3.37–3.43 (m, 4H), 3.54–3.62 (m, 1H), 3.74 (t, *J* = 4.8 Hz, 2H), 3.88 (t, *J* = 4.8 Hz, 2H), 7.35 (d, *J* = 6.9 Hz, 1H), 7.90 (d, *J* = 12.9 Hz, 1H), 8.66 (s, 1H), 14.88 (s, 1H). ^13^C NMR (CDCl_3_, 75 MHz): δ (ppm) 8.4 (2 × C), 25.4, 28.3, 28.9, 29.5 (3 × C), 29.6, 32.9, 33.4, 34.3, 35.5, 41.2, 45.5, 49.5 (d, ^4^*J*_*C–F*_ = 2.3 Hz), 50.2 (d, ^4^*J*_*C–F*_ = 5.3 Hz), 105.2 (d, ^3^*J*_*C–F*_ = 3.0 Hz), 108.1, 112.4 (d, ^2^*J*_*C–F*_ = 23.3 Hz), 120.0 (d, ^3^*J*_*C–F*_ = 8.3 Hz), 139.1, 145.5 (d, ^2^*J*_*C–F*_ = 10.5 Hz), 147.6, 153.7 (d, ^1^*J*_*C–F*_ = 249.8 Hz), 166.9, 172.0, 177.0 (d, ^4^*J*_*C–F*_ = 2.3 Hz). HRMS (ESI) *m*/*z*: 578.2051 (calcd for C_28_H_38_BrFN_3_O_4_ [M + H]^+^, 578.2030).

#### 4.2.14 7-[4-(3,3,3-Trifluoropropionyl)-piperazin-1-yl]-1-cyclopropyl-6-fluoro-4-oxo-1,4-dihydro-quinoline-3-carboxylic Acid (**14**)

Pale beige solid, 0.32 g (24%). mp 292.8–293.9 °C. ^1^H NMR (CDCl_3_/CD_3_OD, 9:1 mixture, 500 MHz): δ (ppm) 1.23 (br s, 2H), 1.43 (br s, 2H), 3.35–3.38 (m, 4H), 3.40–3.41 (m, 2H), 3.61 (br s, 1H), 3.74 (br s, 2H), 3.91 (br s, 2H), 7.44 (br s, 1H), 8.03 (d, *J* = 13.0 Hz, 1H), 8.80 (s, 1H). ^13^C NMR (CDCl_3_/CD_3_OD, 9:1 mixture, 125 MHz): δ (ppm) 8.0 (2 × C), 35.3, 37.8 (q, ^2^*J*_*C–F*_ = 28.8 Hz, CF_3_), 41.5, 46.1, 49.0, 49.8 (d, ^4^*J*_*C–F*_ = 5.0 Hz), 105.3, 105.3, 112.5 (d, ^2^*J*_*C–F*_ = 23.8 Hz), 120.5 (d, ^3^*J*_*C–F*_ = 8.8 Hz), 123.9 (q, ^1^*J*_*C–F*_ = 275 Hz, CF_3_), 138.9, 144.9 (d, ^2^*J*_*C–F*_ = 3.8 Hz), 147.8, 153.5 (d, ^1^*J*_*C–F*_ = 250.0 Hz), 162.2 (q, ^3^*J*_*C–F*_ = 2.5 Hz, CF_3_), 167.7, 176.9 (d, ^4^*J*_*C–F*_ = 2.5 Hz). HRMS (ESI) *m*/*z*: 464.1238 (calcd for C_20_H_19_F_4_N_3_O_4_Na [M + Na]^+^, 464.1209).

#### 4.2.15 7-[4-(4,4,4-Trifluorobutyryl)-piperazin-1-yl]-1-cyclopropyl-6-fluoro-4-oxo-1,4-dihydro-quinoline-3-carboxylic Acid (**15**)

Pale beige solid, 0.51 g (37%). mp 291.3–292.8 °C. ^1^H NMR (CDCl_3_/CD_3_OD, 9:1 mixture, 500 MHz): δ (ppm) 1.21–1.25 (m, 2H), 1.41–1.45 (m, 2H), 2.50–2.60 (m, 2H), 2.66–2.69 (m, 2H), 3.34 (t, *J* = 5.0 Hz, 2H), 3.39–3.43 (m, 2H), 3.59 (s, 1H), 3.76 (d, *J* = 5.0 Hz, 2H), 3.89 (d, *J* = 5.0 Hz, 2H), 7.40 (d, *J* = 7.0 Hz, 1H), 8.01 (d, *J* = 13.0 Hz, 1H), 8.76 (s, 1H). ^13^C NMR (CDCl_3_/CD_3_OD, 9:1 mixture, 125 MHz): δ (ppm) 8.1 (2 × C), 25.7 (q, ^3^*J*_*C–F*_ = 2.5 Hz, CF_3_), 29.3 (q, ^2^*J*_*C–F*_ = 28.8 Hz, CF_3_), 35.4, 41.4, 45.1, 49.2 (d, ^4^*J*_*C–F*_ = 3.8 Hz), 49.8 (d, ^4^*J*_*C–F*_ = 5.0 Hz), 105.2 (d, ^3^*J*_*C–F*_ = 3.8 Hz), 107.7, 112.5 (d, ^2^*J*_*C–F*_ = 22.5 Hz), 120.2 (d, ^3^*J*_*C–F*_ = 7.5 Hz), 126.8 (q, ^1^*J*_*C–F*_ = 275 Hz, CF_3_), 138.9, 145.2 (d, ^2^*J*_*C–F*_ = 11.3 Hz), 147.7, 153.5 (d, ^1^*J*_*C–F*_ = 250.0 Hz), 167.2, 168.6, 176.9 (d, ^4^*J*_*C–F*_ = 2.5 Hz). HRMS (ESI) *m*/*z*: 478.1348 (calcd for C_21_H_21_F_4_N_3_O_4_Na [M + Na]^+^, 478.1366).

#### 4.2.16 7-[4-(5,5,5-Trifluoropentanoyl)-piperazin-1-yl]-1-cyclopropyl-6-fluoro-4-oxo-1,4-dihydro-quinoline-3-carboxylic Acid (**16**)

Pale beige solid, 0.47 g (33%). mp 221.1–222.3 °C. ^1^H NMR (CDCl_3_/CD_3_OD, 9:1 mixture, 500 MHz): δ (ppm) 1.23 (br s, 2H), 1.43 (br s, 2H), 1.93–1.99 (m, 2H), 2.18–2.28 (m, 2H), 2.51 (t, *J* = 7.0 Hz, 2H), 3.36 (br s, 2H), 3.40 (br s, 2H), 3.60 (br s, 1H), 3.74 (br s, 2H), 3.87 (br s, 2H), 7.44 (br s, 1H), 7.95 (d, *J* = 13.0 Hz, 1H), 8.72 (s, 1H). ^13^C NMR (CDCl_3_/CD_3_OD, 9:1 mixture, 125 MHz): δ (ppm) 8.1 (2 × C), 17.4 (q, ^3^*J*_*C–F*_ = 3.8 Hz, CF_3_), 31.3, 32.8 (q, ^2^*J*_*C–F*_ = 28.8 Hz, CF_3_), 35.4, 41.2, 45.1, 49.2, 49.8 (d, ^4^*J*_*C–F*_ = 6.3 Hz), 105.1 (d, ^3^*J*_*C–F*_ = 3.8 Hz), 107.6, 112.2 (d, ^2^*J*_*C–F*_ = 22.5 Hz), 120.0 (d, ^3^*J*_*C–F*_ = 7.5 Hz), 127.0 (q, ^1^*J*_*C–F*_ = 275 Hz, CF_3_), 138.9, 145.2 (d, ^2^*J*_*C–F*_ = 10.0 Hz), 147.6, 153.5 (d, ^1^*J*_*C–F*_ = 250.0 Hz), 167.1, 170.4, 176.8 (d, ^4^*J*_*C–F*_ = 2.5 Hz). HRMS (ESI) *m*/*z*: 492.1543 (calcd for C_22_H_23_F_4_N_3_O_4_Na [M + Na]^+^, 492.1522).

#### 4.2.17 CP–Acetyl–CP Dimer (**17**)

Pale beige solid, 0.19 g (9%). mp 231.3–232.6 °C. ^1^H NMR (DMSO-*d*_6_, 500 MHz): δ (ppm) 1.09–1.12 (m, 2H), 1.15–1.19 (m, 2H), 1.23–1.27 (m, 2H), 1.30–1.32 (m, 2H), 3.24 (br s, 2H), 3.29 (br s, 2H), 3.40 (br s, 2H), 3.49 (br s, 2H), 3.59–3.64 (m, 1H), 3.70 (br s, 5H), 3.82 (br s, 3H), 4.46 (s, 2H), 7.46 (d, *J* = 7.5 Hz, 1H), 7.54 (d, *J* = 7.0 Hz, 1H), 7.76 (d, *J* = 13.5 Hz, 1H), 7.84 (d, *J* = 13.0 Hz, 1H), 8.10 (s, 1H), 8.62 (s, 1H), 15.15 (s, 1H). ^13^C NMR (DMSO-*d*_6_, 125 MHz): δ (ppm) 7.6 (2 × C), 7.6 (2 × C), 34.3, 35.9, 41.3, 41.4, 41.9, 45.1, 46.4, 49.3 (d, ^4^*J*_*C–F*_ = 2.5 Hz, 2 × C), 49.7 (d, ^4^*J*_*C–F*_ = 2.5 Hz), 49.9 (d, ^4^*J*_*C–F*_ = 2.5 Hz), 106.3 (d, ^3^*J*_*C–F*_ = 1.3 Hz), 106.4, 106.7 (d, ^3^*J*_*C–F*_ = 2.6 Hz), 110.9 (d, ^2^*J*_*C–F*_ = 22.5 Hz), 111.2 (d, ^2^*J*_*C–F*_ = 22.5 Hz), 116.7, 118.7 (d, ^3^*J*_*C–F*_ = 6.3 Hz), 120.8 (d, ^3^*J*_*C–F*_ = 6.3 Hz), 138.4, 139.1, 143.5 (d, ^2^*J*_*C–F*_ = 10.0 Hz), 144.1, 144.9 (d, ^2^*J*_*C–F*_ = 10.0 Hz), 147.9, 152.4 (d, ^1^*J*_*C–F*_ = 246.3 Hz), 152.9 (d, ^1^*J*_*C–F*_ = 247.5 Hz), 164.8, 165.1, 165.9, 171.3 (d, ^4^*J*_*C–F*_ = 2.5 Hz), 176.2. HRMS (ESI) *m*/*z*: 721.2781 (calcd for C_36_H_39_F_2_N_6_O_8_ [M + H_2_O + H]^+^, 721.2797).

#### 4.2.18 CP–Butyryl–CP Dimer (**18**)

White solid, 0.18 g (8%). mp 174.5–176.6 °C. ^1^H NMR (DMSO-*d*_6_, 500 MHz): δ (ppm) 1.08–1.12 (m, 2H), 1.16–1.19 (m, 2H), 1.23–1.27 (m, 2H), 1.30–1.34 (m, 2H), 1.96–2.01 (m, 2H), 2.53 (t, *J* = 7.0 Hz, 2H), 3.21 (br s, 2H), 3.26 (br s, 2H), 3.35–3.40 (m, 5H), 3.48 (br s, 2H), 3.58–3.63 (m, 1H), 3.67–3.70 (m, 5H), 3.79–3.84 (m, 3H), 7.46 (d, *J* = 7.5 Hz, 1H), 7.55 (d, *J* = 7.5 Hz, 1H), 7.76 (d, *J* = 13.5 Hz, 1H), 7.84 (d, *J* = 13.0 Hz, 1H), 8.10 (s, 1H), 8.62 (s, 1H), 15.15 (s, 1H). ^13^C NMR (DMSO-*d*_6_, 125 MHz): δ (ppm) 7.6 (2 × C), 7.6 (2 × C), 27.9, 29.3, 34.3, 35.9, 40.8, 41.3, 44.6, 45.1, 46.4, 49.3 (d, ^4^*J*_*C–F*_ = 2.5 Hz), 49.5 (d, ^4^*J*_*C–F*_ = 2.5 Hz), 49.9 (d, ^4^*J*_*C–F*_ = 2.5 Hz, 2 × C), 106.2 (d, ^3^*J*_*C–F*_ = 2.5 Hz), 106.4 (d, ^3^*J*_*C–F*_ = 3.8 Hz), 106.7, 110.9 (d, ^2^*J*_*C–F*_ = 22.5 Hz), 111.2 (d, ^2^*J*_*C–F*_ = 22.5 Hz), 116.7, 118.7 (d, ^3^*J*_*C–F*_ = 7.5 Hz), 120.7 (d, ^3^*J*_*C–F*_ = 6.3 Hz), 138.4, 139.1, 143.6 (d, ^2^*J*_*C–F*_ = 10.0 Hz), 144.1, 144.9 (d, ^2^*J*_*C–F*_ = 10.0 Hz), 147.9, 152.4 (d, ^1^*J*_*C–F*_ = 245.0 Hz), 152.9 (d, ^1^*J*_*C–F*_ = 247.5 Hz), 165.1, 165.9, 169.8, 171.3 (d, ^4^*J*_*C–F*_ = 2.5 Hz), 176.2 (d, ^4^*J*_*C–F*_ = 2.5 Hz). HRMS (ESI) *m*/*z*: 771.2947 (calcd for C_38_H_42_F_2_N_6_O_8_Na [M + H_2_O + Na]^+^, 771.2930).

#### 4.2.19 CP–Pentanoyl–CP Dimer (**19**)

White solid, 0.11 g (5%). mp 170.3–171.9 °C. ^1^H NMR (CDCl_3_, 500 MHz): δ (ppm) 1.15–1.22 (m, 4H), 1.31–1.36 (m, 2H), 1.40–1.43 (m, 2H), 1.81–1.91 (m, 4H), 2.44 (t, *J* = 7.0 Hz, 2H), 3.25 (t, *J* = 5.0 Hz, 2H), 3.30 (t, *J* = 5.0 Hz, 2H), 3.35–3.40 (m, 1H), 3.43–3.52 (m, 4H), 3.56–3.63 (m, 5H), 3.72 (t, *J* = 5.0 Hz, 2H), 3.87 (t, *J* = 5.0 Hz, 2H), 3.99 (br s, 2H), 7.32 (d, *J* = 7.0 Hz, 1H), 7.40 (d, *J* = 6.5 Hz, 1H), 7.94–7.99 (m, 2H), 8.14 (s, 1H), 8.72 (s, 1H), 14.99 (s, 1H). ^13^C NMR (CDCl3, 125 MHz): δ (ppm) 8.1 (2 × C), 8.3 (2 × C), 22.4, 32.0, 32.2, 34.4, 35.4, 41.3, 42.3, 44.7, 45.4, 47.3, 49.6 (2 × C), 50.3 (d, ^4^*J*_*C–F*_ = 3.8 Hz), 50.5 (d, ^4^*J*_*C–F*_ = 3.8 Hz), 104.9, 105.2 (d, ^3^*J*_*C–F*_ = 2.5 Hz), 108.0, 112.3 (d, ^2^*J*_*C–F*_ = 22.5 Hz), 112.8 (d, ^2^*J*_*C–F*_ = 22.5 Hz), 117.0, 119.9 (d, ^3^*J*_*C–F*_ = 7.5 Hz), 121.9 (d, ^3^*J*_*C–F*_ = 6.3 Hz), 138.3, 139.0, 144.2 (d, ^2^*J*_*C–F*_ = 11.3 Hz), 145.3, 145.7 (d, ^2^*J*_*C–F*_ = 11.3 Hz), 147.4, 153.2 (d, ^1^*J*_*C–F*_ = 247.5 Hz), 153.6 (d, ^1^*J*_*C–F*_ = 250.0 Hz), 165.9, 166.9, 170.9, 172.2 (d, ^4^*J*_*C–F*_ = 1.3 Hz), 176.9 (d, ^4^*J*_*C–F*_ = 2.5 Hz). HRMS (ESI) *m*/*z*: 785.3067 (calcd for C_39_H_44_F_2_N_6_O_8_Na [M + H_2_O + Na]^+^, 785.3086).

#### 4.2.20 CP–Hexanoyl–CP Dimer (**20**)

Pale beige solid, 0.21 g (9%). mp 161.2–162.9 °C. ^1^H NMR (CDCl_3_, 500 MHz): δ (ppm) 1.15–1.22 (m, 4H), 1.32–1.36 (m, 2H), 1.40–1.44 (m, 2H), 1.50–1.56 (m, 2H), 1.68–1.74 (m, 2H), 1.80–1.86 (m, 2H), 2.42 (t, *J* = 7.5 Hz, 2H), 3.25 (t, *J* = 5.0 Hz, 2H), 3.31 (t, *J* = 5.0 Hz, 2H), 3.45–3.51 (m, 5H), 3.56 (t, *J* = 6.5 Hz, 2H), 3.59–3.64 (m, 3H), 3.73 (t, *J* = 5.0 Hz, 2H), 3.86 (t, *J* = 5.0 Hz, 2H), 3.99 (br s, 2H), 7.32 (d, *J* = 7.0 Hz, 1H), 7.39 (d, *J* = 7.0 Hz, 1H), 7.86 (d, *J* = 13.0 Hz, 1H), 7.92 (d, *J* = 13.0 Hz, 1H), 8.12 (s, 1H), 8.66 (s, 1H), 14.99 (s, 1H). ^13^C NMR (CDCl3, 125 MHz): δ (ppm) 8.0 (2 × C), 8.1 (2 × C), 24.3, 26.5, 32.2, 32.8, 34.3, 35.3, 41.1, 42.1, 44.8, 45.3, 47.1, 49.4 (d, ^4^*J*_*C–F*_ = 2.5 Hz), 49.5, 50.1 (d, ^4^*J*_*C–F*_ = 3.8 Hz), 50.3 (d, ^4^*J*_*C–F*_ = 3.8 Hz), 104.9 (d, ^3^*J*_*C–F*_ = 2.5 Hz), 105.1 (d, ^3^*J*_*C–F*_ = 3.8 Hz), 107.6, 111.9 (d, ^2^*J*_*C–F*_ = 22.5 Hz), 112.6 (d, ^2^*J*_*C–F*_ = 22.5 Hz), 116.9, 119.6 (d, ^3^*J*_*C–F*_ = 7.5 Hz), 121.7 (d, ^3^*J*_*C–F*_ = 7.5 Hz), 138.2, 138.9, 144.1 (d, ^2^*J*_*C–F*_ = 10.0 Hz), 145.1, 145.6 (d, ^2^*J*_*C–F*_ = 10.0 Hz), 147.2, 153.0 (d, ^1^*J*_*C–F*_ = 246.3 Hz), 153.4 (d, ^1^*J*_*C–F*_ = 250.0 Hz), 165.8, 166.7, 171.2, 172.1 (d, ^4^*J*_*C–F*_ = 1.3 Hz), 176.7 (d, ^4^*J*_*C–F*_ = 2.5 Hz). HRMS (ESI) *m*/*z*: 799.3218 (calcd for C_40_H_46_F_2_N_6_O_8_Na [M + H_2_O + Na]^+^, 799.3243).

#### 4.2.21 CP–Undecanoyl–CP Dimer (**21**)

Pale beige solid, 75 mg (3%). mp 141.6–142.9 °C. ^1^H NMR (CDCl_3_, 300 MHz): δ (ppm) 1.14–1.23 (m, 4H), 1.30–1.46 (m, 16H), 1.62–1.71 (m, 2H), 1.79–1.90 (m, 2H), 2.40 (t, *J* = 8.1 Hz, 2H), 3.25–3.31 (m, 4H), 3.38–3.53 (m, 7H), 3.60–3.66 (m, 3H), 3.71 (br s, 2H), 3.86 (br s, 2H), 3.99 (br s, 2H), 7.31 (d, *J* = 8.4 Hz, 1H), 7.40 (d, *J* = 7.2 Hz, 1H), 7.92 (d, *J* = 9.0 Hz, 1H), 7.96 (d, *J* = 9.0 Hz, 1H), 8.13 (s, 1H), 8.71 (s, 1H), 15.01 (s, 1H). ^13^C NMR (CDCl3, 75 MHz): δ (ppm) 8.3, 8.4, 8.7 (2 × C), 25.4, 28.2, 28.8, 29.4, 29.5, 29.6, 32.9, 33.4, 34.2, 34.6, 35.6, 41.3, 42.4, 45.6, 46.2, 47.4, 49.8 (2 × C), 50.4, 50.6 (d, ^4^*J*_*C–F*_ = 3.0 Hz), 105.1, 105.4, 108.0, 112.3 (d, ^2^*J*_*C–F*_ = 22.5 Hz), 112.9 (d, ^2^*J*_*C–F*_ = 22.5 Hz), 117.1, 120.0 (d, ^3^*J*_*C–F*_ = 7.5 Hz), 122.0 (d, ^3^*J*_*C–F*_ = 7.5 Hz), 138.5, 139.1, 144.4 (d, ^2^*J*_*C–F*_ = 10.5 Hz), 145.4, 145.8 (d, ^2^*J*_*C–F*_ = 10.5 Hz), 147.5, 152.8 (d, ^1^*J*_*C–F*_ = 247.5 Hz), 153.2 (d, ^1^*J*_*C–F*_ = 249.8 Hz), 165.1, 167.0, 171.9, 172.4, 177.1 (d, ^4^*J*_*C–F*_ = 2.3 Hz). HRMS (ESI) *m*/*z*: 869.4058 (calcd for C_45_H_56_F_2_N_6_O_8_Na [M + H_2_O + Na]^+^, 869.4025).

### 4.3 Biological Studies

#### 4.3.1 In Vitro Antibacterial Studies

To characterize the antibacterial activity of fluoroquinolone conjugates, reference bacterial strains from international microbe collections, American Type Culture Collection (ATTC) and National Collection of Type Culture (NCTC), as well as a panel of clinical rods, were studied. The first set contains two Gram-negative organisms, *E. coli* ATCC 25922 and *P. aeruginosa* ATCC 15442, and a series of six Gram-positive strains: *S. aureus*: NCTC 4163; ATCC: 29213, 25923, and 6538; and *S. epidermidis* ATCC: 12228 and 35984. The group of clinical strains consisted of isolates of *S. aureus* (180, 5595, T5595, and T5591), *S. epidermidis* (4341, 5253, and KR4243/1), *S. pasteuri* (4358), *P. aeruginosa* (37 and 659), *E. coli* (951, 520, and 600), *E. cloacae* (8), and *K. pneumoniae* (28 and 510). Antibiotic susceptibility testing including the resistance phenotypes of hospital strains were determined using VITEK 2 Compact and VITEK 2 AES.

The MIC was determined by the twofold microdilution method according to the CLSI reference procedure with some modifications.^51^ The bacteria were cultured in brain heart infusion agar (BHI) and incubated at 37 °C for 24–48 h. Bacterial inoculum were prepared in a sterile saline solution and diluted in MH II liquid medium to a final concentration of 10^6^ colony-forming units per mL (cfu/mL). The reference CP was tested at the range of 0.03–32 μg/mL, whereas the concentrations of conjugates varied from 0.025 to 25.6 μg/mL. The final concentration of DMSO in working solutions was less than 1%. Bacteria were grown overnight in the presence of different concentrations of the tested compounds. After a 18 h period of incubation, the lowest concentration of drugs that inhibited the visible growth of bacteria was considered as the MIC value. Tests were repeated independently three times.

The VITEK 2 Compact (BioMérieux) automated system for the antimicrobial susceptibility testing of microorganisms was used in accordance with the manufacturer’s directions.

#### 4.3.2 Bacterial Growth Curve Assay

The growth rate of *P. aeruginosa* ATCC 15442, *E. coli* ATCC 25922, and *S. aureus* ATCC 6538 strains was observed by inoculating the microtiter plates with BHI broth, containing 5 × 10^5^ colony-forming units (cfu) per mL of bacteria, loaded with varying concentrations (0.8–0.0016 μg/mL) of compounds **5**, **10**, **11**, and CP as the reference drug. The plates were incubated at 37 °C and rotated at 180 rpm. After inoculation, the optical density (OD) at 600 nm was monitored every 5 min interval for 18 h. Assays were repeated three times on different days. The results were expressed as the mean of three experiments.

#### 4.3.3 Biofilm Eradication Assay

Three bacterial strains *E. coli* ATCC 25922, *P. aeruginosa* ATCC 15442, and *S. aureus* ATCC 6538 were used to assign biofilm eradication properties of compounds **5**, **10**, **11**, and CP as the reference drug. To quantify the biomass of the bacteria biofilm, the crystal violet method was used with some modifications.^52^ The bacteria were incubated to the mid-log phase, diluted to 103 cfu/mL in brain–heart infusion medium, and seeded in a 96-well plate. Bacterial biofilms were formed by overnight incubation at 37 °C with rotation at 140 rpm. After biofilm formation, all wells were washed with PBS to remove nonadherent bacteria cells. Next, the biofilm was treated with compounds at concentrations ranging from 1/2 MIC to 8 MIC (Table S1). The biofilms with tested substances were incubated for 20 h at 37 °C with shaking at 140 rpm. The bacterial biofilm was stained with 0.1% (v/v) CV for 10 min. Afterward, the biofilm was rinsed with PBS and dried. To solubilize the adsorbed crystal violet, wells with stained biofilms were incubated in 30% acetic acid. To estimate the total biofilm biomass, optical density (OD) of the resulting solution was measured at 595 nm. Biofilm assays were repeated at least three times on different days, with two technical replicates assessed each time. A minimum eradication concentration (MBEC) was determined as the lowest concentration of compound, which could damage ≥50% of the formed biofilm structure.

#### 4.3.4 In Vitro Antimycobacterial Activity

The synthesized compounds **1–21** were tested in vitro for their tuberculostatic activity against typical strains [*M. tuberculosis* H_37_Rv strain (ATCC 25618), *M. tuberculosis* Spec. 210, and *M. tuberculosis* Spec. 192] using the MABA method (Microplate Alamar Blue Assay method).^53,54^ Investigations were performed by the twofold serial microdilution method (in 96-well microliter plates) using the Middlebrook 7H9 Broth medium (Beckton Dickinson) containing 10% of OADC (Beckton Dickinson). The inoculum was prepared from fresh LJ culture in the Middlebrook 7H9 Broth medium with OADC, adjusted to a no. 1 McFarland tube, and diluted 1:20. The stock solution of a tested agent was prepared in DMSO. Each stock solution of a tested compound was diluted in the Middlebrook 7H9 Broth medium with OADC by 4-fold the final highest concentration to be tested. Compounds were diluted serially in a sterile 96-well microtiter plates using 100 μL Middlebrook 7H9 Broth medium with OADC. The concentrations of tested agents ranged from 0.125 to 512 μg/mL. A growth control containing no antibiotic and a sterile control without inoculation were also prepared on each plate. The plates were incubated at 37 °C for a week. After the incubation period, 30 μL of Alamar blue solution was added to each well, and the plate was reincubated for 24 h. The growth was indicated by the color change from blue to pink. The lowest concentration of a compound that prevented the color change was considered as its MIC. CP, isoniazid (INH), rifampicin (RMP), streptomycin (SM), and ethambutol (EMB) were used as reference drugs.

#### 4.3.5 Inhibition of Bacterial DNA Topoisomerases—Enzymatic Assay

The enzymatic assay was carried using commercial topoisomerases type II as previously described by Alt et al.^55^ with some modifications.

Briefly, 1 U of the enzyme (gyrase or topo IV, Inspiralis. Norwich, UK) isolated from *S. aureus* converts 0.5 mg of relaxed pBR322 DNA to the supercoiled form (gyrase) or decatenates 200 ng of kinetoplast DNA (topo IV). The enzymatic assay was performed by incubation for 30 min at 37 °C in a total reaction volume of 30 μL and in the presence of different concentrations of the tested compounds. The reactions were terminated by adding an equal volume of STEB buffer (40% sucrose, 100 mM Tris–HCl pH 8, 10 mM EDTA, and 0.5 mg/mL bromophenol blue), followed by extraction with 1 volume of chloroform/isoamyl alcohol (24:1). The samples were vortexed and centrifuged, and 20 μL of the aqueous phase of each sample was loaded onto a 1% agarose gel and left for 30 min prior to electrophoresis to allow diffusion of salt. Electrophoresis was conducted in TAE buffer for 3 h at 50 V (gyrase) or for 1.5 h at 80 V (topo IV). Gels were stained with ethidium bromide and visualized under UV light in a transilluminator (ChemiDoc MP, Bio Rad). The IC_50_ values were defined as the concentration causing 50% inhibition of the supercoiling or the decatenation reaction, as can be seen with the drug-free controls and were determined by plotting the results obtained from the densitometric analyses of the gel images using Image Lab software (BioRad).

#### 4.3.6 Molecular Docking Studies

The 13 studied compounds and CP were docked to structures of DNA gyrase (PDB ID: 5BTC)^48^ and DNA topoisomerase IV (PDB ID: 3RAD)^49^ (see Table S2). The docking procedure is as follows. Ligand structures were generated using the Automated Topology Builder (ATB version 2.2),^56^ and topologies were created using the Avogadro^57^ program. Docking calculations and data analysis were performed using AutoDock4 (v. 4.2) and AutoDockTools4 programs, respectively.^58^ 1000 models were generated for each complex during the docking procedure. Preferred binding modes were selected based on structural clustering with an rmsd cut-off value of 3 Å. The central structure of the largest cluster was selected as the final ligand-docked structure for each complex.

#### 4.3.7 Cytotoxic Activity

##### 4.3.7.1 Cell Culture

Metastatic prostate cancer (PC3) and human immortal keratinocyte (HaCaT) cell lines were purchased from the American Type Culture Collection (ATCC, Rockville, MD, USA) and cultured in MEM (minimal essential medium, Thermo Sci, USA), RPMI 1640 (Roswell Park Memorial Institute, Biowest SAS, France), and DMEM (Dulbecco’s Modified Eagle’s Medium, Biowest SAS, France). Cells were seeded in 6 mL medium in a tissue culture flask (50 mL) in a 37 °C/5% CO_2_ humidified incubator. The medium was supplemented with 10% heat-inactivated fetal bovine serum (FBS), penicillin (100 U/mL), streptomycin (100 μg/mL) (Gibco BRL San Francisco, CA, USA), and HEPES (20 mM, Thermo Sci (Waltham, MA, USA). The cells were cultured until 80–90% confluency was reached and then were harvested by treatment with 0.25% trypsin-0.02% EDTA (Gibco BRL, San Francisco, CA, USA) and used for experiments.

##### 4.3.7.2 MTT Assay

CP conjugates, the parental drug and leading cytostatics—doxorubicin and cisplatin, were tested at various concentrations (ranged from 1 to 100 μM). They were added on 96-well plates (1 × 10^4^ cells per well) with seeded normal and cancer cells and incubated for 72 h. MTT analysis was performed according to a previous study.^59^ Untreated cells were used as controls.

Cell absorbance results were inserted into the formula for the relative MTT level (%). It allows to calculate the viability of cells under the influence of the tested compounds. Cell viability was expressed as the percentage of MTT reduction in cells treated with tested compounds compared to the control sample.

The relative MTT level was calculated using the formula [100%] = *A*/*B* × 100% where *A*—the test sample absorbance and *B*—the control sample absorbance. The IC_50_ values were estimated using CompuSyn version 1.0.

##### 4.3.7.3 Annexin V-FITC/PI Binding Assay

The PC3 and HaCaT cells were cultured and harvested under the conditions mentioned in the Cell Culture section and seeded in 12-well plates (1 × 10^5^ cells per well). After 24 h of preincubation, the cells were treated with the tested compounds at IC_50_ concentrations and incubated for 72 h. The apoptotic effect was performed using the annexin V-FITC/propidium iodide (PI) apoptosis assay kit (Becton Dickinson, Pharmingen) according to the manufacturer’s instructions and analyzed by flow cytometry (Becton Dickinson). The cells which were annexin V-FITC-positive and PI-negative were identified as early apoptotic and both annexin V-FITC- and PI-positive as late apoptotic or necrotic. The experiment was repeated three times.

##### 4.3.7.4 Interleukin-6 Assay

The level of interleukin-6 (IL-6) in PC3 and HaCaT cell lines was measured by commercial human IL-6 ELISA kits Diaclon SAS (Besancon Cedex, France). The cells were treated with IC_50_ concentrations of the tested compounds for 72 h. The untreated cells were used as the control. The IL-6 level in a cell culture supernatant was measured using an enzyme-linked immunosorbent assay in accordance with the manufacturer’s instruction. The experiment was repeated three times.

##### 4.3.7.5 ROS Detection—DCFH-DA and DHR-123 Assay

ROS generation was assessed by the spectrofluorometric method using 2′,7′-dichlorodihydrofluorescein diacetate (DCFH-DA) or dihydrorhodamine 123 (DHR-123). The method is based on the ROS-dependent oxidation of the compounds to fluorescent dichlorofluorescein (DCF) or rhodamine-123, respectively. PC3 and HaCaT were seeded on to 96-well plates (5 × 10^4^ cells per well) and allowed to adhere for 24 h. Then, the cells were rinsed with PBS and incubated with DCFH-DA (5 μM) or DHR-123 (1 μM) for 30 min at 37 °C in the dark. Thereafter, the cells were rinsed with PBS and treated for 2, 12, and 72 h at 37 °C with red phenol-free culture medium containing tested compounds at their IC_50_ concentrations to observe the level of ROS. A sample with H_2_O_2_ (1.5 mM) was a positive control, and a sample without any reagent was a negative control. Maximum excitation and emission spectra for DCF were 492 and 527 nm, and those for rhodamine-123 were 500 and 536 nm, respectively. The generation of H_2_O_2_ was measured by Microplate Spectrofluorometer BioTek Synergy (BioTek Instruments, USA) and expressed as fluorescence intensity (FI). Values from three experiments performed in triplicate were analyzed.

##### 4.3.7.6 Statistical Analysis

The statistical calculation was performed using the Statistica 13.1 (StatSoft, Inc, USA) program. The quantitative comparisons were made using Student’s *t*-test. The IC_50_ values were estimated by CompuSyn version 1.0. The results of all presented experiments were expressed as the mean ± SD and considered statistically significant at *p* < 0.05.

## Supporting Information Available

Growth curve analysis of *S. aureus* ATCC 6538 at an absorbance of 600 nm (OD_600_) with or without different concentrations of compounds **5**, **10**, and **11** and **CP**; MIC (μg/mL) values of compounds **5**, **10**, **11**, and **CP** used in biofilm eradication assay; DNA gyrase (PDB ID: 5BTC) and DNA topoisomerase IV (PDB ID: 3RAD) binding data based on docking results for compounds **1–13** and **CP**; trypan blue assay; effect of compounds **3**, **15**, and **21** on live cell number and viability in PC3 and HaCaT cells; effect of compounds **3**, **15**, and **21** on early and late apoptosis or necrosis in PC3 and HaCaT cells; effect of CP conjugates **3**, **15**, and **21** on ROS production in PC3 and HaCaT cells; ^1^H and ^13^C NMR spectra of the synthesized **CP** derivatives **1–21** (PDF)
