# Supplementary material for: N-Acylated Ciprofloxacin Derivatives: Synthesis and In Vitro Biological Evaluation as Antibacterial and Anticancer Agents
Source: ACS Omega. 2023 May 18;8(21):18663–84. doi: 10.1021/acsomega.3c00554 (PMC10233829; doi:10.1021/acsomega.3c00554)

Supporting Information

N-acylated ciprofloxacin derivatives: synthesis and *in vitro* biological evaluation as antibacterial and anticancer agents

Marta Struga[1], Piotr Roszkowski[2,*], Anna Bielenica[1,*], Dagmara Otto-Ślusarczyk[1], Karolina Stępień[3], Joanna Stefańska[3], Anna Zabost[4], Ewa Augustynowicz-Kopeć[4], Michał Koliński[5], Sebastian Kmiecik[6], Alina Myslovska[2], Małgorzata Wrzosek[7]

[1] Chair and Department of Biochemistry, Medical University of Warsaw, ul. Banacha 1, 02-097 Warsaw, Poland

[2] Faculty of Chemistry, University of Warsaw, Pasteura 1, 02-093 Warsaw, Poland

[3] Department of Pharmaceutical Microbiology, Centre for Preclinical Research, Medical University of Warsaw, 02-097 Warsaw, Poland

[4] Department of Microbiology, National Tuberculosis and Lung Diseases Research Institute, 01-138 Warsaw, Poland

[5] Bioinformatics Laboratory, Mossakowski Medical Research Institute, Polish Academy of Sciences, 5 Pawinskiego St., 02-106 Warsaw, Poland

[6] Biological and chemical Research Centre, Faculty of Chemistry, University of Warsaw, 02-089 Warsaw, Poland

[7] Department of Biochemistry and Pharmacogenomics, Faculty of Pharmacy, Medical University of Warsaw, 02-097 Warsaw, Poland

[*]Corresponding authors: Anna Bielenica; abielenica@wum.edu.pl (biological activity), Piotr Roszkowski; roszkowski@chem.uw.edu.pl (chemistry)

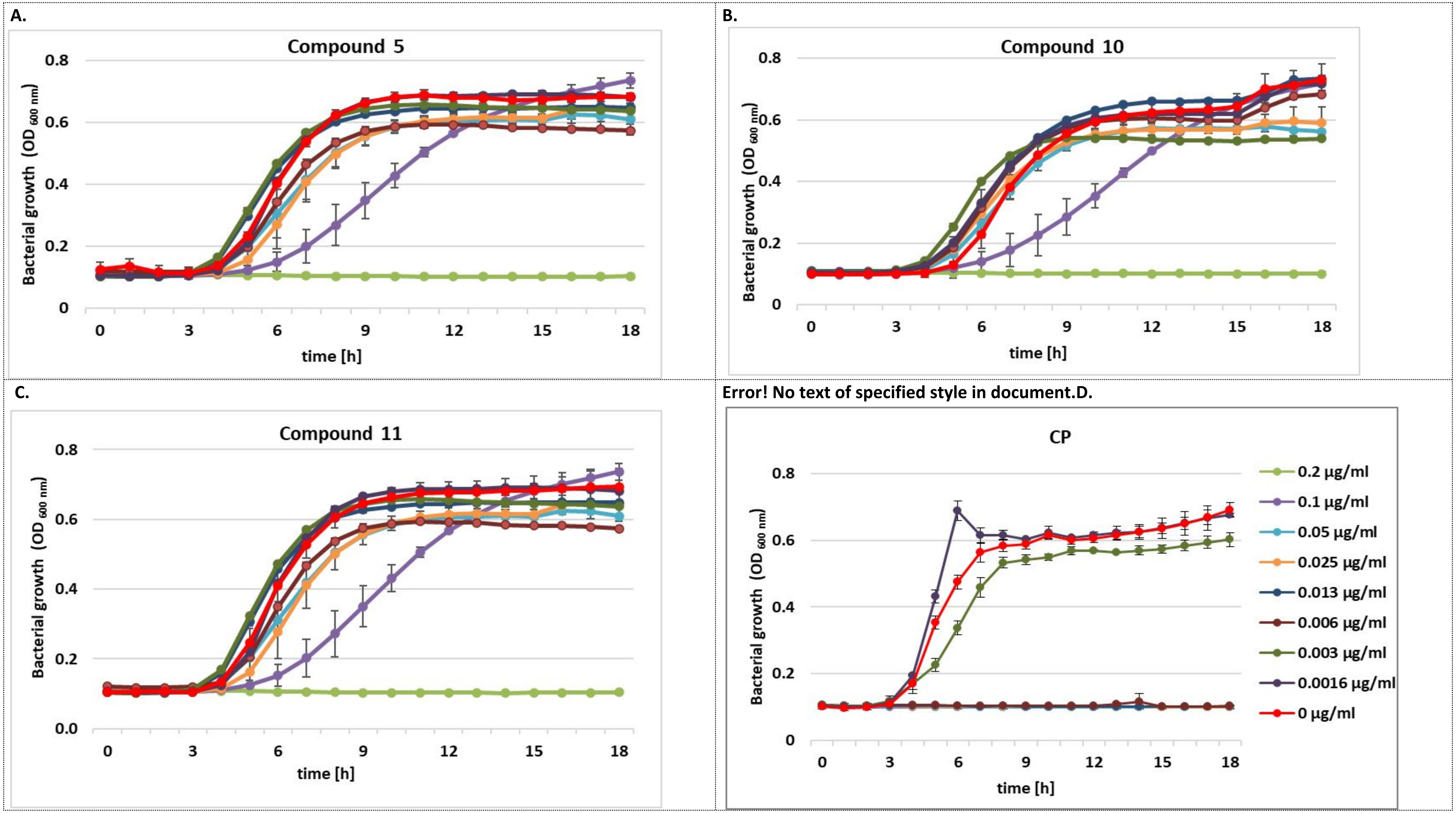

Figure S1. Growth curve analysis of *S. aureus* ATCC 6538 at absorbance of 600 nm (OD600) with or without different concentrations of **A.** compound **5**, **B.** compound **10**, **C.** compound **11**, **D.** CP for 18h. The growth curve data were plotted as average values with standard deviations of n = 3.

Table S1. MIC [ug/ml] values of compounds **5**, **10**, **11** and ciprofloxacin, used in biofilm eradication assay.

**Bacteria strain**	4 MIC **5**	4 MIC **10**	4 MIC **11**	4 MIC **CP**	2 MIC **5**	2 MIC **10**	2 MIC **11**	2 MIC **CP**	MIC **5**	MIC **10**	MIC **11**	MIC **CP**	1/2 MIC **5**	1/2 MIC **10**	1/2 MIC **11**	1/2 MIC **CP**
***E. coli* 25988**	0.1	0.1	0.1	0.1	0.05	0.05	0.05	0.03	0.025	0.025	0.025	0.015	0.013	0.0125	0.0125	0.0075
***S. aureus* 6538**	0.8	0.8	0.4	0.5	0.4	0.4	0.2	0.25	0.200	0.200	0.100	0.125	0.1	0.1	0.05	0.0625
***P. aeruginosa* 15442**	0.8	0.4	0.4	0.2	0.4	0.2	0.2	0.12	0.200	0.100	0.100	0.060	0.1	0.05	0.05	0.03

(Column headers: **Concentration [μg/ml]**; **Compound**)

Table S2. DNA gyrase (PDB ID: 5BTC [1]) and DNA topoisomerase IV (PDB ID: 3RAD [2]) binding data based on docking results for compounds **1-13** and **CP**.

**Compound**	**DNA gyrase**		**DNA topoisomerase IV**	
	**CS**	**BE (kcal/mol)**	**CS**	**BE (kcal/mol)**
**1**	433	-7.86	660	-6.7200
**2**	360	-8.65	597	-7.3400
**3**	454	-7.64	714	-6.7100
**4**	409	-8.46	701	-6.8700
**5**	343	-9.12	662	-7.9100
**6**	340	-9.93	754	-8.2300
**7**	350	-10.79	647	-9.0800
**8**	408	-7.99	645	-7.4900
**9**	399	-8.48	672	-7.6300
**10**	418	-9.49	833	-8.5300
**11**	381	-10.29	830	-9.2100
**12**	392	-10.74	854	-10.0900
**13**	171	-12.46	219	-12.2000
**CP**	753	-7.26	698	-6.1700

CS = number of members of the largest cluster calculated for 1000 docking runs using RMSD cutoff tolerance = 3 Å.
BE = binding free energy values estimated using AutoDock4 energy function for the representative ligand structure of the largest cluster.

Table S3. Trypan blue assay. The effect of compounds **3**, **15** and **21** on live cell number and viability in PC3 and HaCaT cells. Cells were incubated for 72 h with tested compounds used in their IC_{50} concentrations, then cells were harvested, stained with trypan blue, and analyzed using cell counter. Data are expressed as the mean ± SD. „-“ control without compound, [a]Human metastatic prostate cancer (PC3), [b]Human immortal keratinocyte cell line from adult human skin (HaCaT).

		Compound	Cell number x 10^6	Cell number (% of control)	Viability (%)
**Cancer cell line**	**PC3**[a]	**-**	2.4 ± 0.90	100	98 ± 1.01
		**3**	0.04 ± 0.01	1.97	52 ± 2.40
		**15**	0.3 ± 0.08	12.04	82 ± 2.64
		**21**	0.2 ± 0.03	6.22	78 ± 1.50
**Normal cell line**	**HaCaT**[b]	**-**	1.5 ± 0.45	100	97 ± 2.01
		**3**	0.2 ± 0.01	12.15	75 ± 2.23
		**15**	0.8 ± 0.10	48.38	90 ± 3.04
		**21**	0.3 ± 0.01	19.35	94 ± 4.01

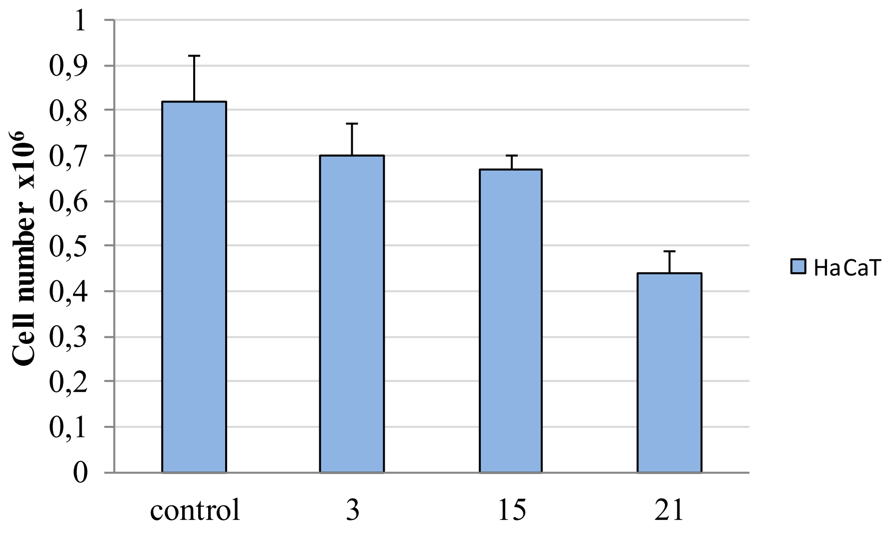

Figure S2. Trypan blue assay. The effect of compounds **3**, **15** and **21** on live cell number and viability in HaCaT cells. Compounds were used in their IC_{50} for PC3 cells, 2.02 μM, 15.7 μM and 4.8 μM, respectively.

Table S4. The effect of compounds **3**, **15** and **21** on early and late apoptosis or necrosis in PC3 and HaCaT cells detected with Annexin V-FITC/PI by flow cytometry. Cells which were Annexin V:FITC positive and PI negative were identified as early apoptosis, and Annexin V:FITC and PI positive as late apoptosis or necrosis. Data are expressed as the mean ± SD from 3 independent experiments.

**PC3 cells**				
Compound	Live cells	Early apoptosis	Late apoptosis	Dead cells
**control**	92.03 ± 1.04	7.61 ± 1.05	0.21 ± 0.06	0.15 ± 0.04
**3**	20.27 ± 0.45	1.51 ± 0.04	12.12 ± 2.18	66.10 ± 6.31
**15**	61.21 ± 2.06	1.15 ± 0.10	16.49 ± 2.65	21.15 ± 3.29
**21**	2.59 ± 0.20	28.22 ± 0.98	16.28 ± 2.80	52.91 ± 3.99

**HaCaT cells**				
Compound	Live cells	Early apoptosis	Late apoptosis	Dead cells
**control**	95.78 ± 0.77	2.30 ± 0.65	0.97 ± 0.22	0.95 ± 0.17
**3**	42.70 ± 2.81	10.07 ± 1.22	37.24 ± 2.27	9.99 ± 1.17
**15**	87.32 ± 2.20	11.63 ± 2.93	0.77 ± 0.05	0.3 ± 0.01
**21**	54.53 ± 3.34	10.52 ± 1.07	20.71 ± 4.34	14.24 ± 3.78

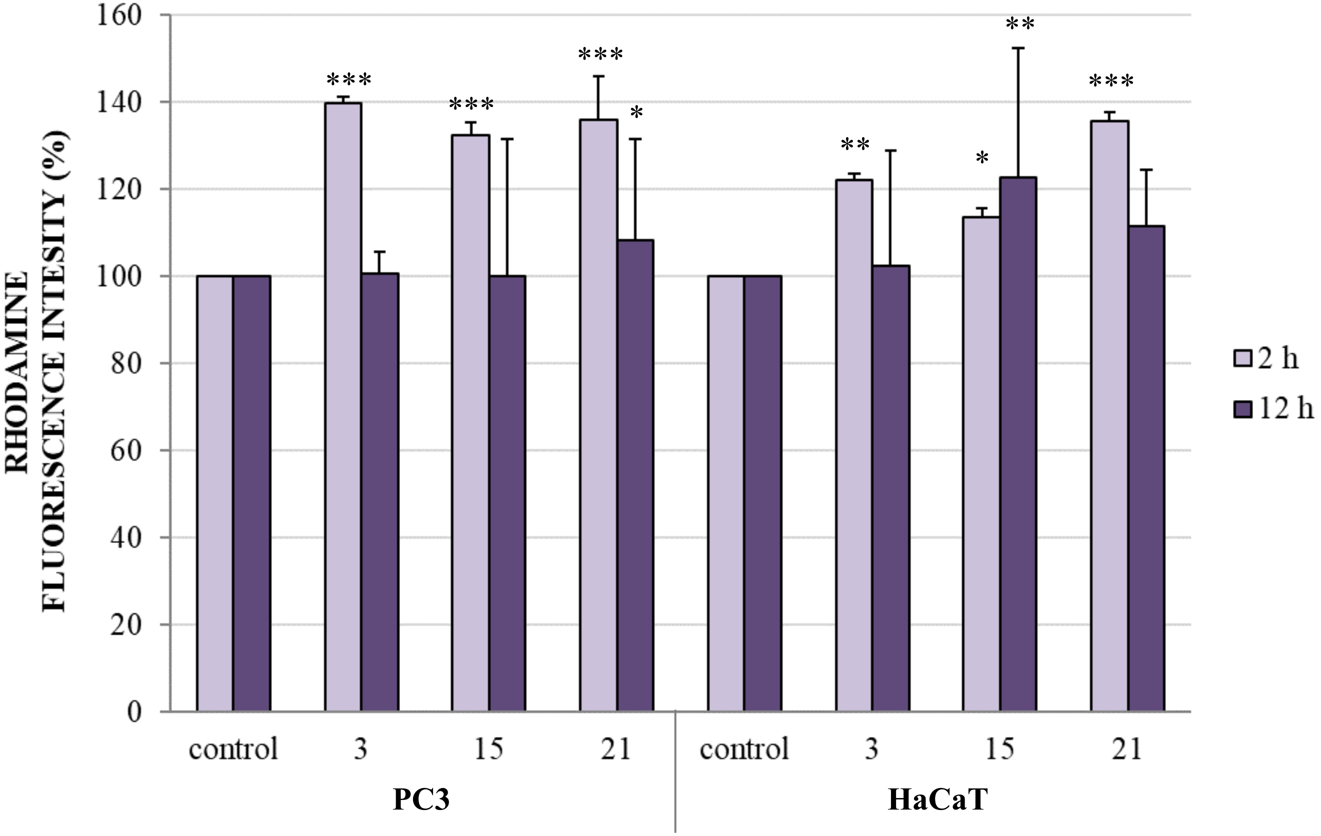

Figure S3. The effect of CP conjugates **3**, **15**, **21** on ROS production in PC3 and HaCaT cells. Cells were incubated with tested compounds at their IC_{50} concentration for 2 and 12 h. Fluorescence intensity (FI) of the probe was measured by rhodamine (5 μM). The results are expressed as mean ± SD of three experiments, each of them performed in triplicate. $^{***}p \leq 0.0001$, $^{**}p \leq 0.001$, $^{*}p \leq 0.01$, as compared to the control.

^{1}H and ^{13}C NMR spectra of synthesized Ciprofloxacin derivatives **1-21.**

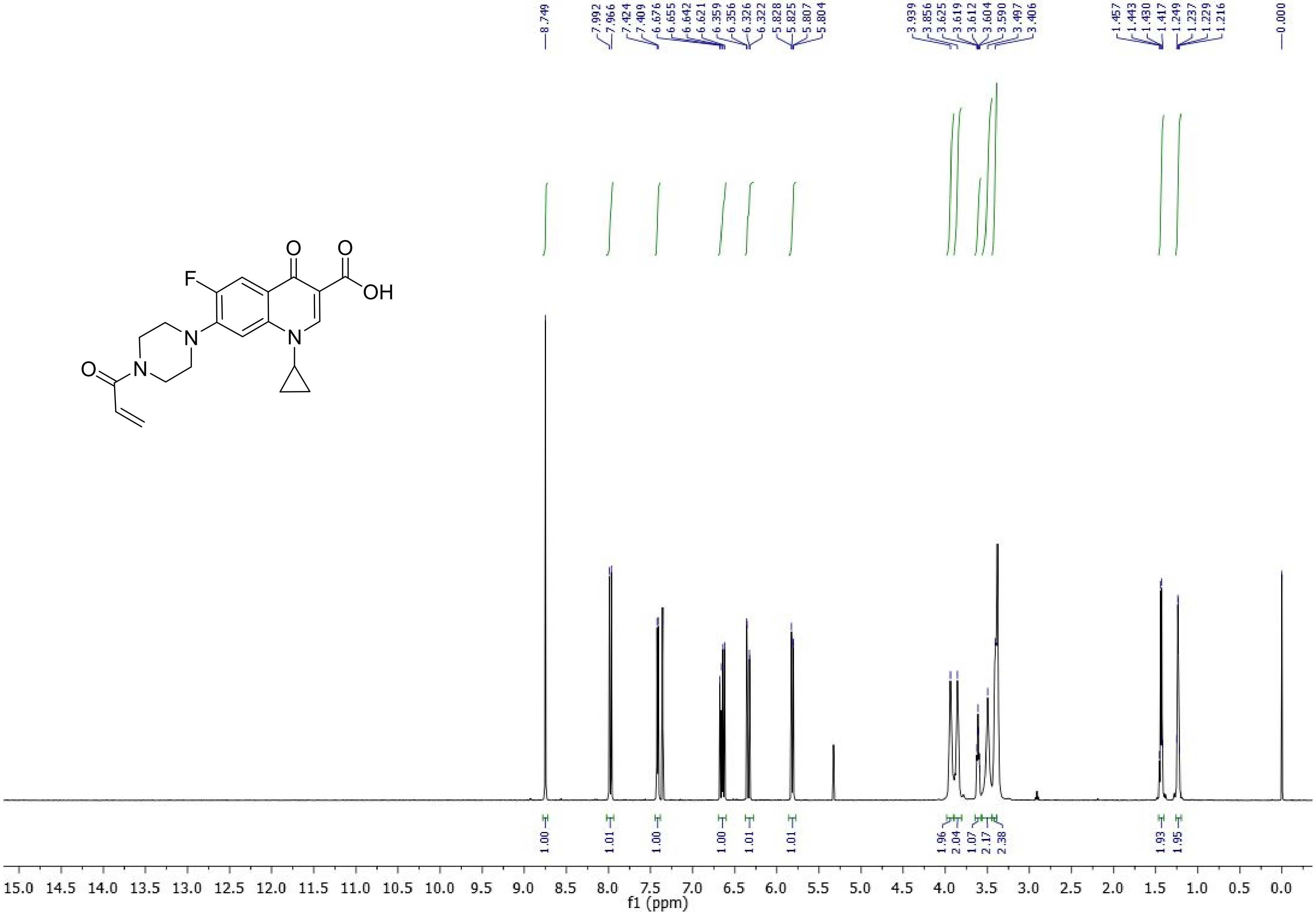
8.749
7.992
7.966
7.424
7.409
6.676
6.655
6.642
6.621
6.359
6.356
6.326
6.322
5.828
5.825
5.807
5.804
3.939
3.856
3.625
3.619
3.612
3.604
3.590
3.497
3.406
1.457
1.443
1.430
1.417
1.249
1.237
1.229
1.216
0.000
1.00
1.01
1.00
1.00
1.01
1.01
1.96
2.04
1.07
2.17
2.38
1.93
1.95
15.0 14.5 14.0 13.5 13.0 12.5 12.0 11.5 11.0 10.5 10.0 9.5 9.0 8.5 8.0 7.5 7.0 6.5 6.0 5.5 5.0 4.5 4.0 3.5 3.0 2.5 2.0 1.5 1.0 0.5 0.0
f1 (ppm)

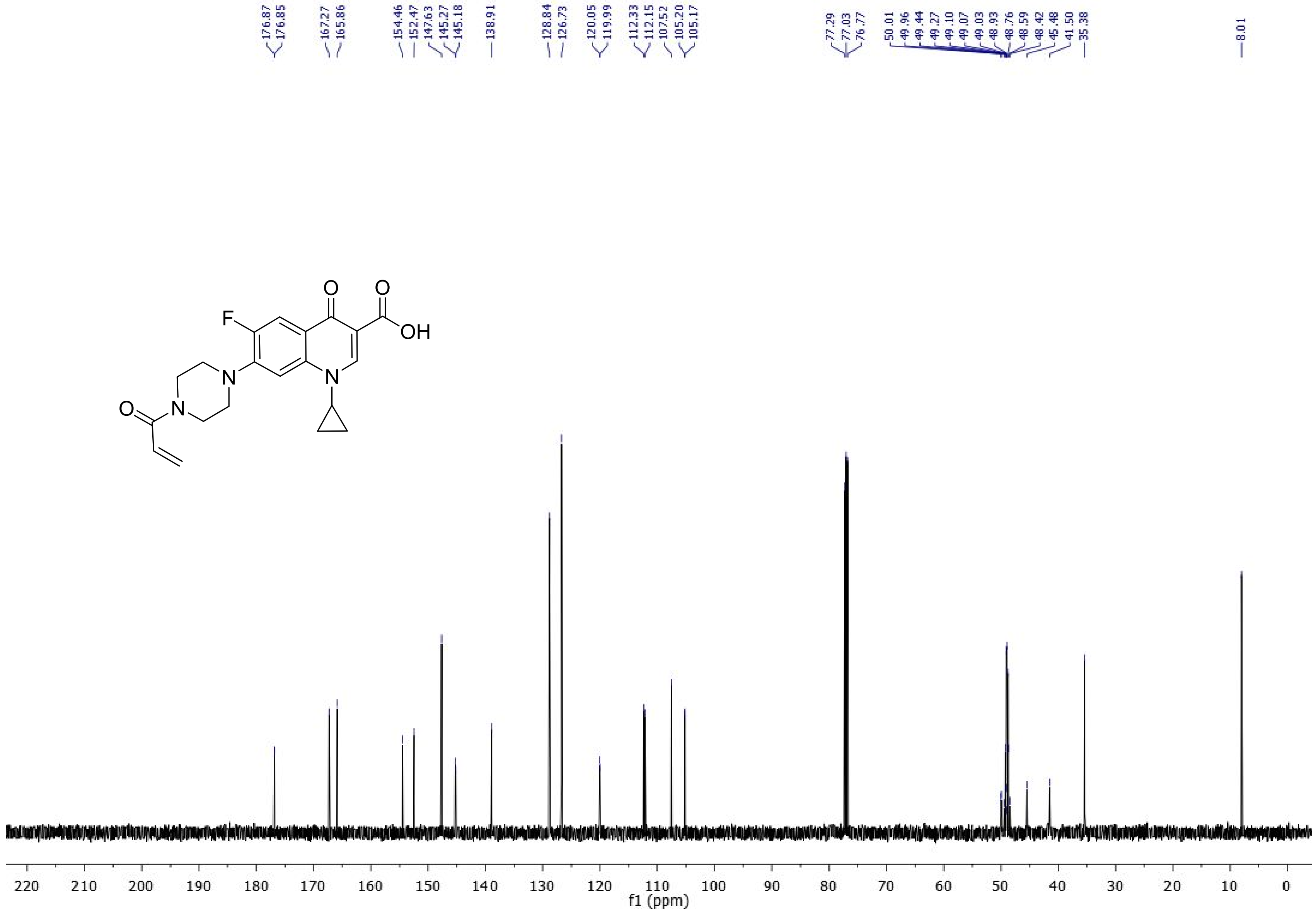
176.87
176.85
167.27
165.86
154.46
152.47
147.63
145.27
145.18
138.91
128.84
126.73
120.05
119.99
112.33
112.15
107.52
105.20
105.17
77.29
77.03
76.77
50.01
49.96
49.44
49.27
49.10
49.07
49.03
48.93
48.76
48.59
48.42
45.48
41.50
35.38
8.01
F
O
O
OH
N
N
O
N
220
210
200
190
180
170
160
150
140
130
120
110
100
90
80
70
60
50
40
30
20
10
0
f1 (ppm)

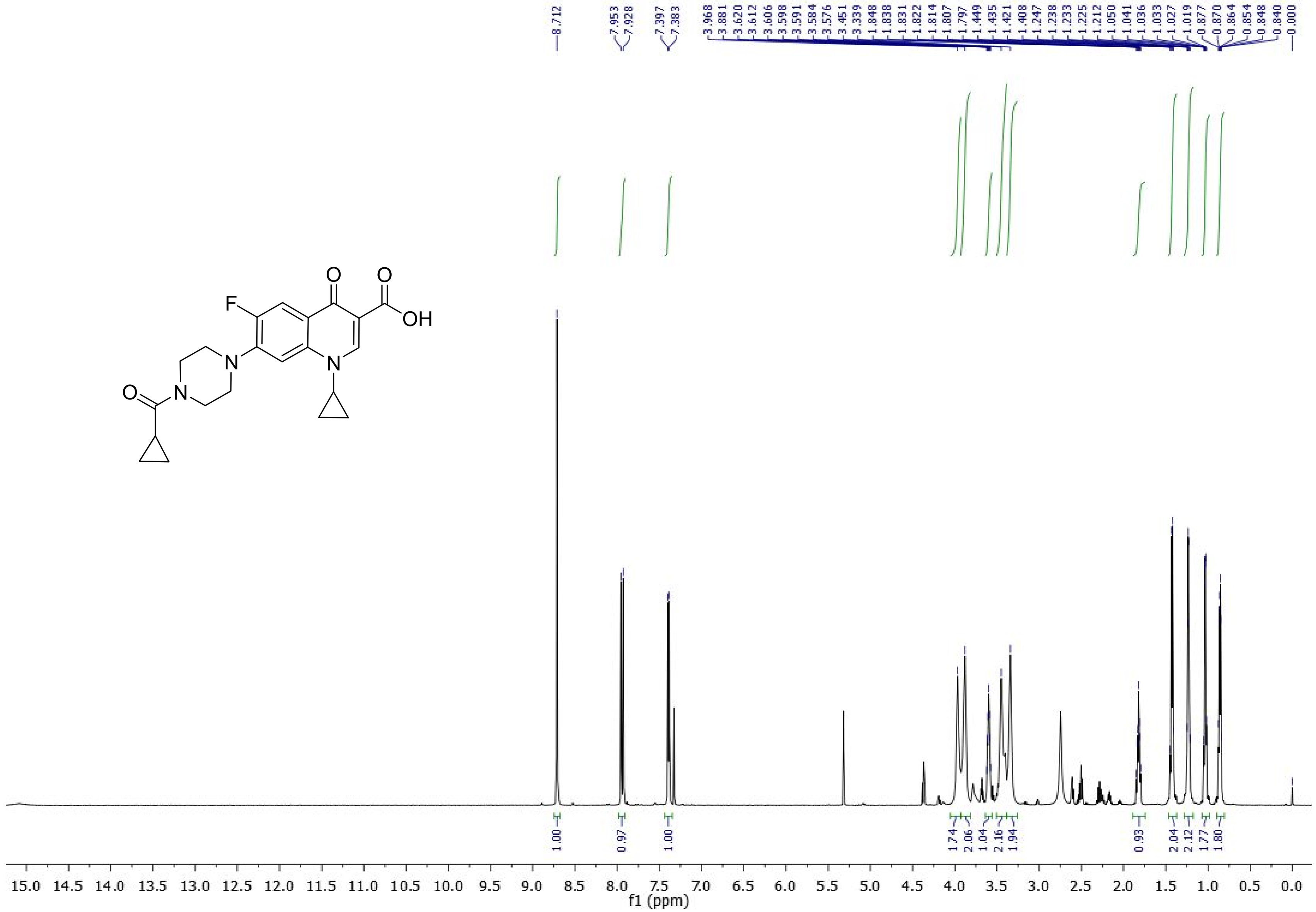

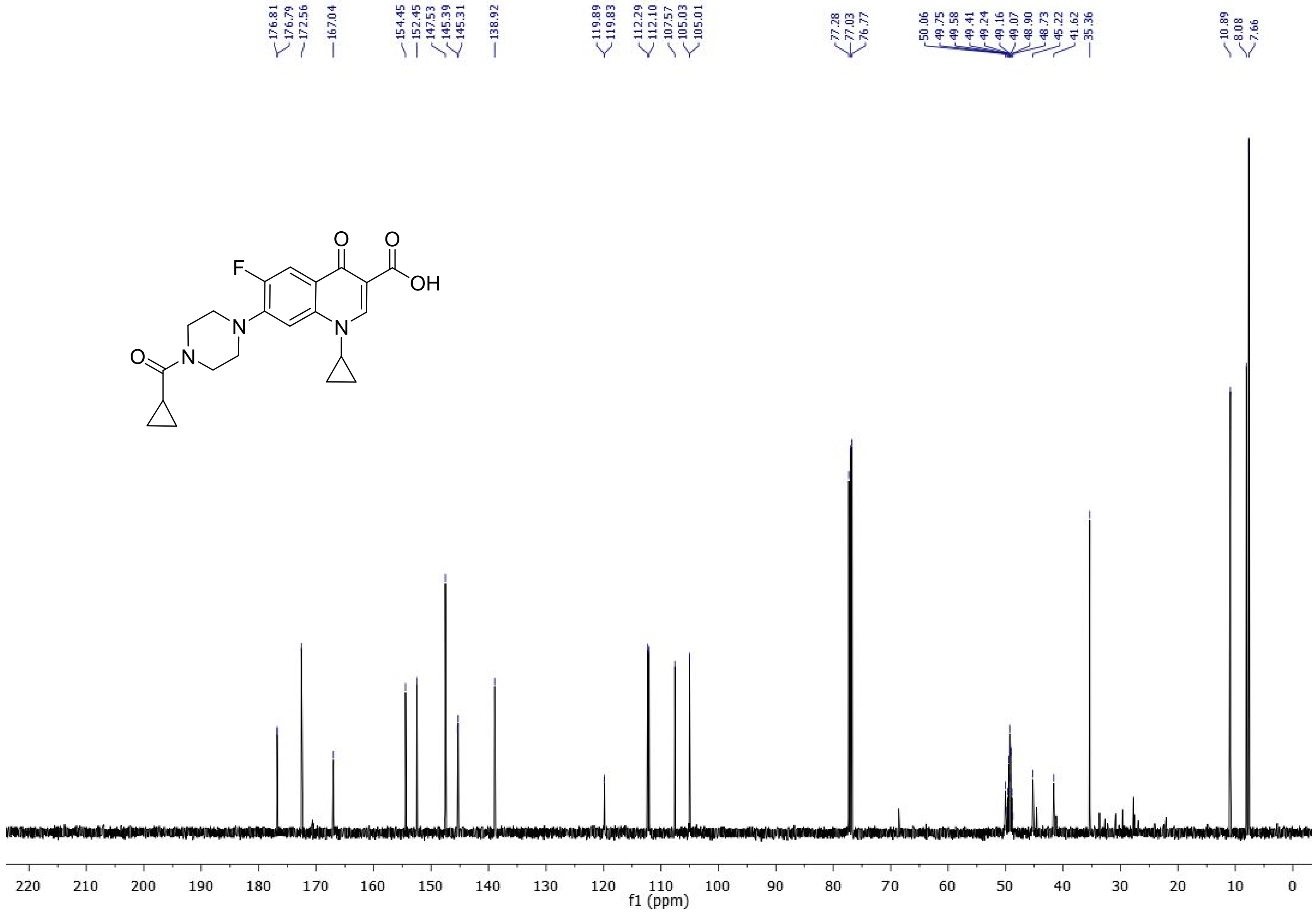
176.81
176.79
172.56
167.04
154.45
152.45
147.53
145.39
145.31
138.92
119.89
119.83
112.29
112.10
107.57
105.03
105.01
77.28
77.03
76.77
50.06
49.75
49.58
49.41
49.24
49.16
49.07
48.90
48.73
45.22
41.62
35.36
10.89
8.08
7.66
F
O
O
OH
N
N
N
O
220
210
200
190
180
170
160
150
140
130
120
110
100
90
80
70
60
50
40
30
20
10
0
f1 (ppm)

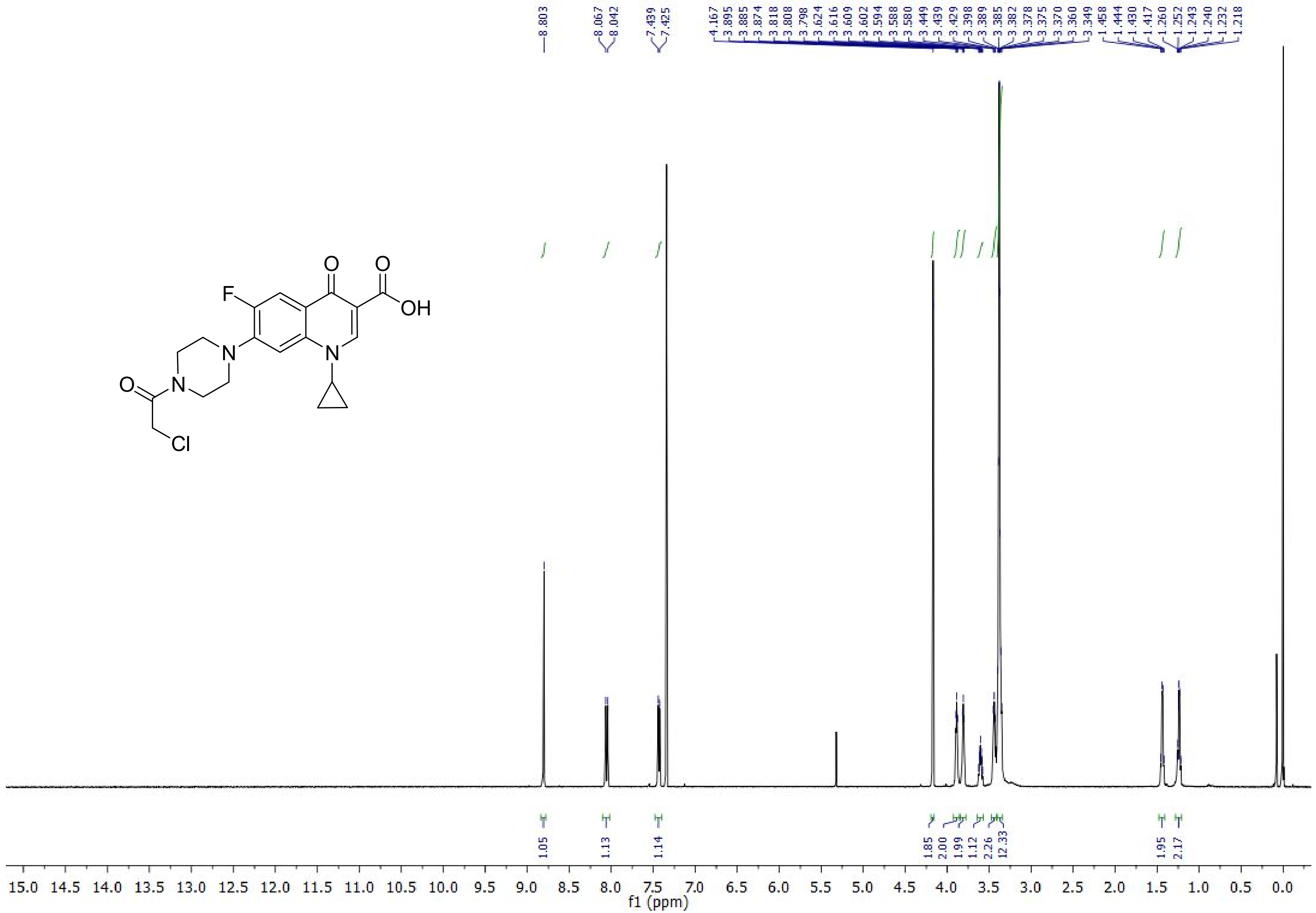
F
O
O
OH
N
N
O
N
Cl
8.803
8.067
8.042
7.439
7.425
4.167
3.895
3.885
3.874
3.818
3.808
3.798
3.624
3.616
3.609
3.602
3.594
3.588
3.580
3.449
3.439
3.429
3.398
3.389
3.385
3.382
3.378
3.375
3.370
3.360
3.349
1.458
1.444
1.430
1.417
1.260
1.252
1.243
1.240
1.232
1.218
1.05
1.13
1.14
1.85
2.00
1.99
1.12
2.26
12.33
1.95
2.17
15.0 14.5 14.0 13.5 13.0 12.5 12.0 11.5 11.0 10.5 10.0 9.5 9.0 8.5 8.0 7.5 7.0 6.5 6.0 5.5 5.0 4.5 4.0 3.5 3.0 2.5 2.0 1.5 1.0 0.5 0.0
f1 (ppm)

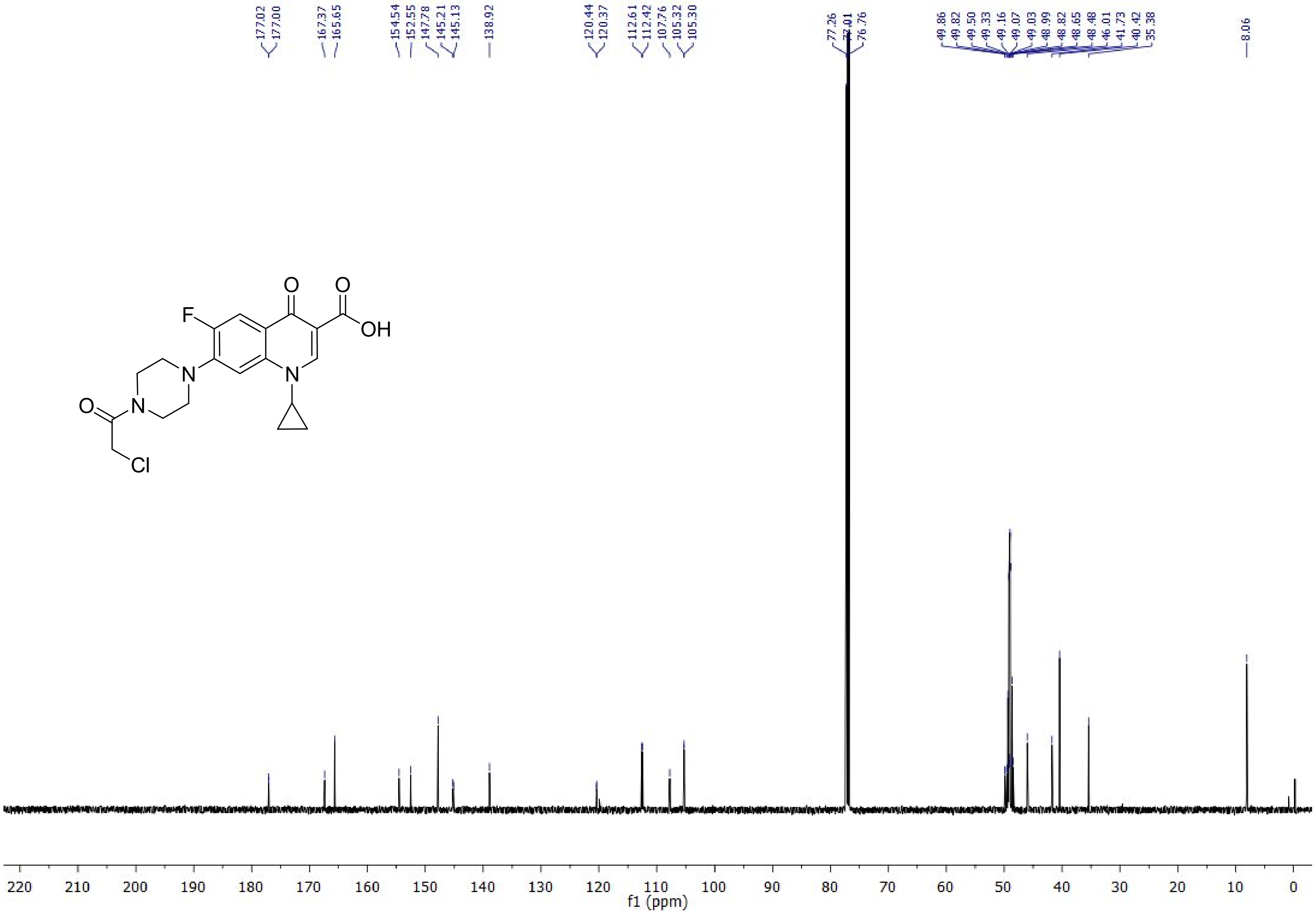
177.02
177.00
167.37
165.65
154.54
152.55
147.78
145.21
145.13
138.92
120.44
120.37
112.61
112.42
107.76
105.32
105.30
77.26
77.01
76.76
49.86
49.82
49.50
49.33
49.16
49.07
49.03
48.99
48.82
48.65
48.48
46.01
41.73
40.42
35.38
8.06
F
O
O
OH
N
N
N
O
Cl
220
210
200
190
180
170
160
150
140
130
120
110
100
90
80
70
60
50
40
30
20
10
0
f1 (ppm)

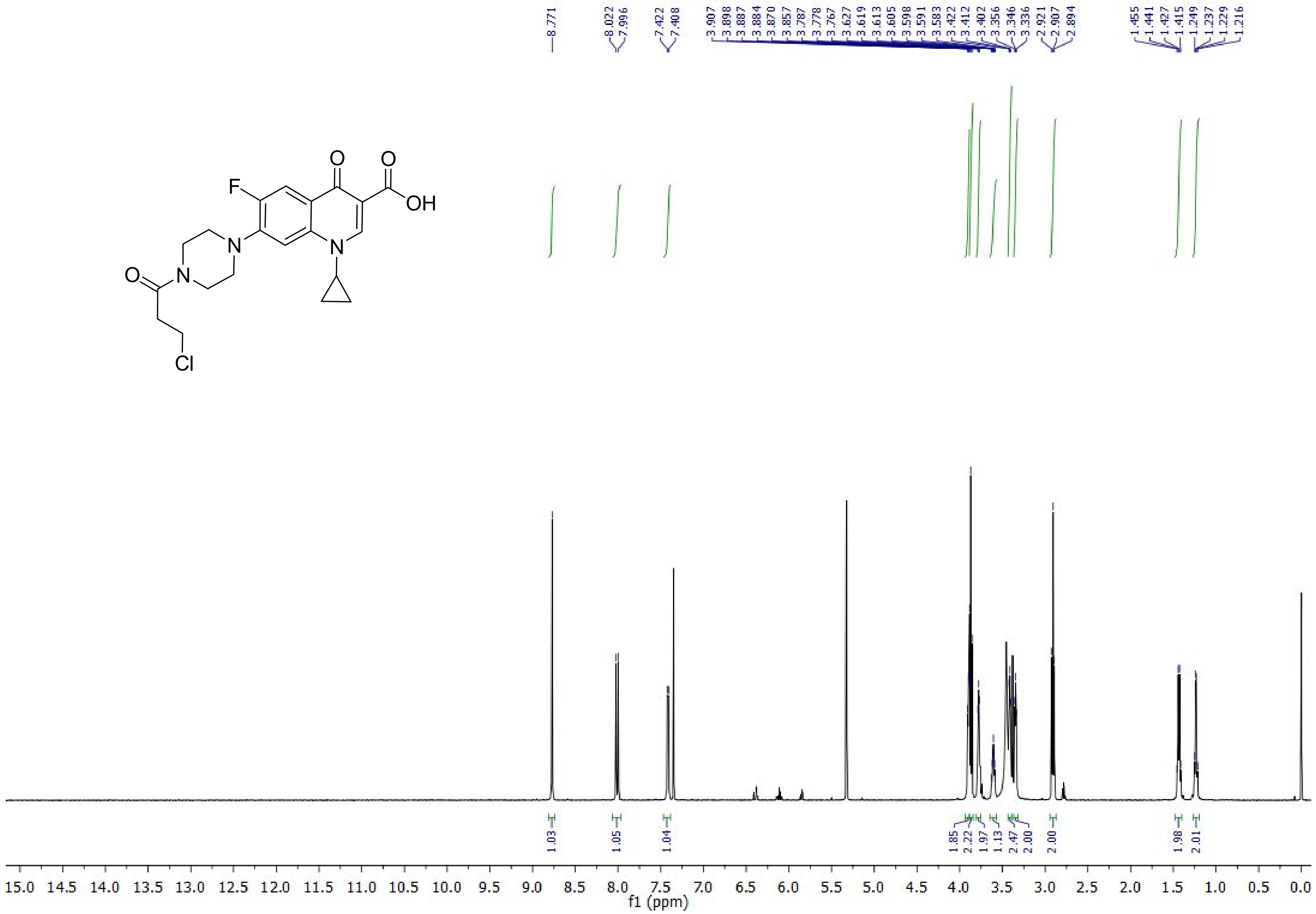
8.771
8.022
7.996
7.422
7.408
3.907
3.898
3.887
3.884
3.870
3.857
3.787
3.778
3.767
3.627
3.619
3.613
3.605
3.598
3.591
3.583
3.422
3.412
3.402
3.356
3.346
3.336
2.921
2.907
2.894
1.455
1.441
1.427
1.415
1.249
1.237
1.229
1.216
1.03
1.05
1.04
1.85
2.22
1.97
1.13
2.47
2.00
2.00
1.98
2.01
15.0 14.5 14.0 13.5 13.0 12.5 12.0 11.5 11.0 10.5 10.0 9.5 9.0 8.5 8.0 7.5 7.0 6.5 6.0 5.5 5.0 4.5 4.0 3.5 3.0 2.5 2.0 1.5 1.0 0.5 0.0
f1 (ppm)

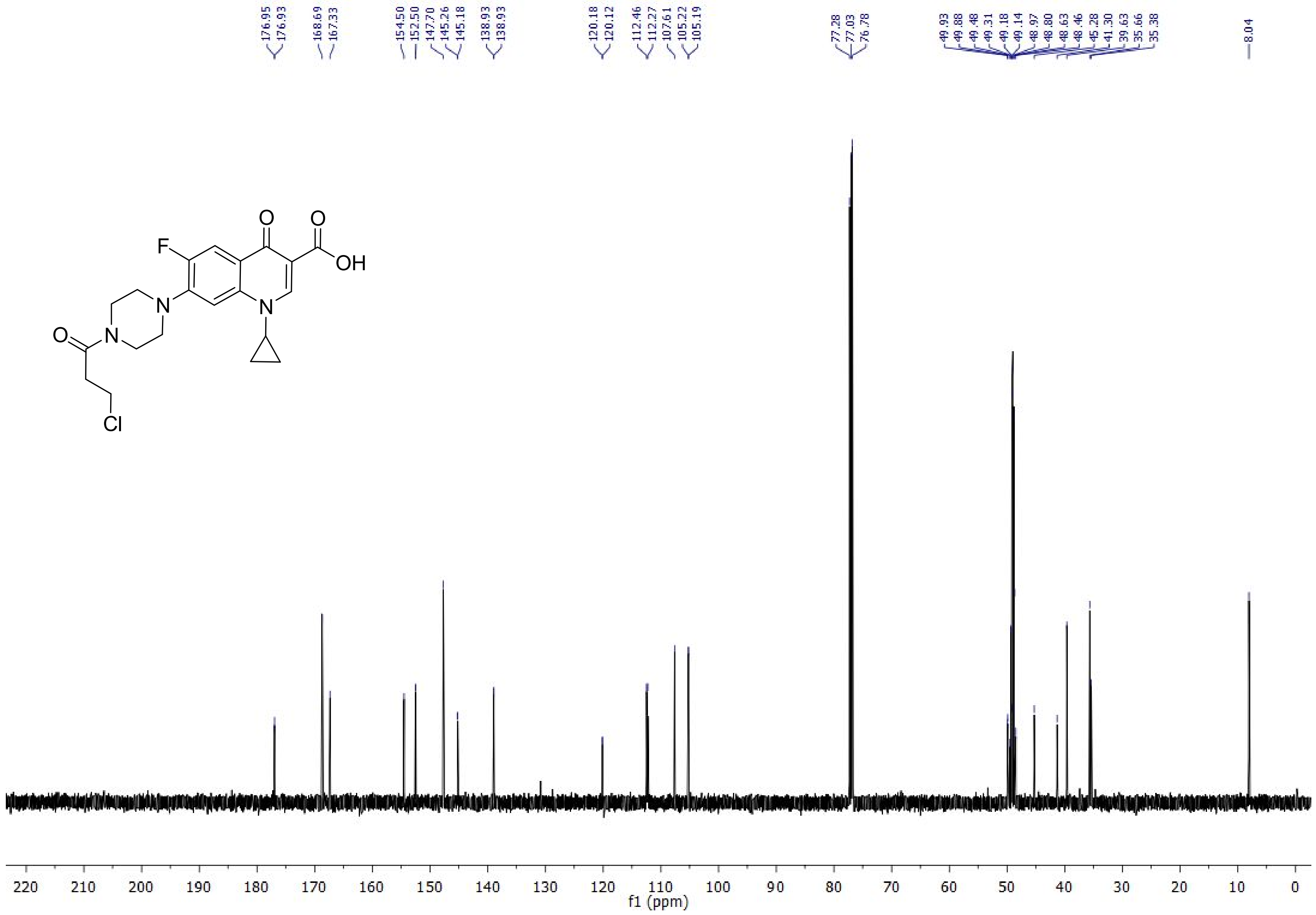
176.95
176.93
168.69
167.33
154.50
152.50
147.70
145.26
145.18
138.93
138.93
120.18
120.12
112.46
112.27
107.61
105.22
105.19
77.28
77.03
76.78
49.93
49.88
49.48
49.31
49.18
49.14
48.97
48.80
48.63
48.46
45.28
41.30
39.63
35.66
35.38
8.04
F
O
O
OH
N
N
O
N
Cl
220 210 200 190 180 170 160 150 140 130 120 110 100 90 80 70 60 50 40 30 20 10 0
f1 (ppm)

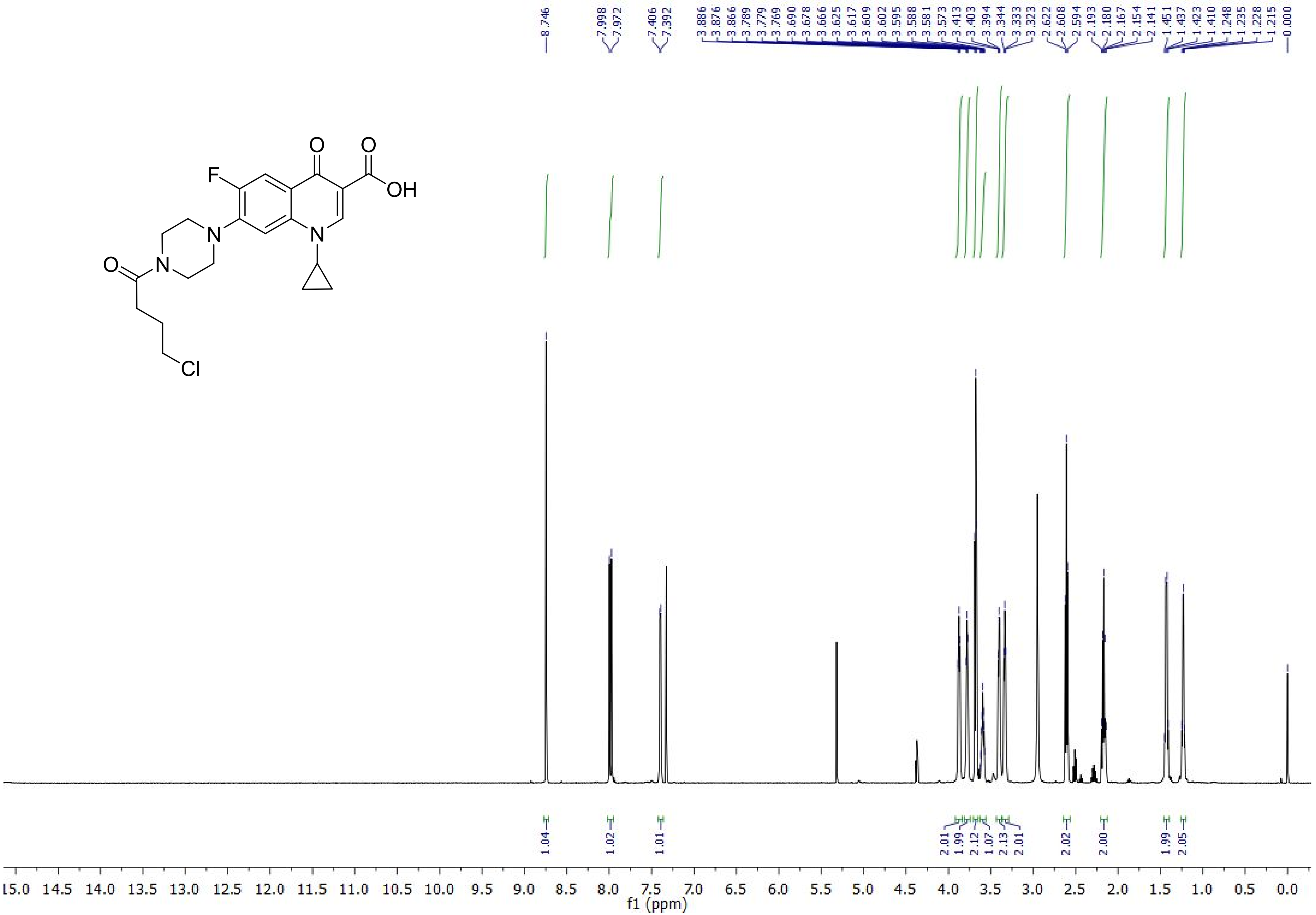

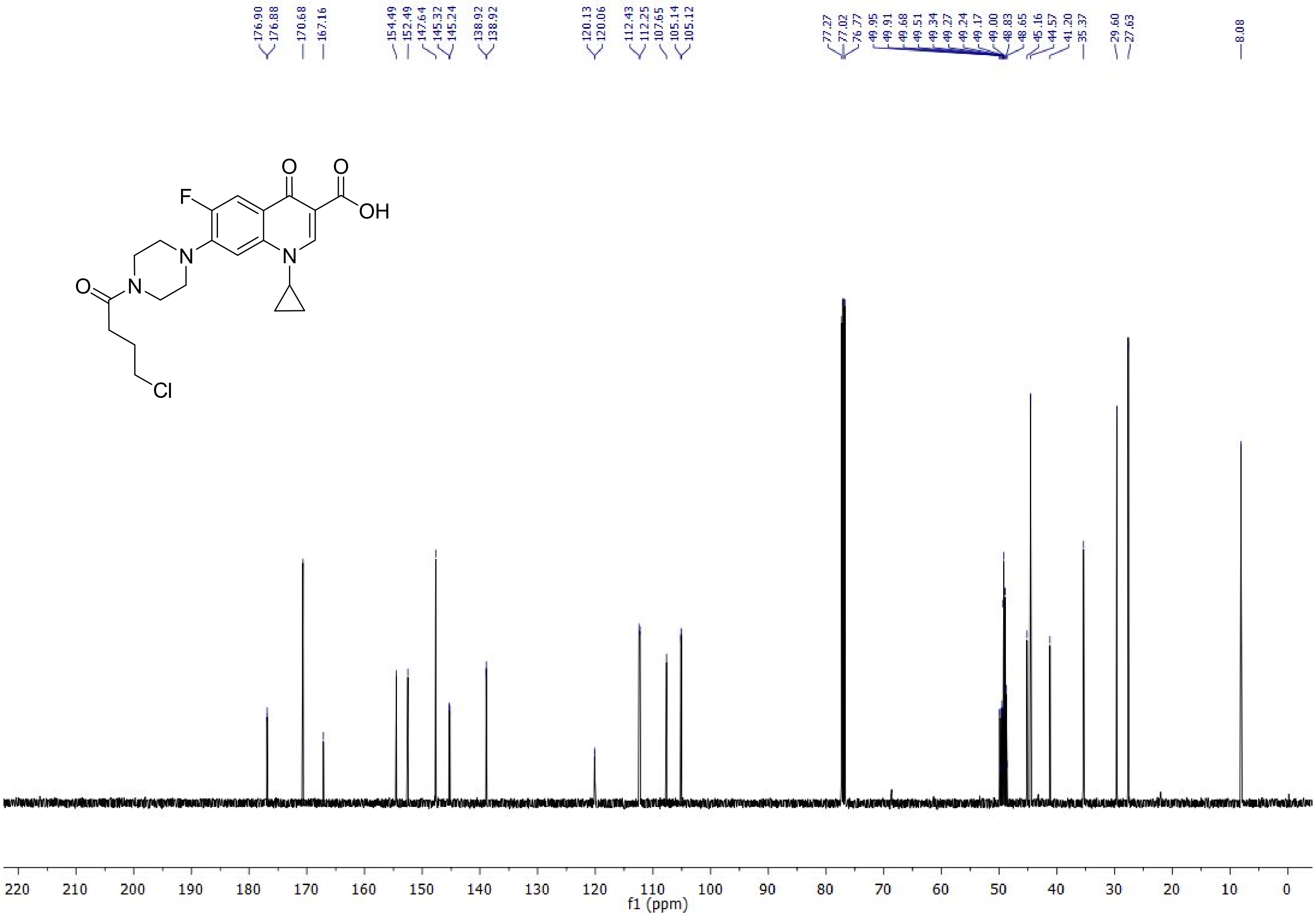
176.90
176.88
170.68
167.16
154.49
152.49
147.64
145.32
145.24
138.92
138.92
120.13
120.06
112.43
112.25
107.65
105.14
105.12
77.27
77.02
76.77
49.95
49.91
49.68
49.51
49.34
49.27
49.24
49.17
49.00
48.83
48.65
45.16
44.57
41.20
35.37
29.60
27.63
8.08
F
O
O
OH
N
N
N
O
Cl
220
210
200
190
180
170
160
150
140
130
120
110
100
90
80
70
60
50
40
30
20
10
0
f1 (ppm)

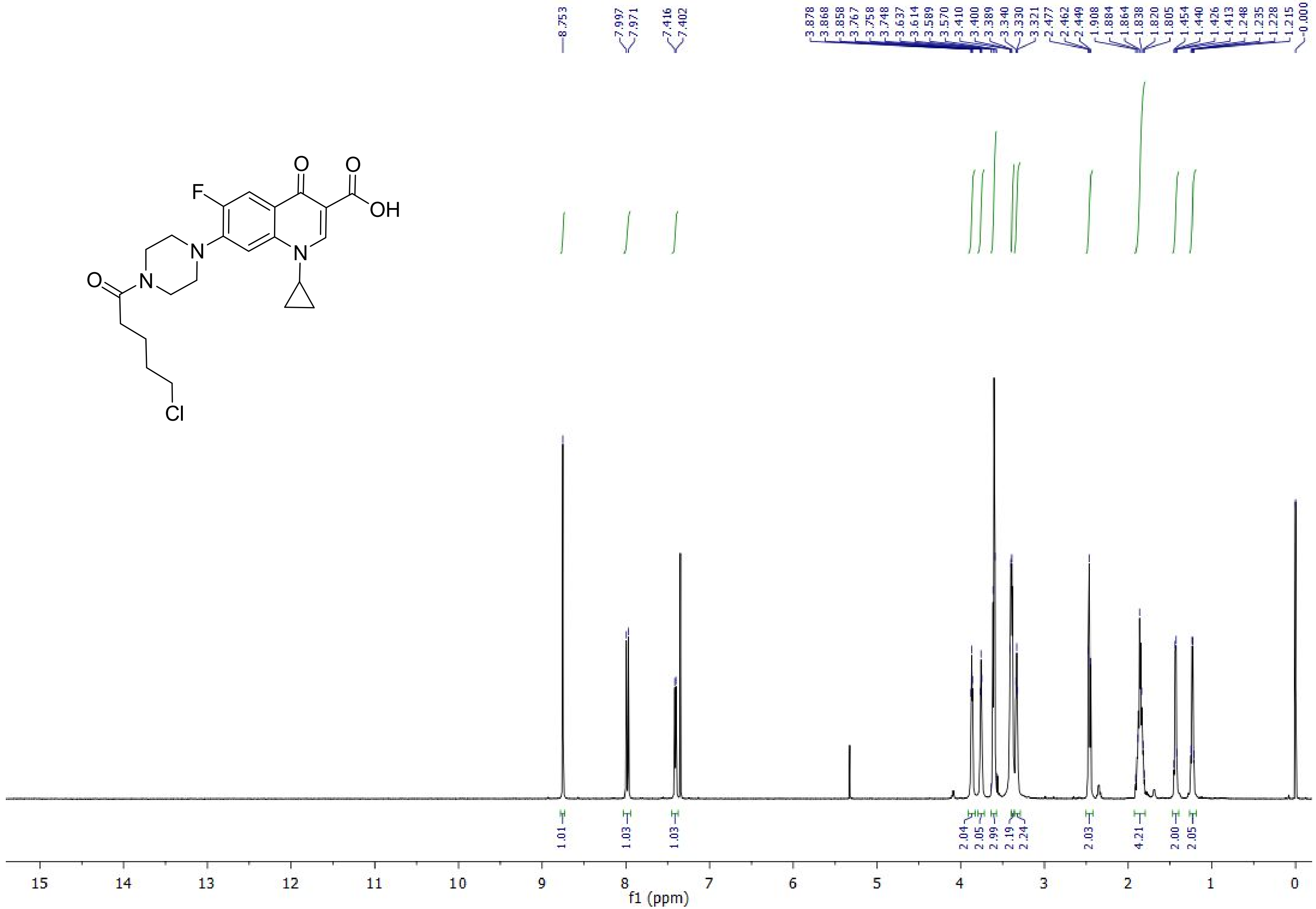
F
O
O
OH
N
N
O
N
Cl
8.753
7.997
7.971
7.416
7.402
3.878
3.868
3.858
3.767
3.758
3.748
3.637
3.614
3.589
3.570
3.410
3.400
3.389
3.340
3.330
3.321
2.477
2.462
2.449
1.908
1.884
1.864
1.838
1.820
1.805
1.454
1.440
1.426
1.413
1.248
1.235
1.228
1.215
0.000
1.01
1.03
1.03
2.04
2.05
2.99
2.19
2.24
2.03
4.21
2.00
2.05
15
14
13
12
11
10
9
8
7
6
5
4
3
2
1
0
f1 (ppm)

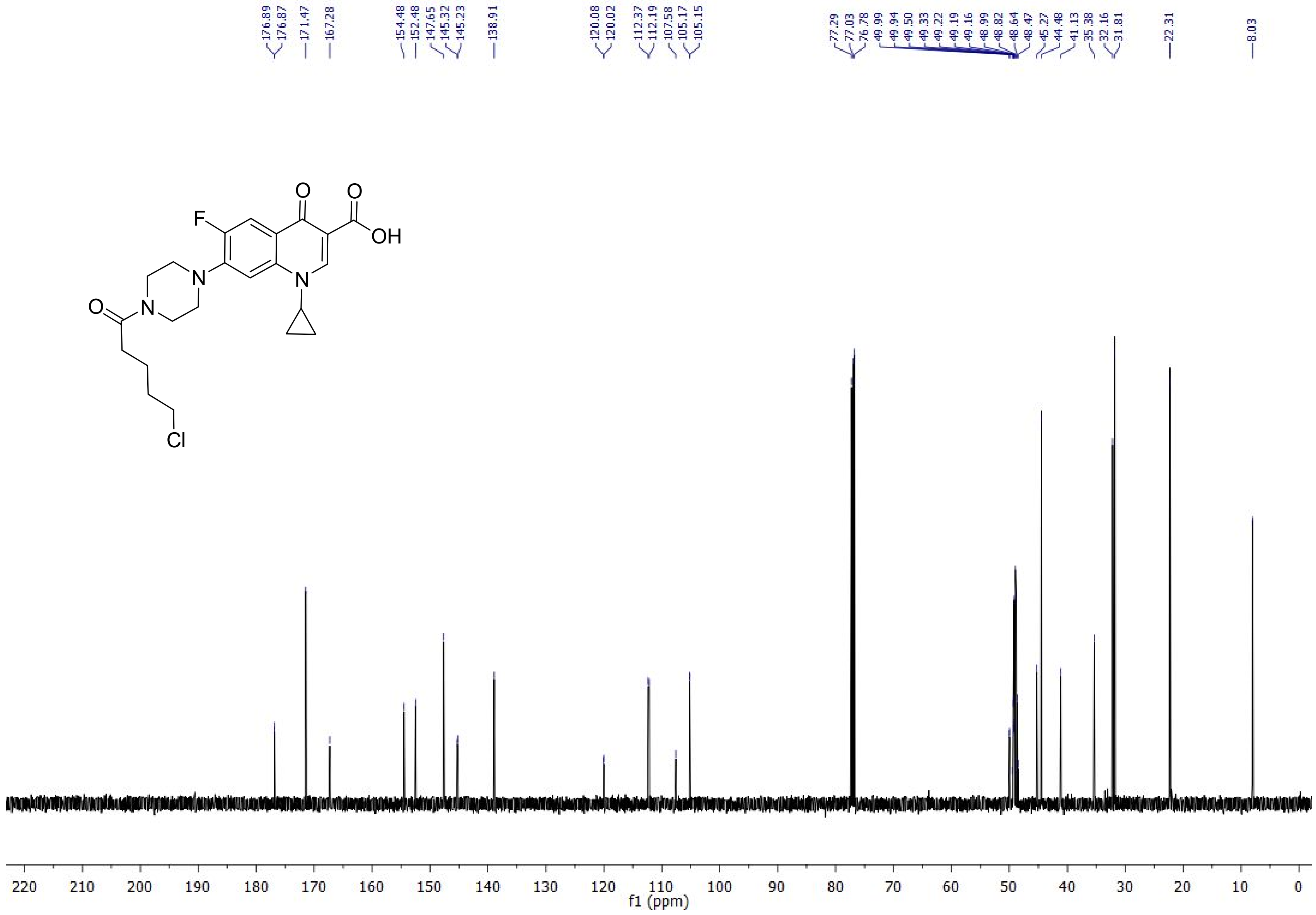
176.89
176.87
171.47
167.28
154.48
152.48
147.65
145.32
145.23
138.91
120.08
120.02
112.37
112.19
107.58
105.17
105.15
77.29
77.03
76.78
49.99
49.94
49.50
49.33
49.22
49.19
49.16
48.99
48.82
48.64
48.47
45.27
44.48
41.13
35.38
32.16
31.81
22.31
8.03
F
O
O
OH
N
N
N
O
Cl
f1 (ppm)

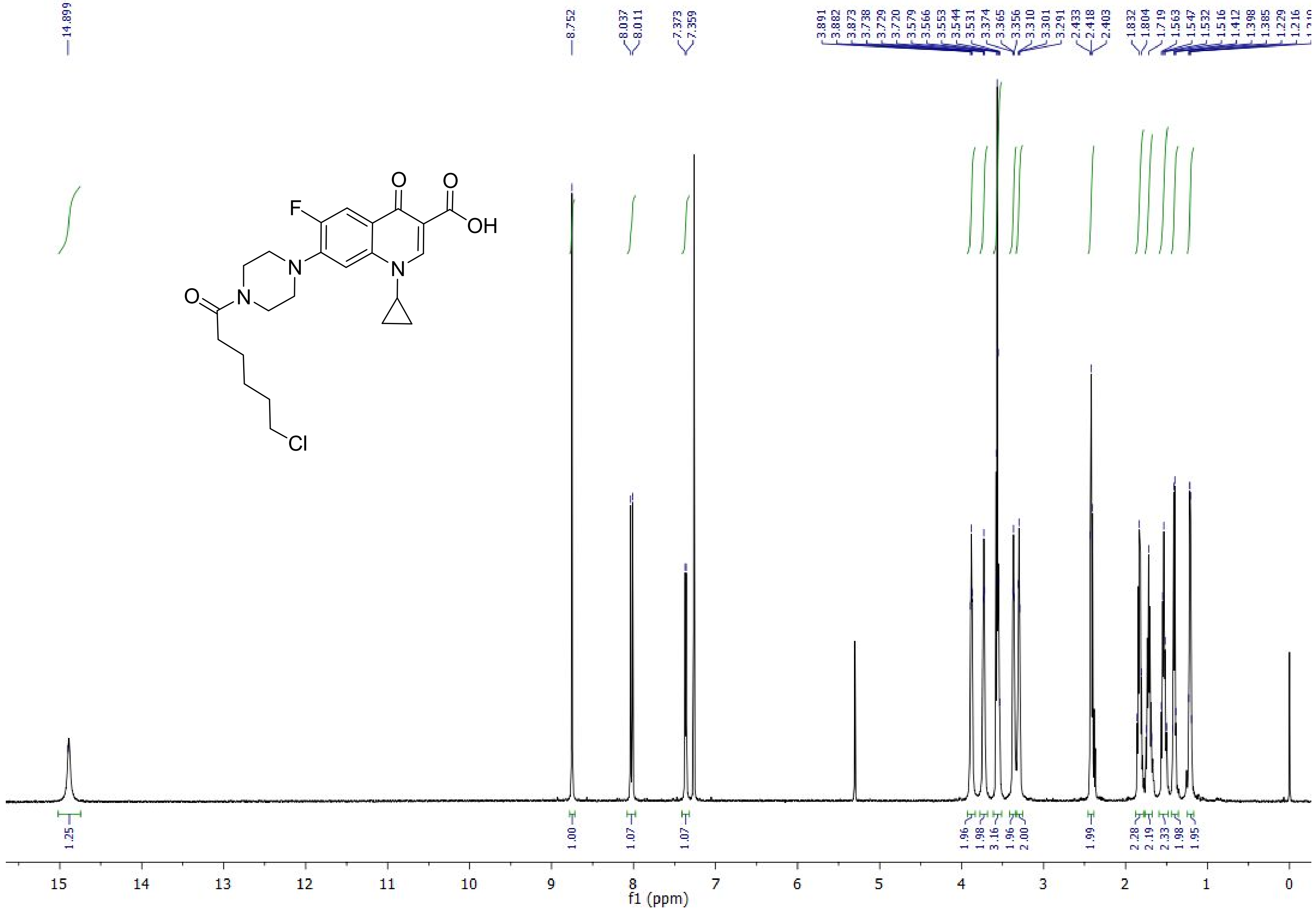

f1 (ppm)

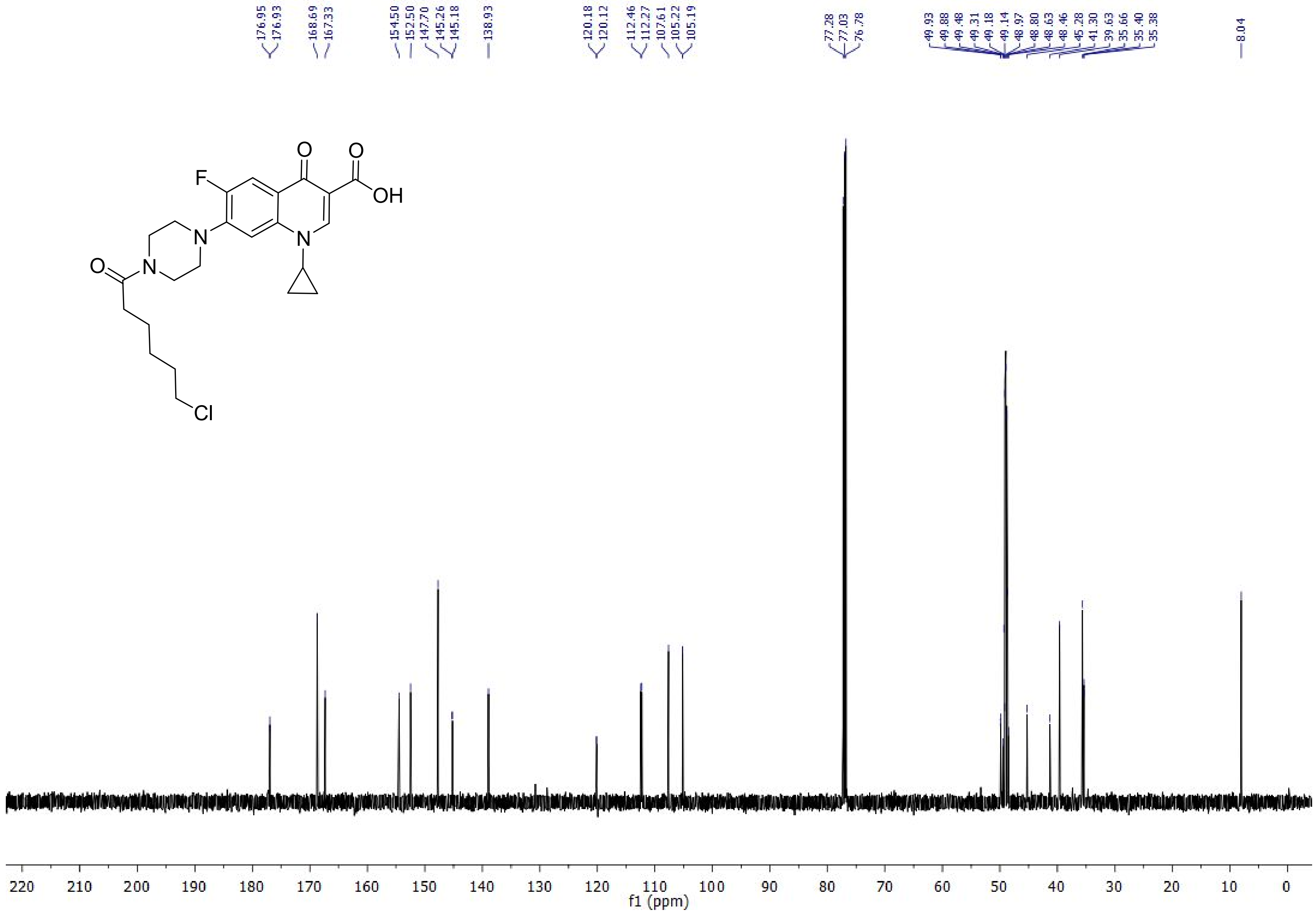
176.95
176.93
168.69
167.33
154.50
152.50
147.70
145.26
145.18
138.93
120.18
120.12
112.46
112.27
107.61
105.22
105.19
77.28
77.03
76.78
49.93
49.88
49.48
49.31
49.18
49.14
48.97
48.80
48.63
48.46
45.28
41.30
39.63
35.66
35.40
35.38
8.04
F
O
O
OH
N
N
O
N
Cl
f1 (ppm)

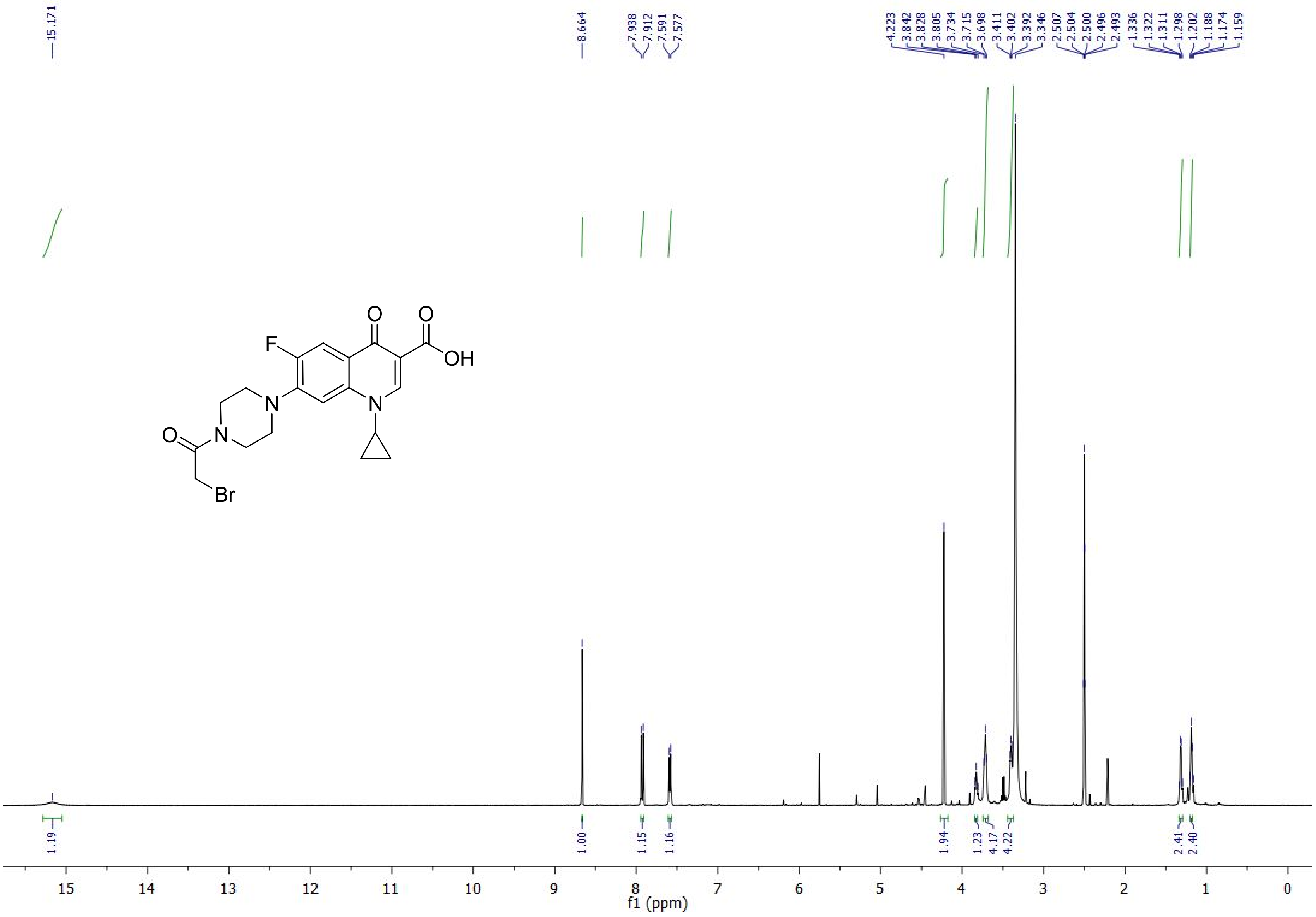

15.171
8.664
7.938
7.912
7.591
7.577
4.223
3.842
3.828
3.805
3.734
3.715
3.698
3.411
3.402
3.392
3.346
2.507
2.504
2.500
2.496
2.493
1.336
1.322
1.311
1.298
1.202
1.188
1.174
1.159
1.19
1.00
1.15
1.16
1.94
1.23
4.17
4.22
2.41
2.40
f1 (ppm)
F
O
O
OH
N
N
O
N
Br

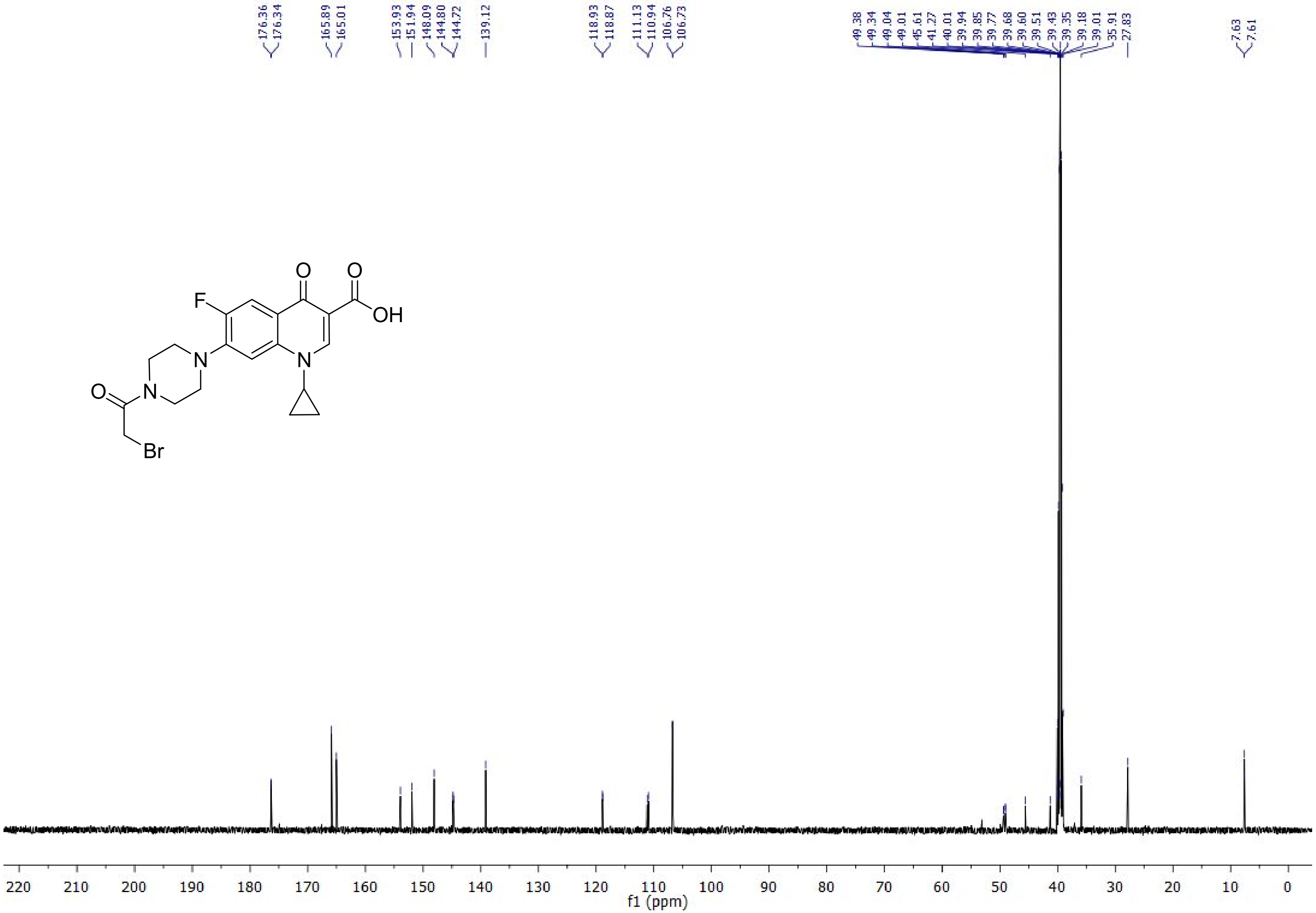
176.36
176.34
165.89
165.01
153.93
151.94
148.09
144.80
144.72
139.12
118.93
118.87
111.13
110.94
106.76
106.73
49.38
49.34
49.04
49.01
45.61
41.27
40.01
39.94
39.85
39.77
39.68
39.60
39.51
39.43
39.35
39.18
39.01
35.91
27.83
7.63
7.61
F
O
O
OH
N
N
N
O
Br
220 210 200 190 180 170 160 150 140 130 120 110 100 90 80 70 60 50 40 30 20 10 0
f1 (ppm)

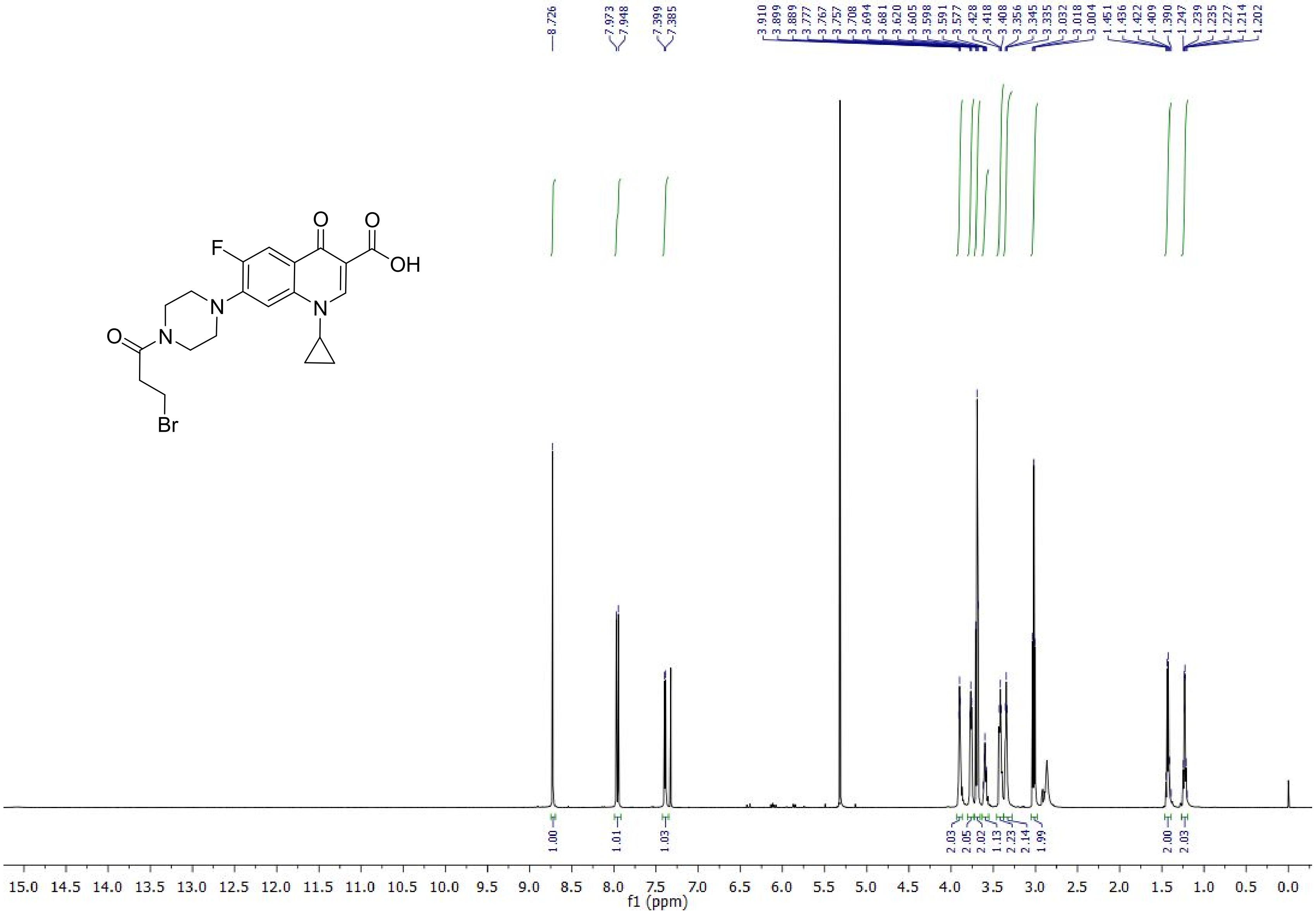
F
O
O
OH
N
N
N
O
Br
8.726
7.973
7.948
7.399
7.385
3.910
3.899
3.889
3.777
3.767
3.757
3.708
3.694
3.681
3.620
3.605
3.598
3.591
3.577
3.428
3.418
3.408
3.356
3.345
3.335
3.032
3.018
3.004
1.451
1.436
1.422
1.409
1.390
1.247
1.239
1.235
1.227
1.214
1.202
1.00
1.01
1.03
2.03
2.05
2.02
1.13
2.23
2.14
1.99
2.00
2.03
15.0 14.5 14.0 13.5 13.0 12.5 12.0 11.5 11.0 10.5 10.0 9.5 9.0 8.5 8.0 7.5 7.0 6.5 6.0 5.5 5.0 4.5 4.0 3.5 3.0 2.5 2.0 1.5 1.0 0.5 0.0
f1 (ppm)

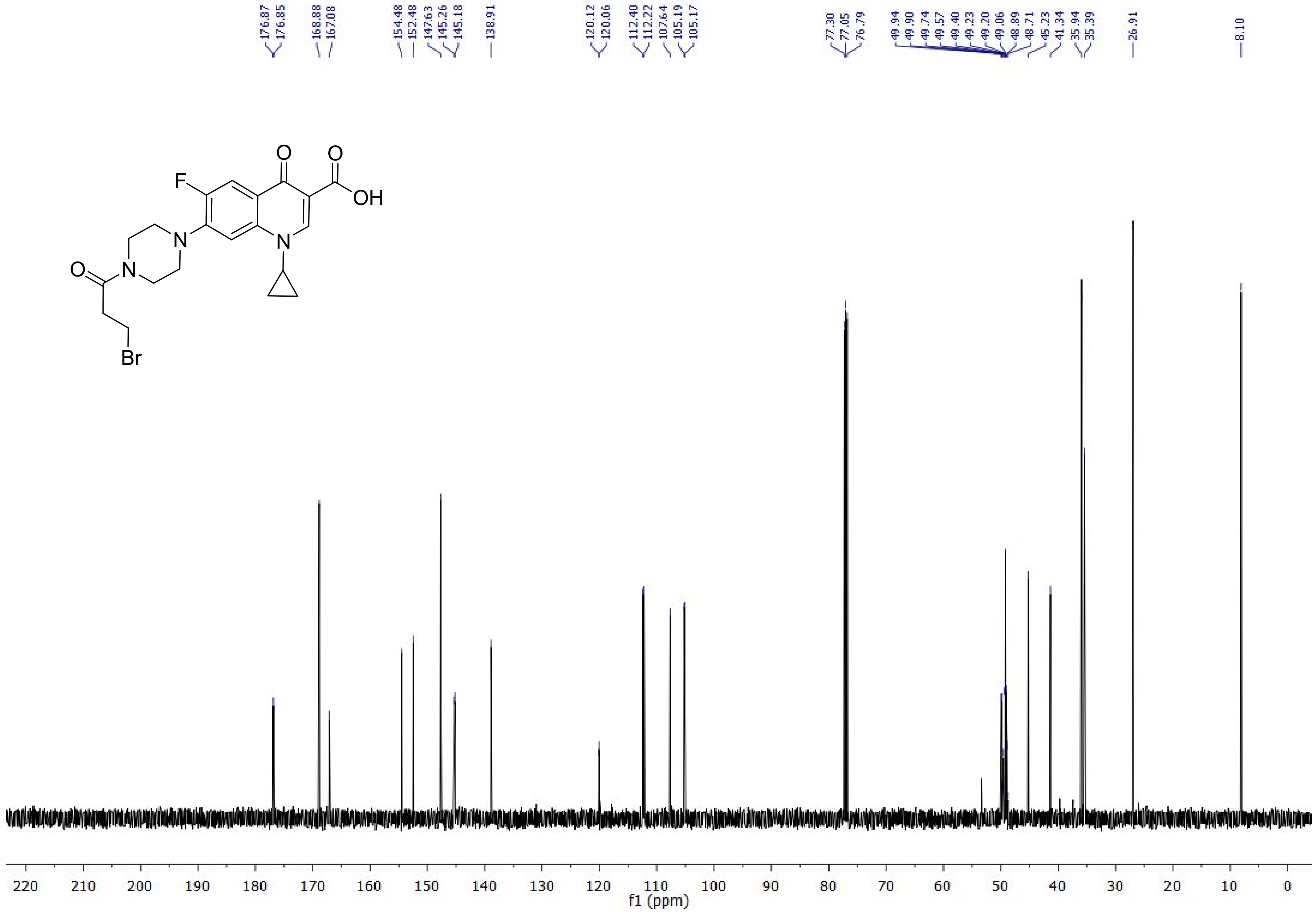
176.87
176.85
168.88
167.08
154.48
152.48
147.63
145.26
145.18
138.91
120.12
120.06
112.40
112.22
107.64
105.19
105.17
77.30
77.05
76.79
49.94
49.90
49.74
49.57
49.40
49.23
49.20
49.06
48.89
48.71
45.23
41.34
35.94
35.39
26.91
8.10
F
O
O
OH
N
N
N
O
Br
220 210 200 190 180 170 160 150 140 130 120 110 100 90 80 70 60 50 40 30 20 10 0
f1 (ppm)

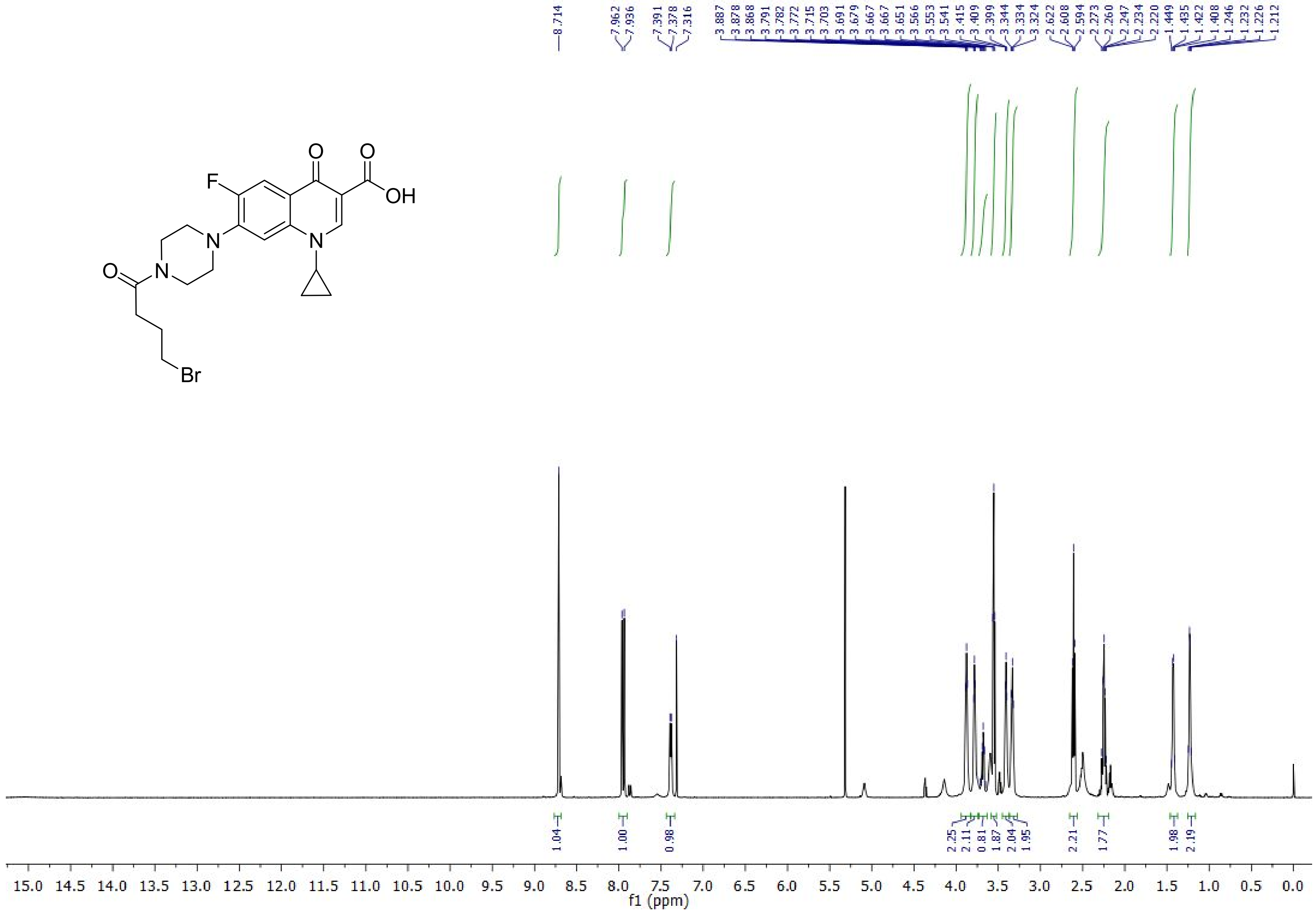
F
O
O
OH
N
N
N
O
Br
8.714
7.962
7.936
7.391
7.378
7.316
3.887
3.878
3.868
3.791
3.782
3.772
3.715
3.703
3.691
3.679
3.667
3.667
3.651
3.566
3.553
3.541
3.415
3.409
3.399
3.344
3.334
3.324
2.622
2.608
2.594
2.273
2.260
2.247
2.234
2.220
1.449
1.435
1.422
1.408
1.246
1.232
1.226
1.212
1.04
1.00
0.98
2.25
2.11
0.81
1.87
2.04
1.95
2.21
1.77
1.98
2.19
15.0 14.5 14.0 13.5 13.0 12.5 12.0 11.5 11.0 10.5 10.0 9.5 9.0 8.5 8.0 7.5 7.0 6.5 6.0 5.5 5.0 4.5 4.0 3.5 3.0 2.5 2.0 1.5 1.0 0.5 0.0
f1 (ppm)

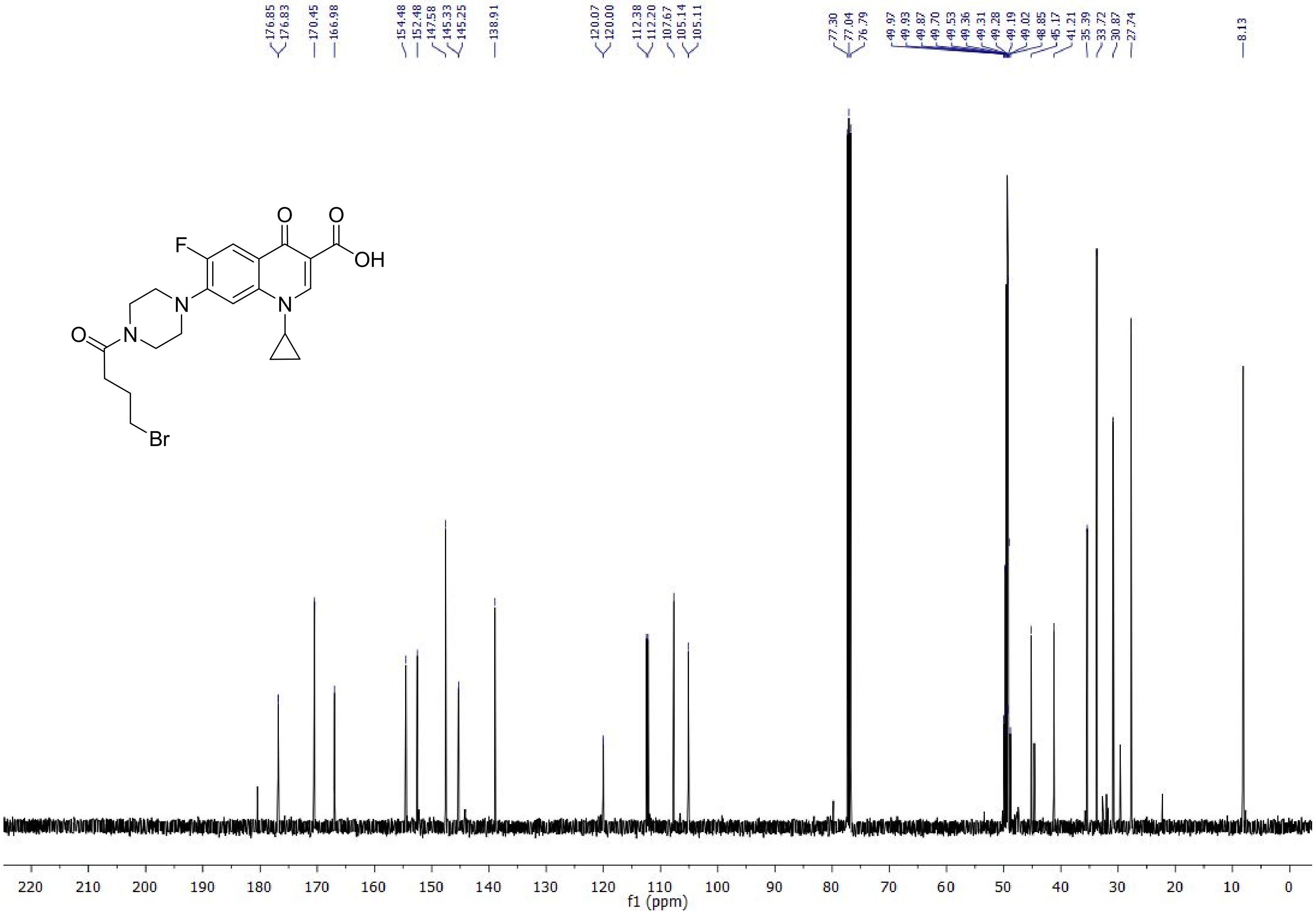
176.85
176.83
170.45
166.98
154.48
152.48
147.58
145.33
145.25
138.91
120.07
120.00
112.38
112.20
107.67
105.14
105.11
77.30
77.04
76.79
49.97
49.93
49.87
49.70
49.53
49.36
49.31
49.28
49.19
49.02
48.85
45.17
41.21
35.39
33.72
30.87
27.74
8.13
F
O
O
OH
N
N
N
O
Br
220 210 200 190 180 170 160 150 140 130 120 110 100 90 80 70 60 50 40 30 20 10 0
f1 (ppm)

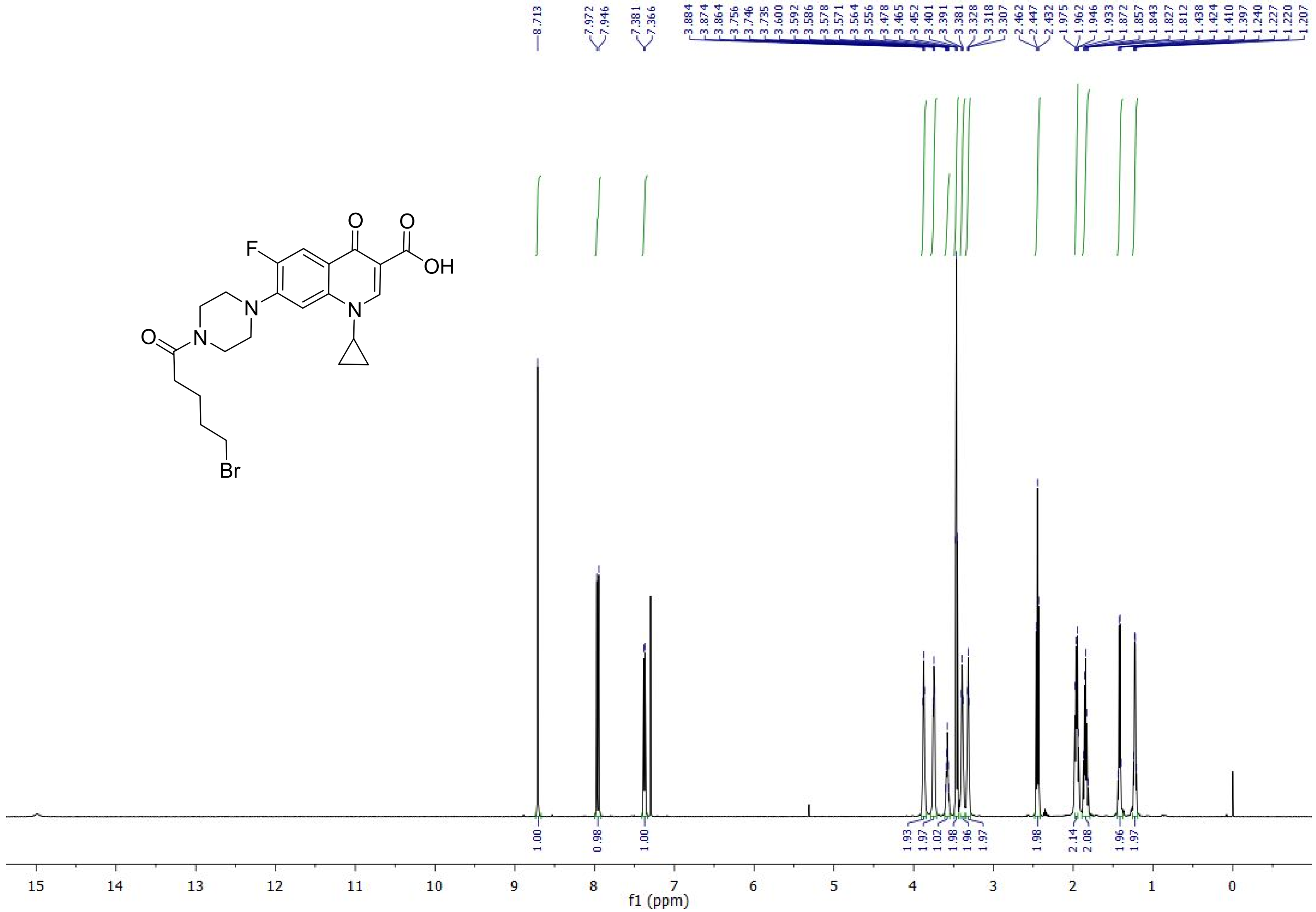
8.713
7.972
7.946
7.381
7.366
3.884
3.874
3.864
3.756
3.746
3.735
3.600
3.592
3.586
3.578
3.571
3.564
3.556
3.478
3.465
3.452
3.401
3.391
3.381
3.328
3.318
3.307
2.462
2.447
2.432
1.975
1.962
1.946
1.933
1.872
1.857
1.843
1.827
1.812
1.438
1.424
1.410
1.397
1.240
1.227
1.220
1.207
F
O
O
OH
N
N
N
O
Br
1.00
0.98
1.00
1.93
1.97
1.02
1.98
1.96
1.97
1.98
2.14
2.08
1.96
1.97
15
14
13
12
11
10
9
8
7
6
5
4
3
2
1
0
f1 (ppm)

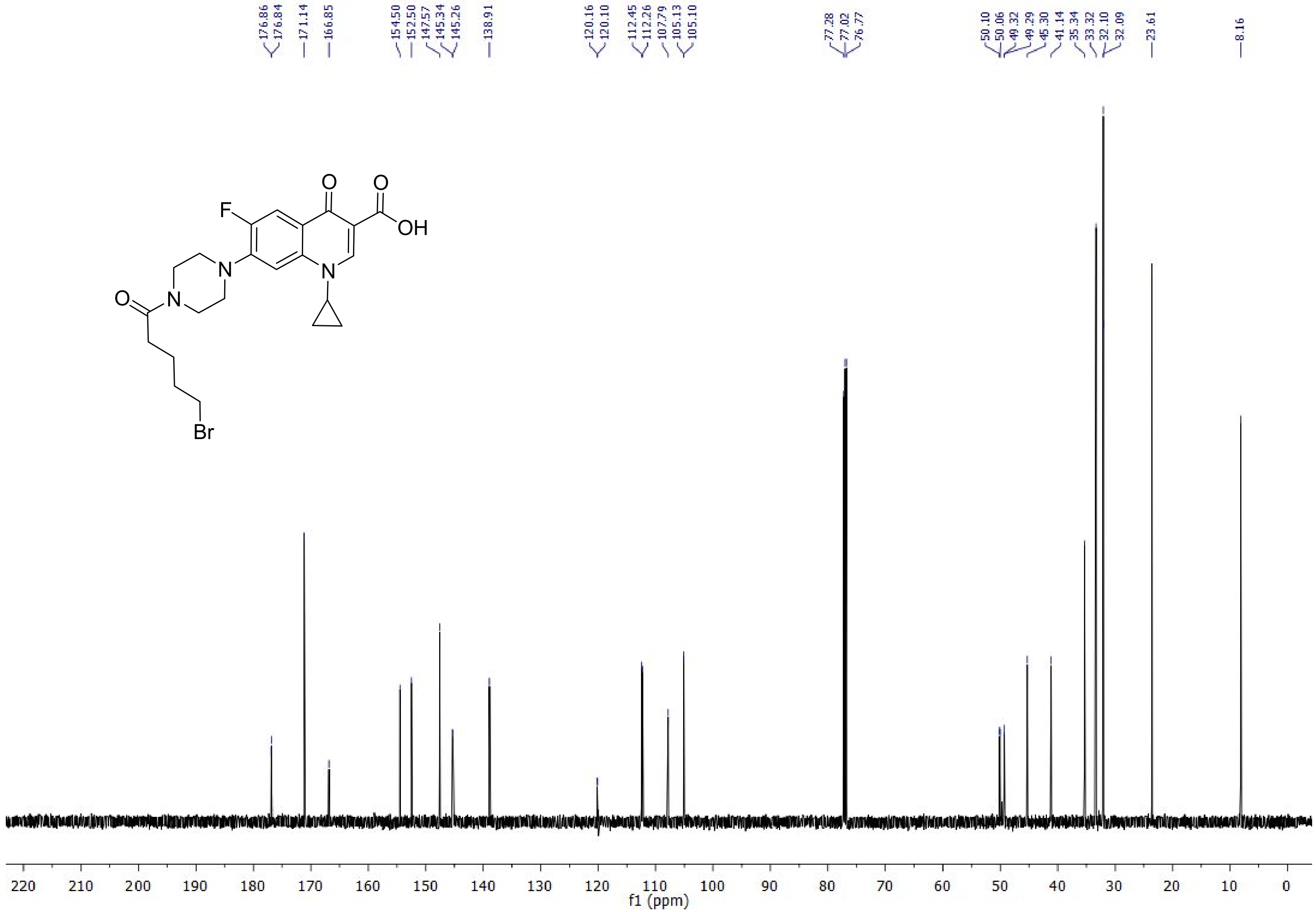
176.86
176.84
171.14
166.85
154.50
152.50
147.57
145.34
145.26
138.91
120.16
120.10
112.45
112.26
107.79
105.13
105.10
77.28
77.02
76.77
50.10
50.06
49.32
49.29
45.30
41.14
35.34
33.32
32.10
32.09
23.61
8.16
F
O
O
OH
N
N
O
N
Br
220
210
200
190
180
170
160
150
140
130
120
110
100
90
80
70
60
50
40
30
20
10
0
f1 (ppm)

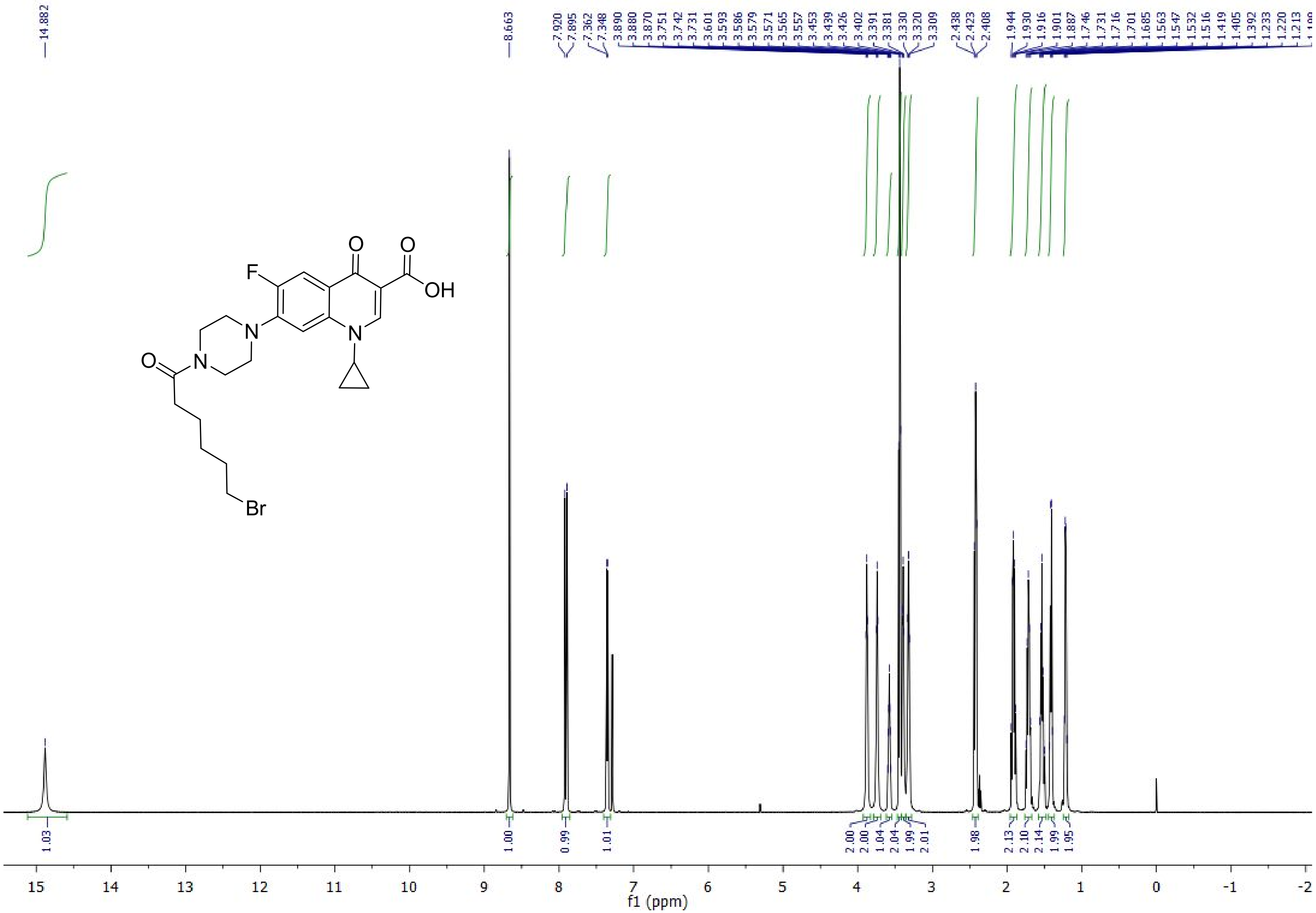

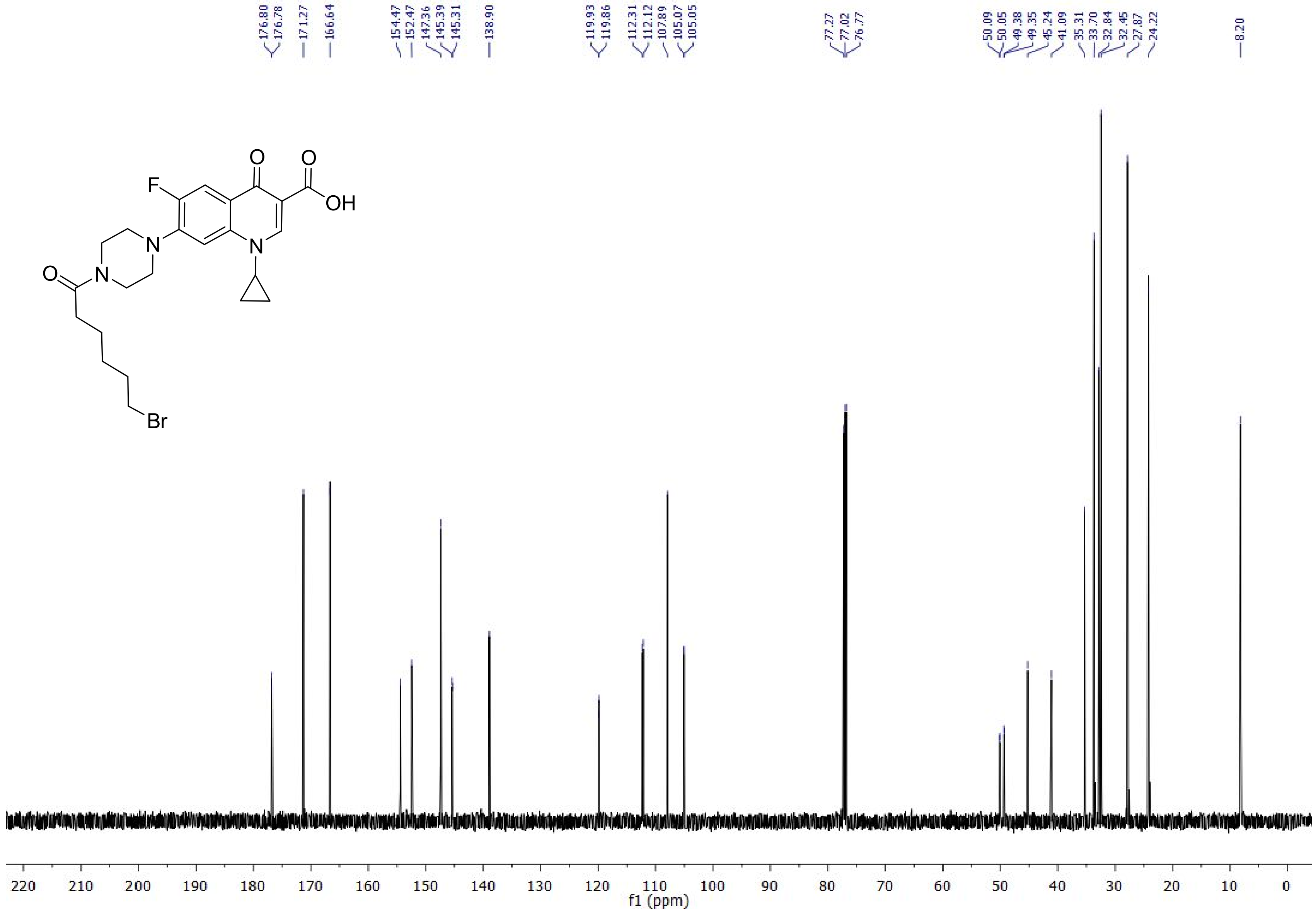
176.80
176.78
171.27
166.64
154.47
152.47
147.36
145.39
145.31
138.90
119.93
119.86
112.31
112.12
107.89
105.07
105.05
77.27
77.02
76.77
50.09
50.05
49.38
49.35
45.24
41.09
35.31
33.70
32.84
32.45
27.87
24.22
8.20
F
O
O
OH
N
N
N
O
Br
220 210 200 190 180 170 160 150 140 130 120 110 100 90 80 70 60 50 40 30 20 10 0
f1 (ppm)

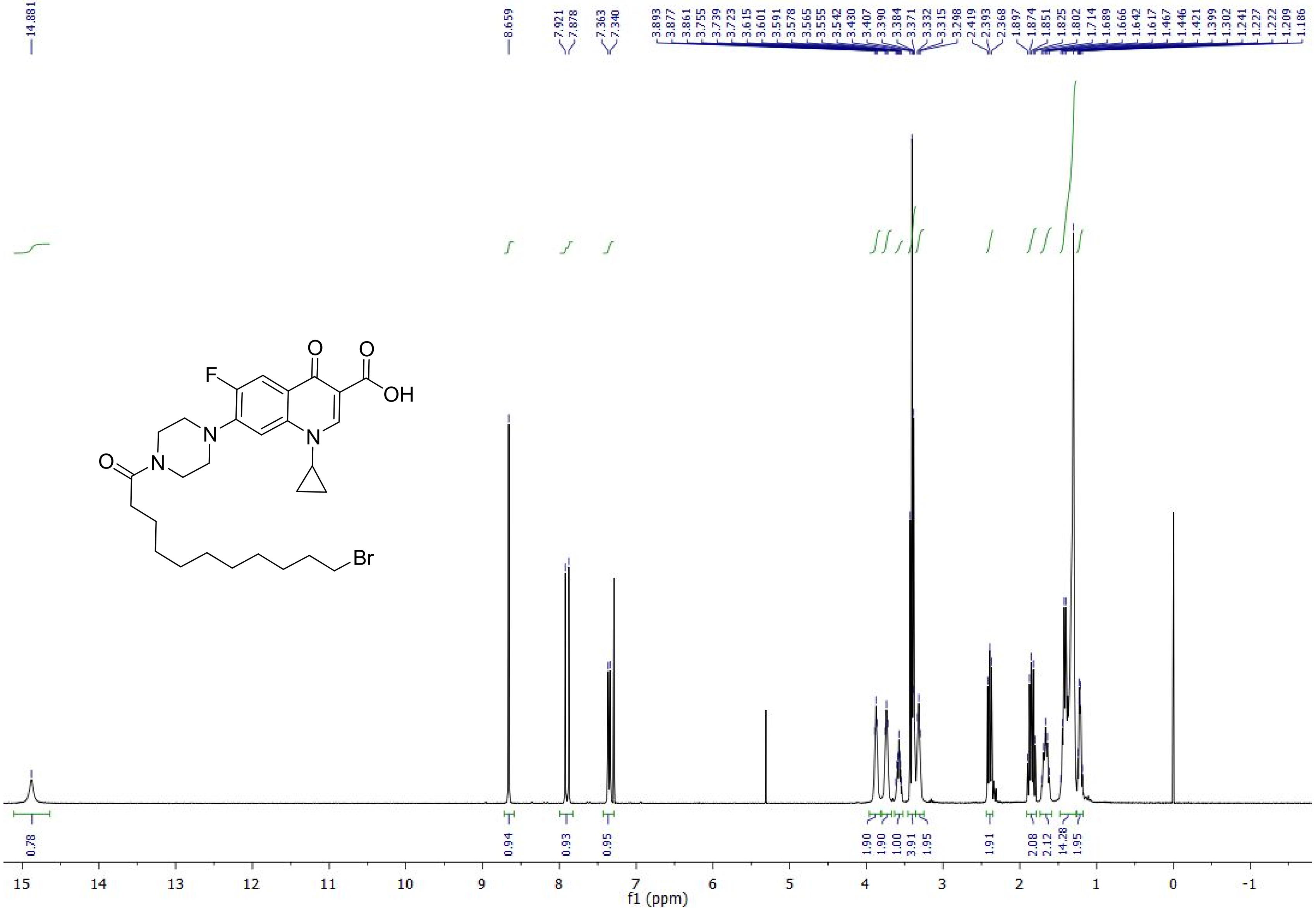

f1 (ppm)

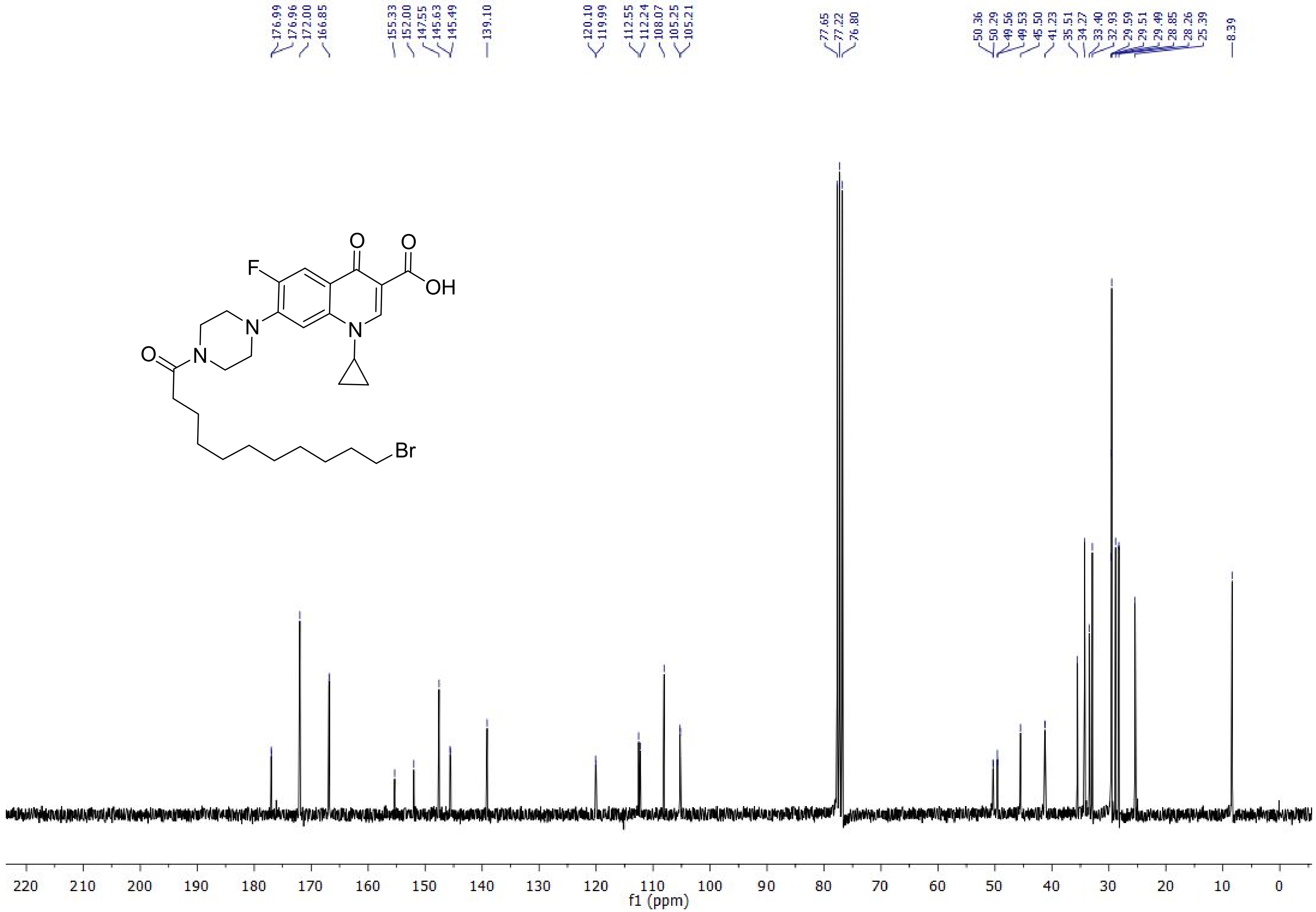
176.99
176.96
172.00
166.85
155.33
152.00
147.55
145.63
145.49
139.10
120.10
119.99
112.55
112.24
108.07
105.25
105.21
77.65
77.22
76.80
50.36
50.29
49.56
49.53
45.50
41.23
35.51
34.27
33.40
32.93
29.59
29.51
29.49
28.85
28.26
25.39
8.39
F
O
O
OH
N
N
N
O
Br
220 210 200 190 180 170 160 150 140 130 120 110 100 90 80 70 60 50 40 30 20 10 0
f1 (ppm)

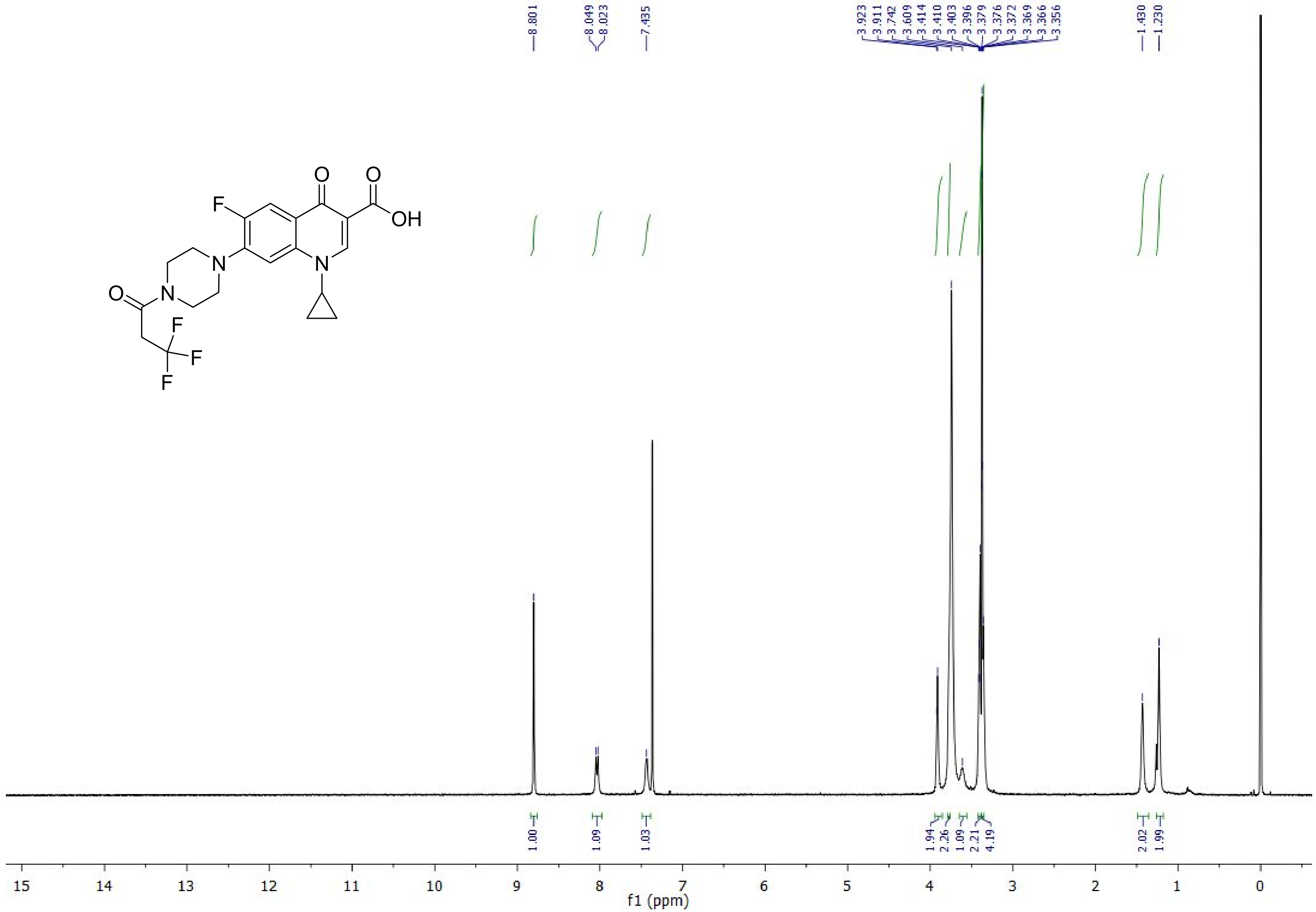

8.801
8.049
8.023
7.435
3.923
3.911
3.742
3.609
3.414
3.410
3.403
3.396
3.379
3.376
3.372
3.369
3.366
3.356
1.430
1.230
1.00
1.09
1.03
1.94
2.26
1.09
2.21
4.19
2.02
1.99
15
14
13
12
11
10
9
8
7
6
5
4
3
2
1
0
f1 (ppm)

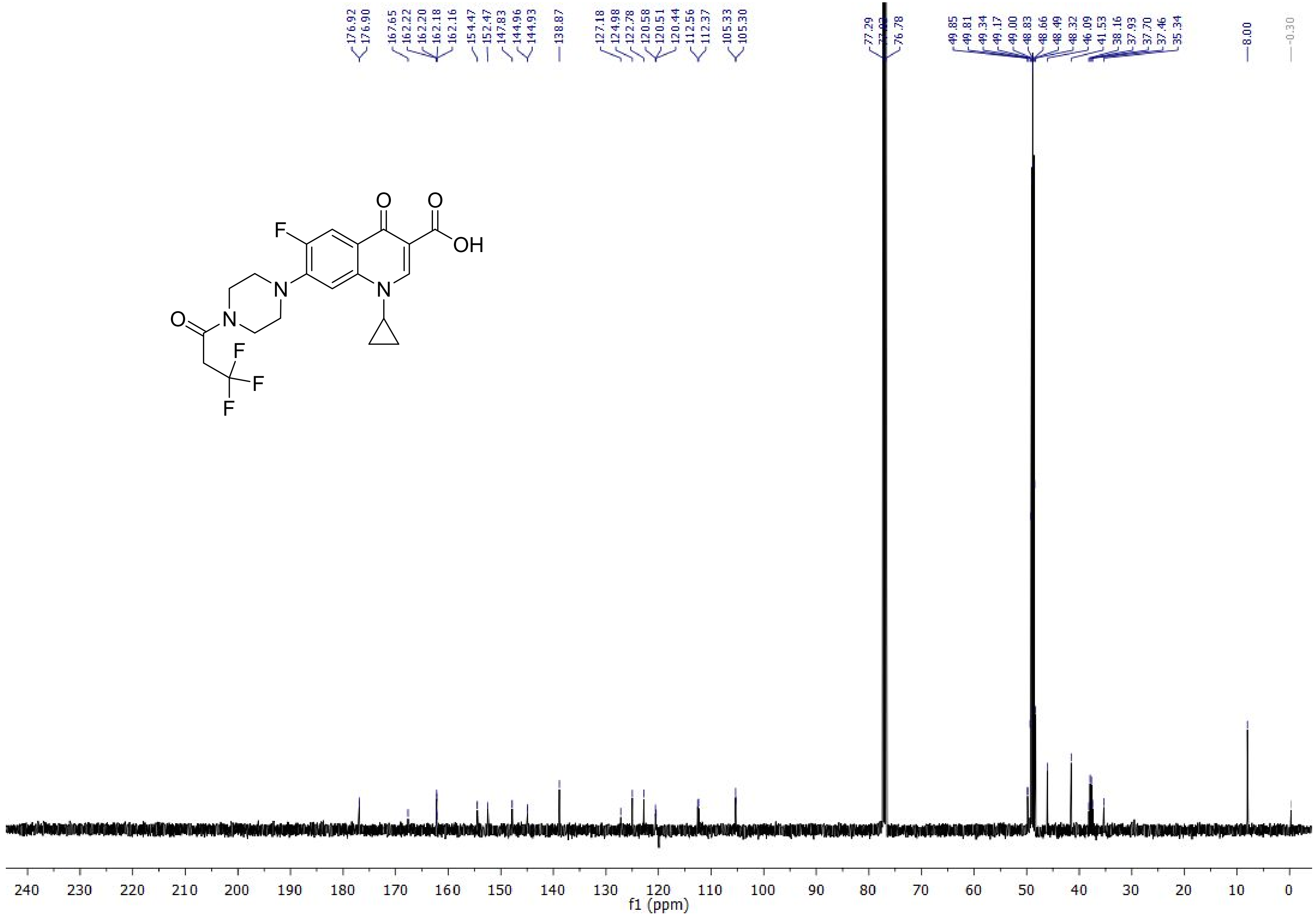

176.92
176.90
167.65
162.22
162.20
162.18
162.16
154.47
152.47
147.83
144.96
144.93
138.87
127.18
124.98
122.78
120.58
120.51
120.44
112.56
112.37
105.33
105.30
77.29
76.78
49.85
49.81
49.34
49.17
49.00
48.83
48.66
48.49
48.32
46.09
41.53
38.16
37.93
37.70
37.46
35.34
8.00
-0.30
240 230 220 210 200 190 180 170 160 150 140 130 120 110 100 90 80 70 60 50 40 30 20 10 0
f1 (ppm)
F
O
O
OH
N
N
N
O
F
F
F

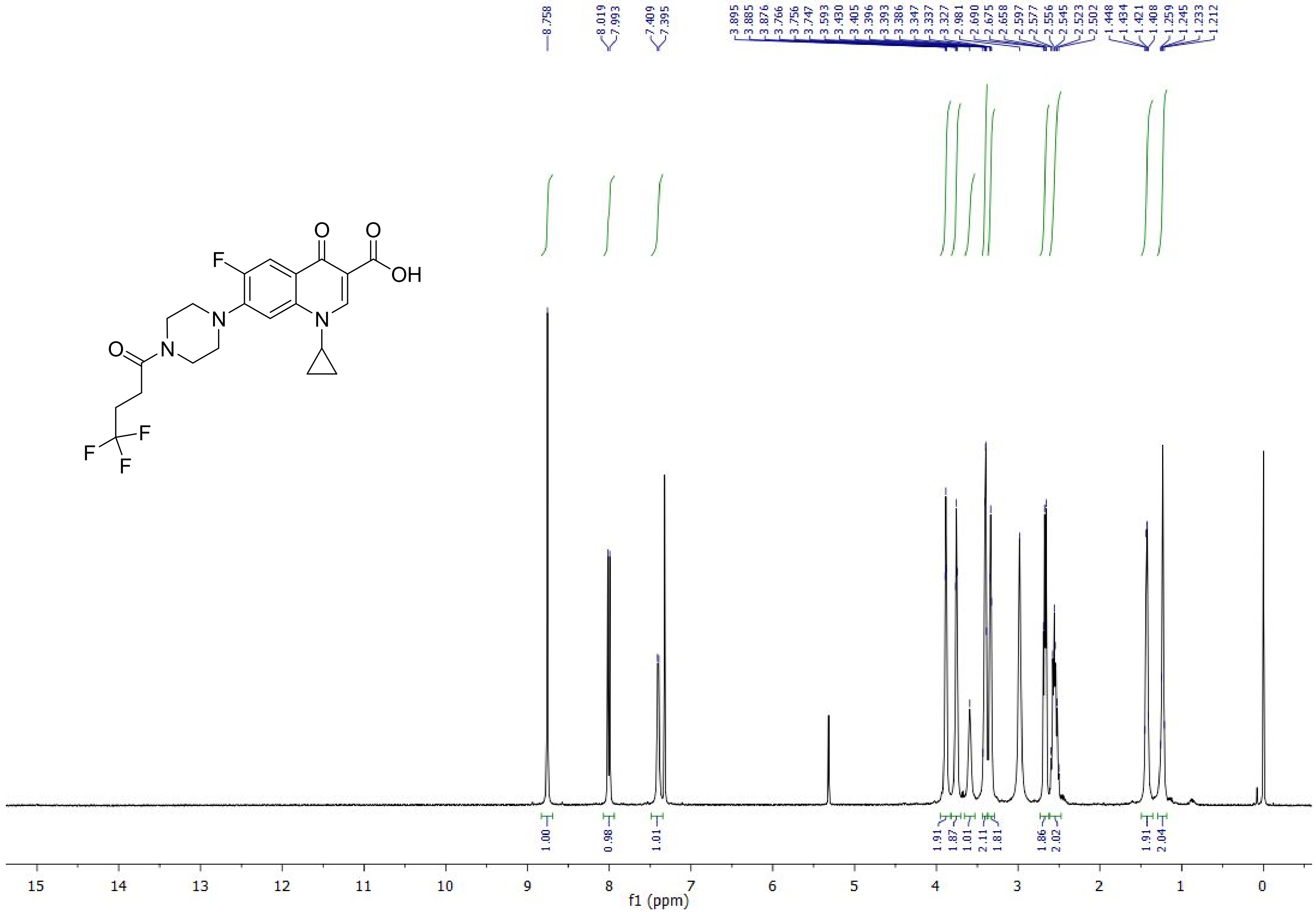
F
O
O
OH
N
N
N
O
F
F
F
8.758
8.019
7.993
7.409
7.395
3.895
3.885
3.876
3.766
3.756
3.747
3.593
3.430
3.405
3.396
3.393
3.386
3.347
3.337
3.327
2.981
2.690
2.675
2.658
2.597
2.577
2.556
2.545
2.523
2.502
1.448
1.434
1.421
1.408
1.259
1.245
1.233
1.212
1.00
0.98
1.01
1.91
1.87
1.01
2.11
1.81
1.86
2.02
1.91
2.04
15
14
13
12
11
10
9
8
7
6
5
4
3
2
1
0
f1 (ppm)

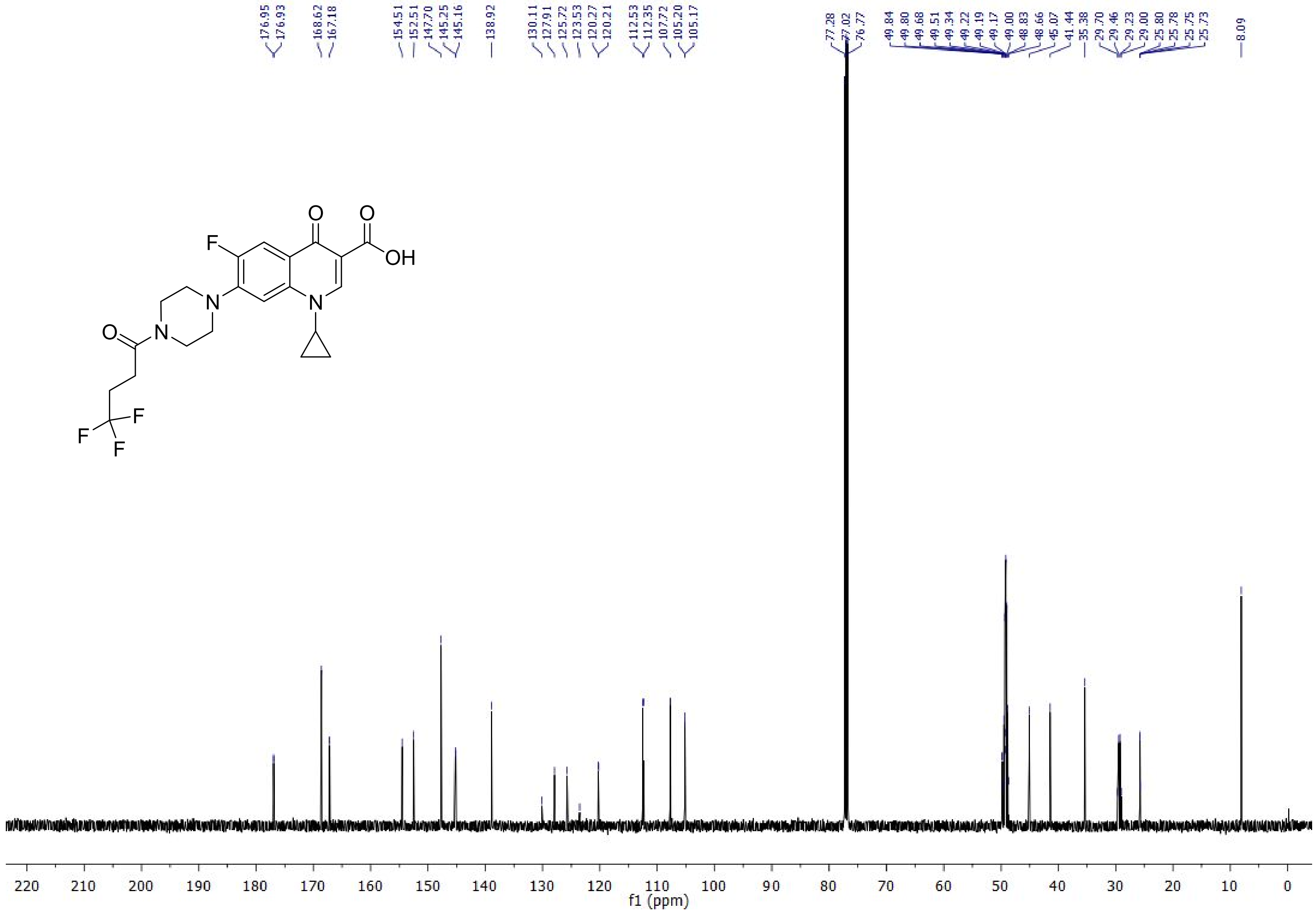
176.95
176.93
168.62
167.18
154.51
152.51
147.70
145.25
145.16
138.92
130.11
127.91
125.72
123.53
120.27
120.21
112.53
112.35
107.72
105.20
105.17
77.28
77.02
76.77
49.84
49.80
49.68
49.51
49.34
49.22
49.19
49.17
49.00
48.83
48.66
45.07
41.44
35.38
29.70
29.46
29.23
29.00
25.80
25.78
25.75
25.73
8.09
220
210
200
190
180
170
160
150
140
130
120
110
100
90
80
70
60
50
40
30
20
10
0
f1 (ppm)

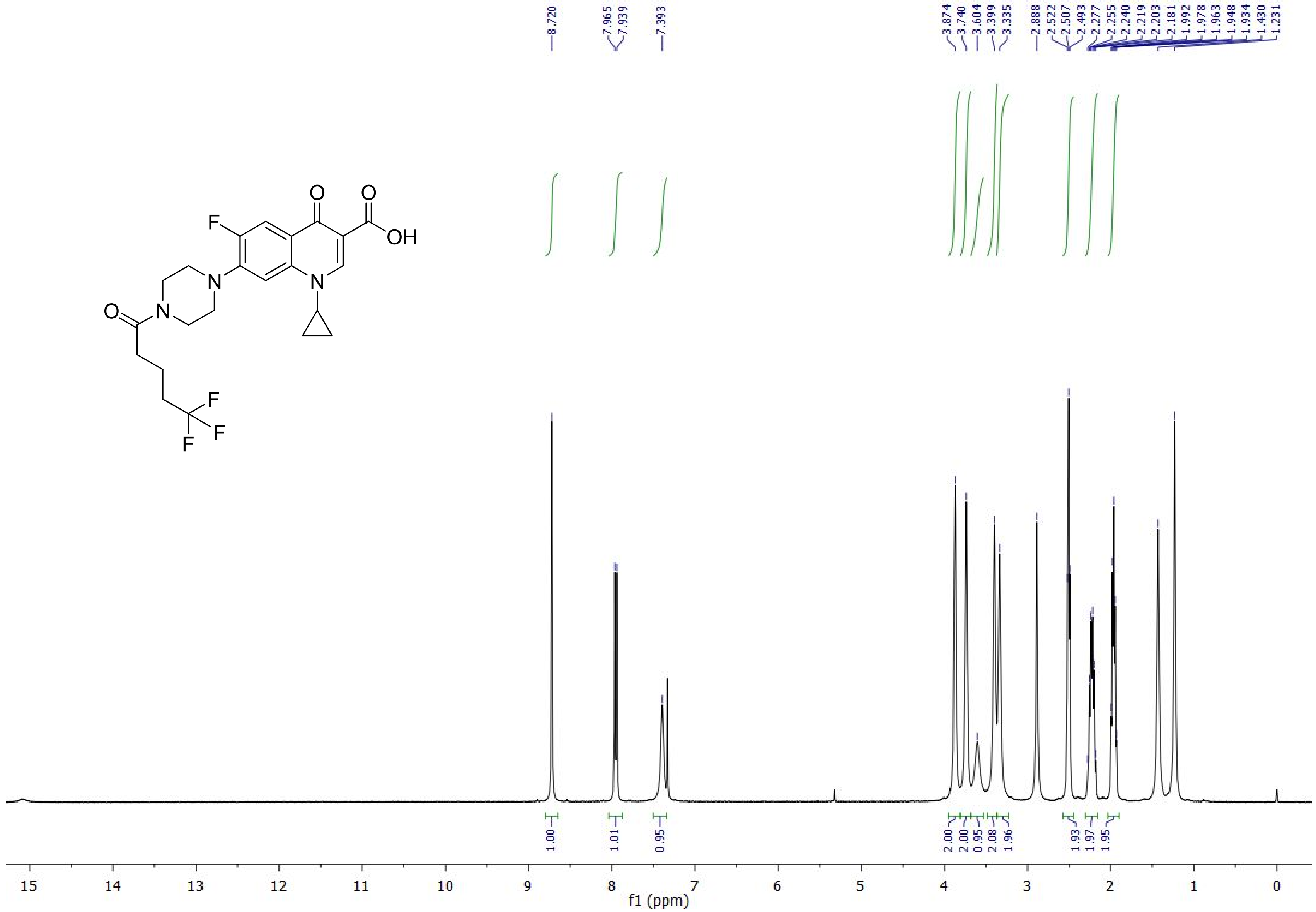

8.720
7.965
7.939
7.393
3.874
3.740
3.604
3.399
3.335
2.888
2.522
2.507
2.493
2.277
2.255
2.240
2.219
2.203
2.181
1.992
1.978
1.963
1.948
1.934
1.430
1.231
1.00
1.01
0.95
2.00
2.00
0.95
2.08
1.96
1.93
1.97
1.95
f1 (ppm)

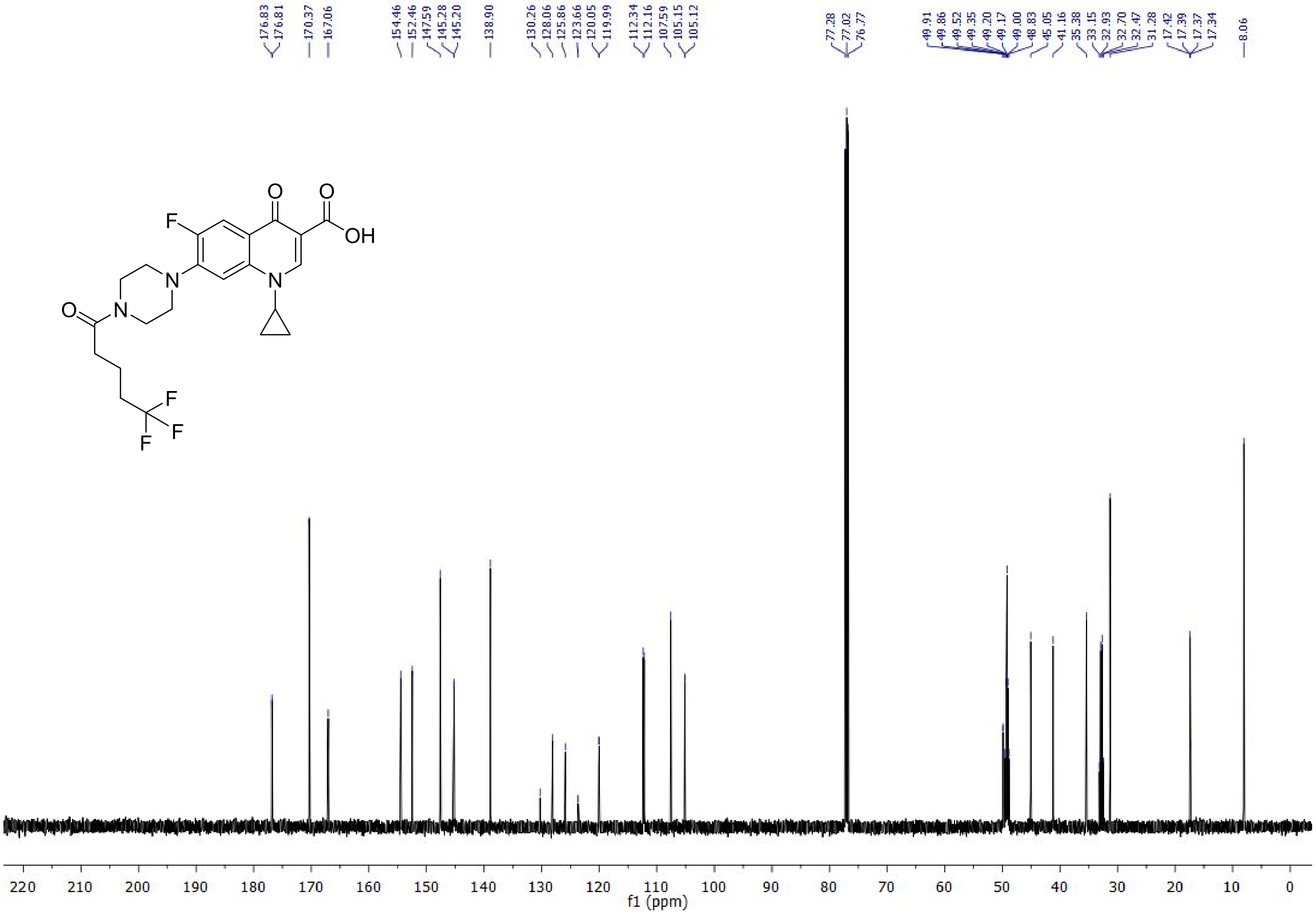
176.83
176.81
170.37
167.06
154.46
152.46
147.59
145.28
145.20
138.90
130.26
128.06
125.86
123.66
120.05
119.99
112.34
112.16
107.59
105.15
105.12
77.28
77.02
76.77
49.91
49.86
49.52
49.35
49.20
49.17
49.00
48.83
45.05
41.16
35.38
33.15
32.93
32.70
32.47
31.28
17.42
17.39
17.37
17.34
8.06
220 210 200 190 180 170 160 150 140 130 120 110 100 90 80 70 60 50 40 30 20 10 0
f1 (ppm)

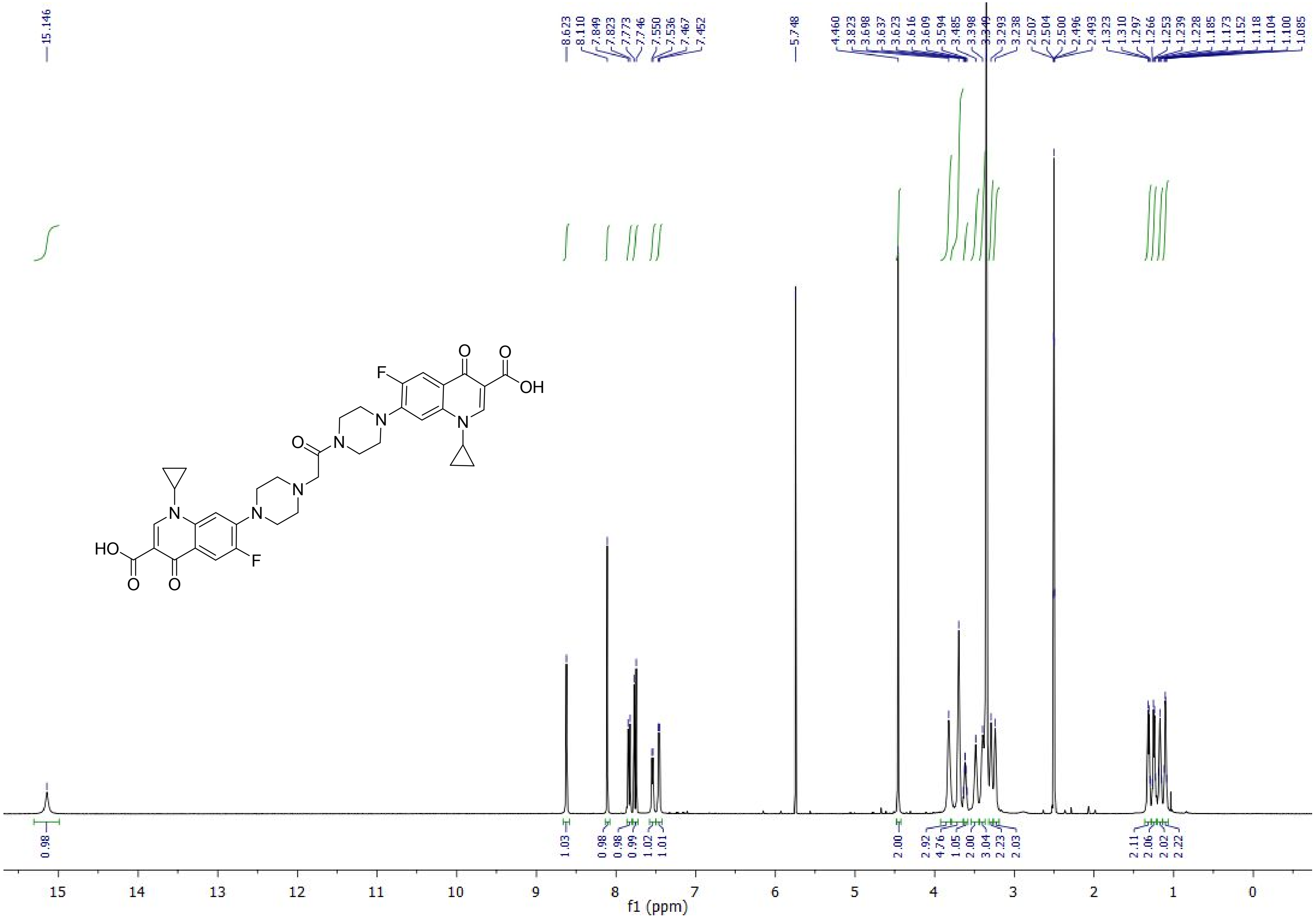

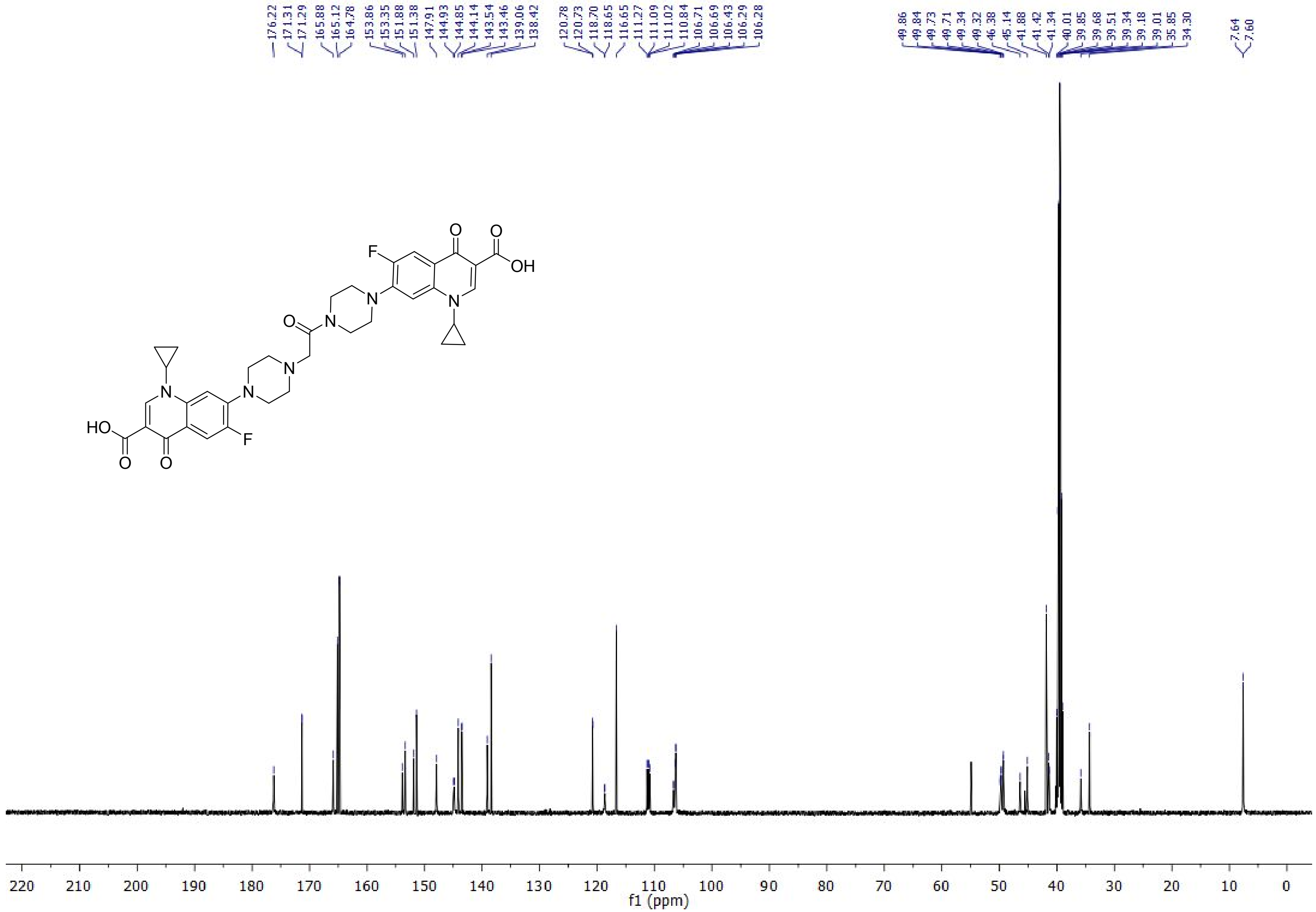
176.22
171.31
171.29
165.88
165.12
164.78
153.86
153.35
151.88
151.38
147.91
144.93
144.85
144.14
143.54
143.46
139.06
138.42
120.78
120.73
118.70
118.65
116.65
111.27
111.09
111.02
110.84
106.71
106.69
106.43
106.29
106.28
49.86
49.84
49.73
49.71
49.34
49.32
46.38
45.14
41.88
41.42
41.34
40.01
39.85
39.68
39.51
39.34
39.18
39.01
35.85
34.30
7.64
7.60
F
O
O
OH
N
N
O
N
N
N
HO
F
O
O
220 210 200 190 180 170 160 150 140 130 120 110 100 90 80 70 60 50 40 30 20 10 0
f1 (ppm)

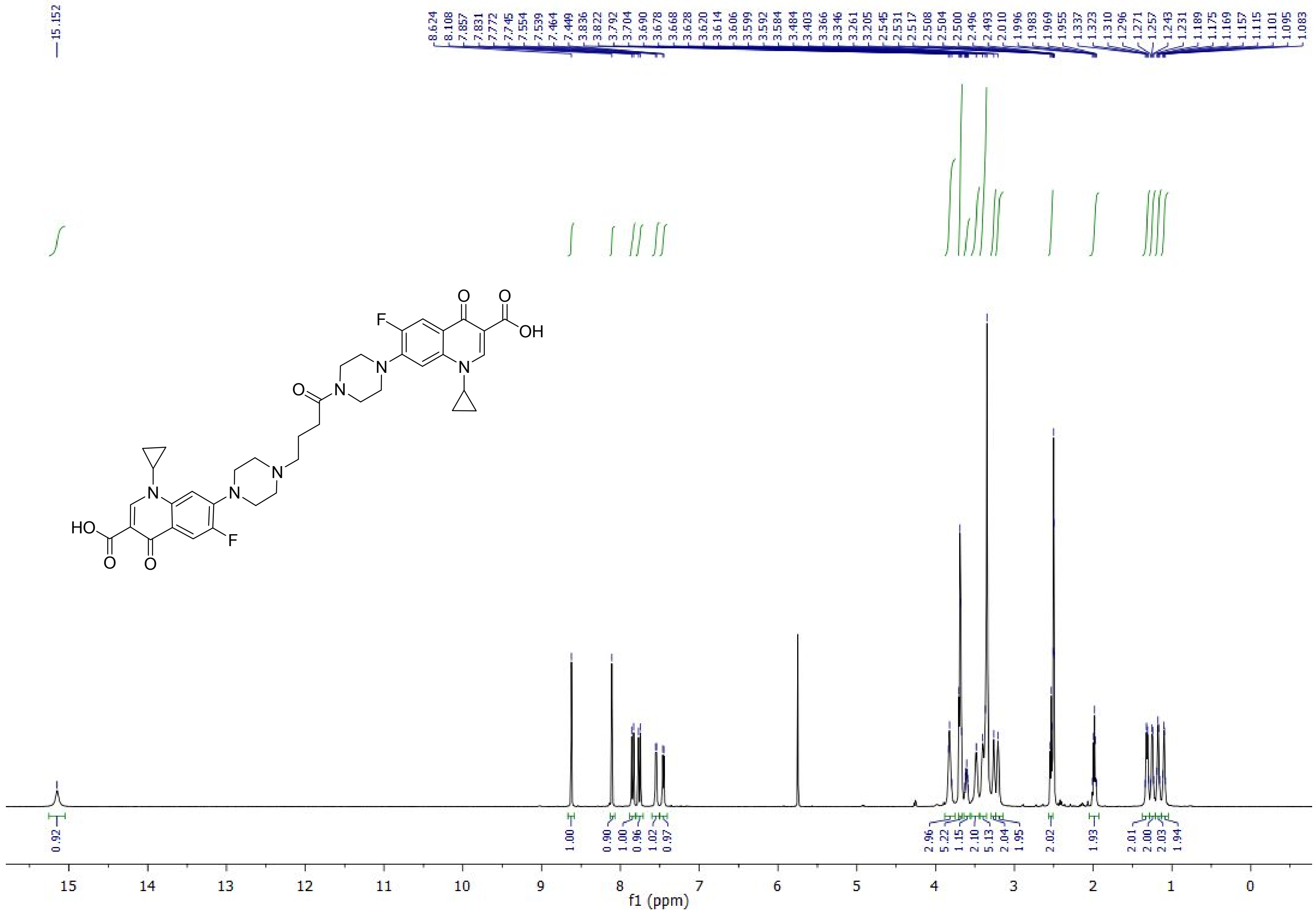

15.152
8.624
8.108
7.857
7.831
7.772
7.745
7.554
7.539
7.464
7.449
3.836
3.822
3.792
3.704
3.690
3.678
3.668
3.628
3.620
3.614
3.606
3.599
3.592
3.584
3.484
3.403
3.366
3.346
3.261
3.205
2.545
2.531
2.517
2.508
2.504
2.500
2.496
2.493
2.010
1.996
1.983
1.969
1.955
1.337
1.323
1.310
1.296
1.271
1.257
1.243
1.231
1.189
1.175
1.169
1.157
1.115
1.101
1.095
1.083
F
O
O
OH
N
N
N
O
N
N
N
HO
O
O
F
0.92
1.00
0.90
1.00
0.96
1.02
0.97
2.96
5.22
1.15
2.10
5.13
2.04
1.95
2.02
1.93
2.01
2.00
2.03
1.94
15
14
13
12
11
10
9
8
7
6
5
4
3
2
1
0
f1 (ppm)

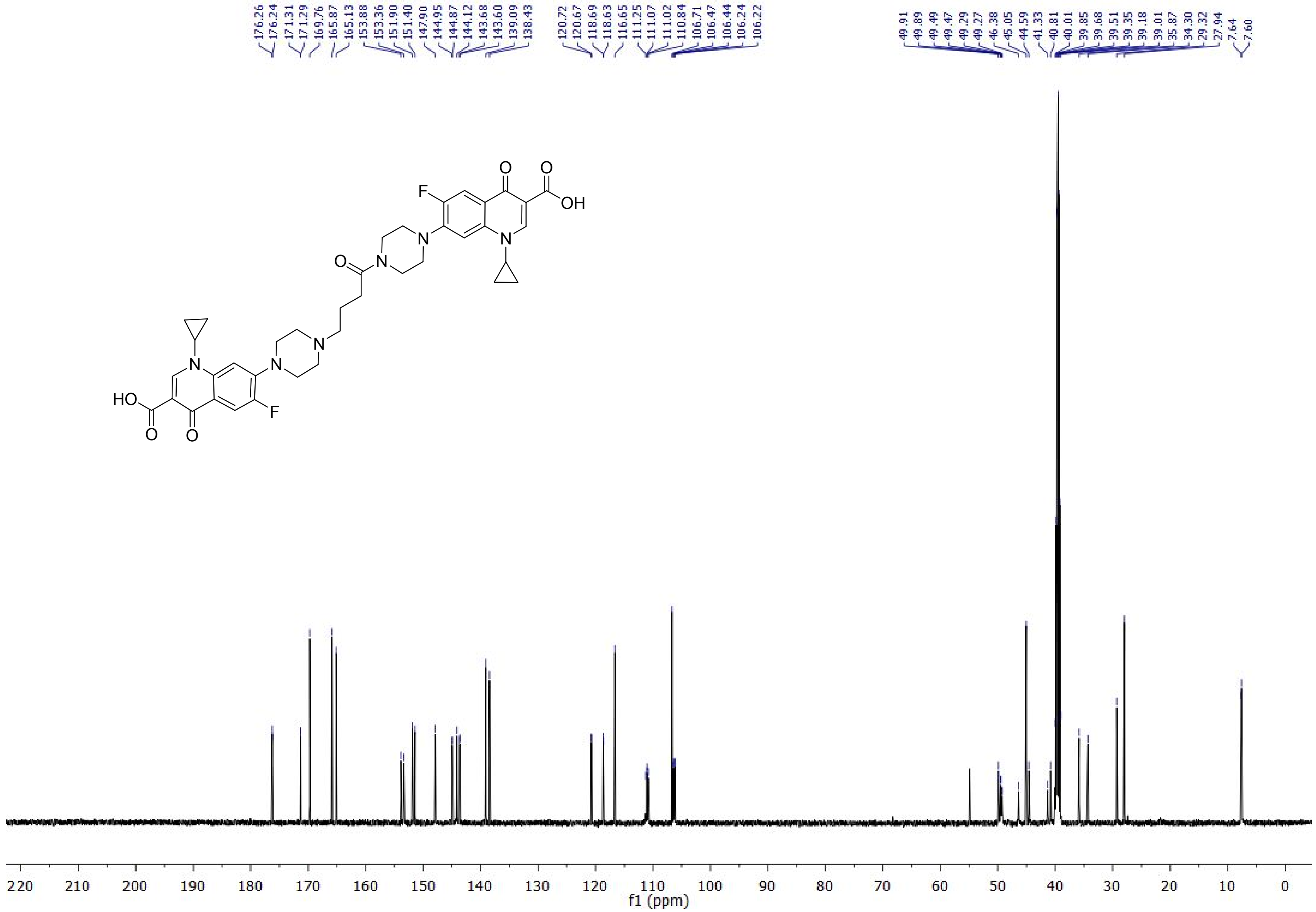
176.26
176.24
171.31
171.29
169.76
165.87
165.13
153.88
153.36
151.90
151.40
147.90
144.95
144.87
144.12
143.68
143.60
139.09
138.43
120.72
120.67
118.69
118.63
116.65
111.25
111.07
111.02
110.84
106.71
106.47
106.44
106.24
106.22
49.91
49.89
49.49
49.47
49.29
49.27
46.38
45.05
44.59
41.33
40.81
40.01
39.85
39.68
39.51
39.35
39.18
39.01
35.87
34.30
29.32
27.94
7.64
7.60
F
O
O
OH
N
N
O
N
N
N
HO
O
O
F
220 210 200 190 180 170 160 150 140 130 120 110 100 90 80 70 60 50 40 30 20 10 0
f1 (ppm)

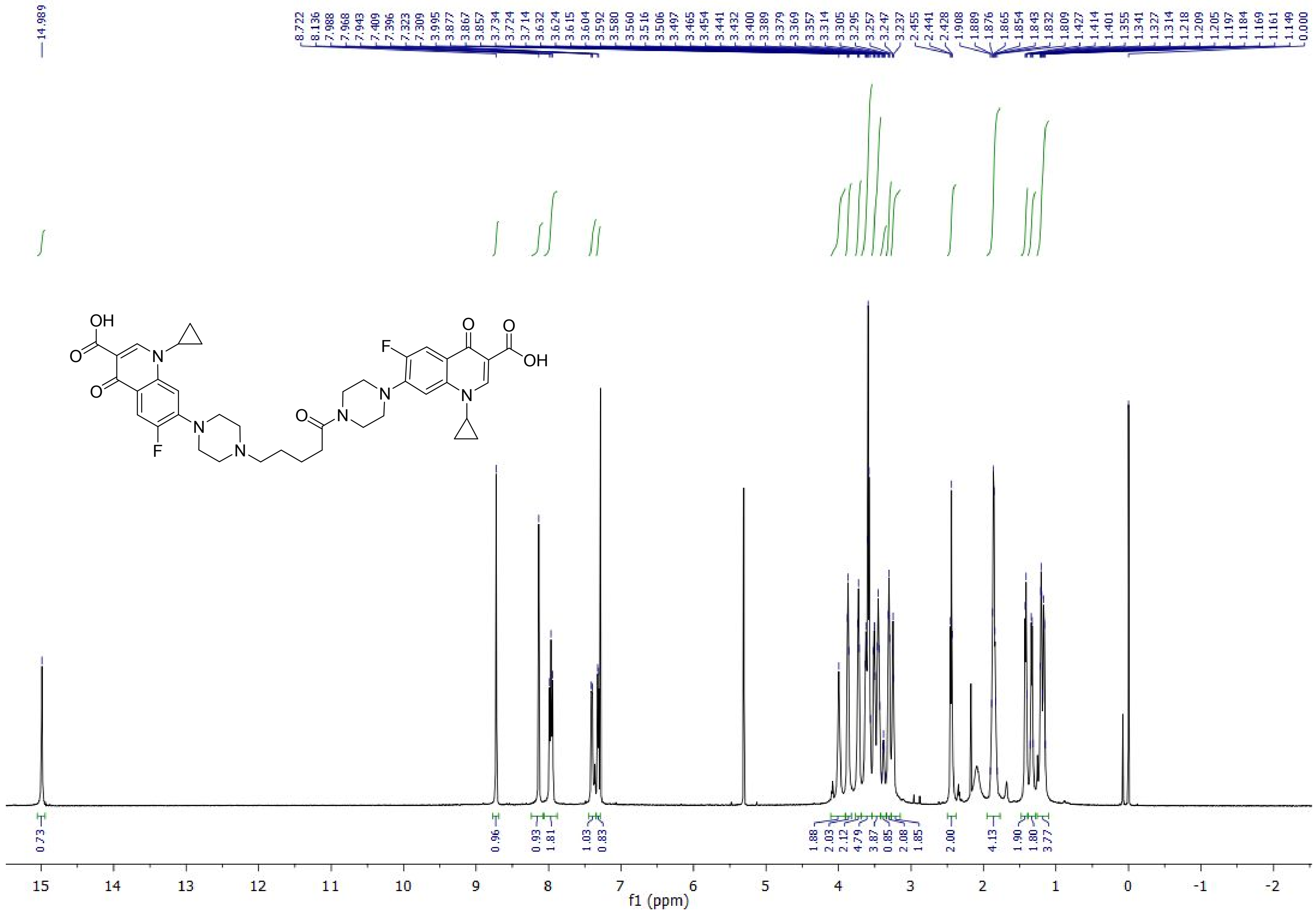

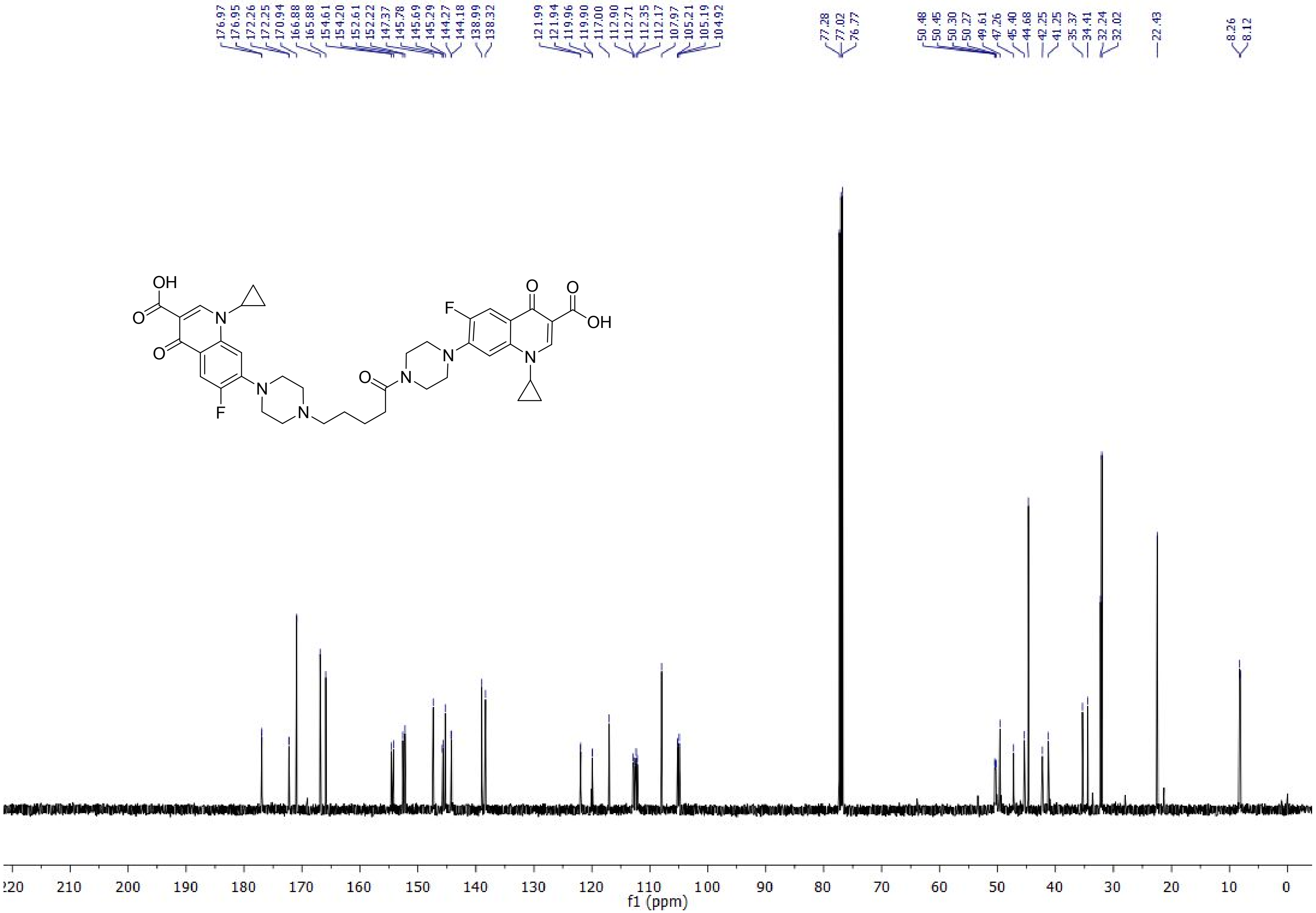
176.97
176.95
172.26
172.25
170.94
166.88
165.88
154.61
154.20
152.61
152.22
147.37
145.78
145.69
145.29
144.27
144.18
138.99
138.32
121.99
121.94
119.96
119.90
117.00
112.90
112.71
112.35
112.17
107.97
105.21
105.19
104.92
77.28
77.02
76.77
50.48
50.45
50.30
50.27
49.61
47.26
45.40
44.68
42.25
41.25
35.37
34.41
32.24
32.02
22.43
8.26
8.12
OH
O
O
N
F
N
N
O
N
N
F
O
O
OH
N
f1 (ppm)

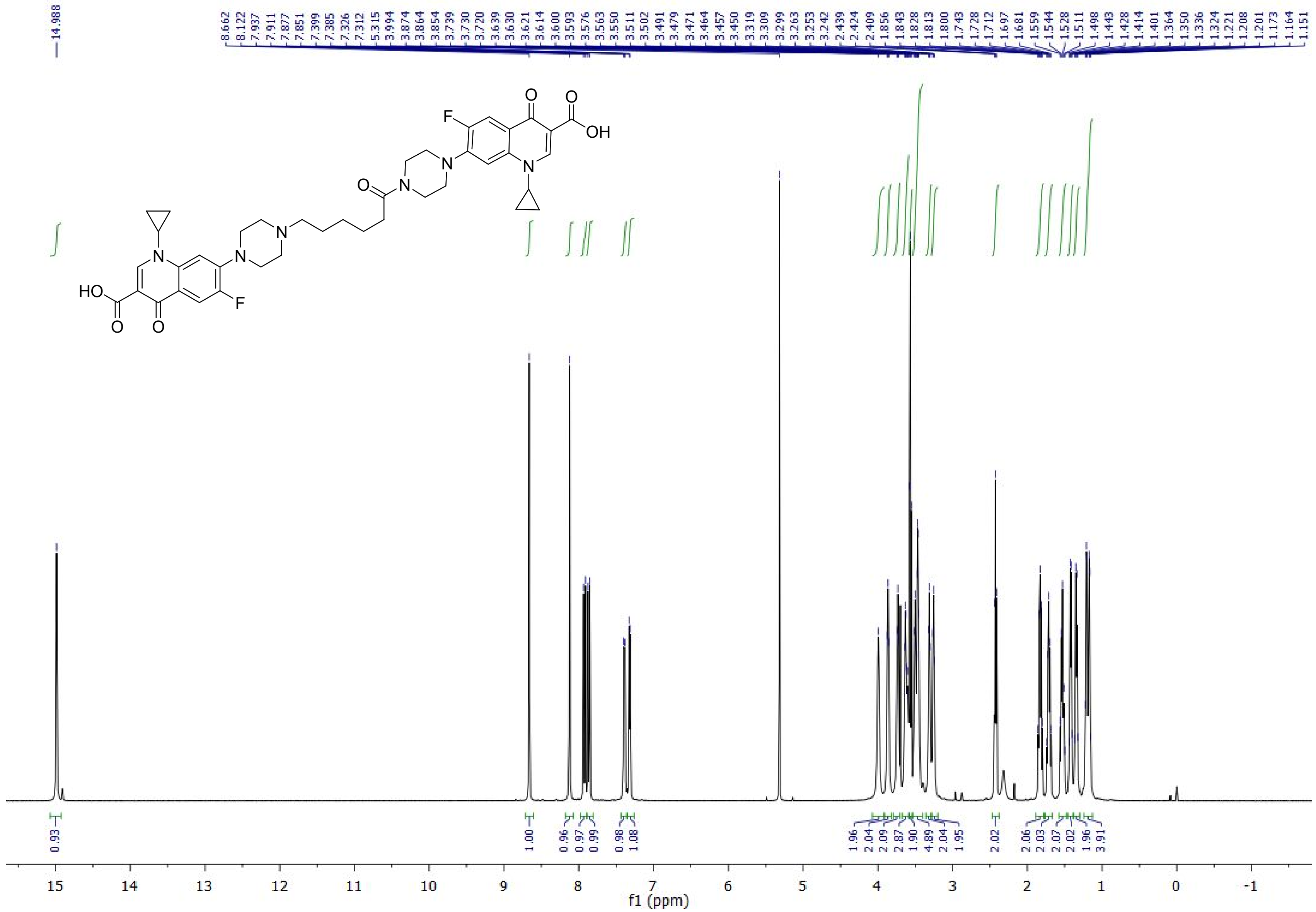

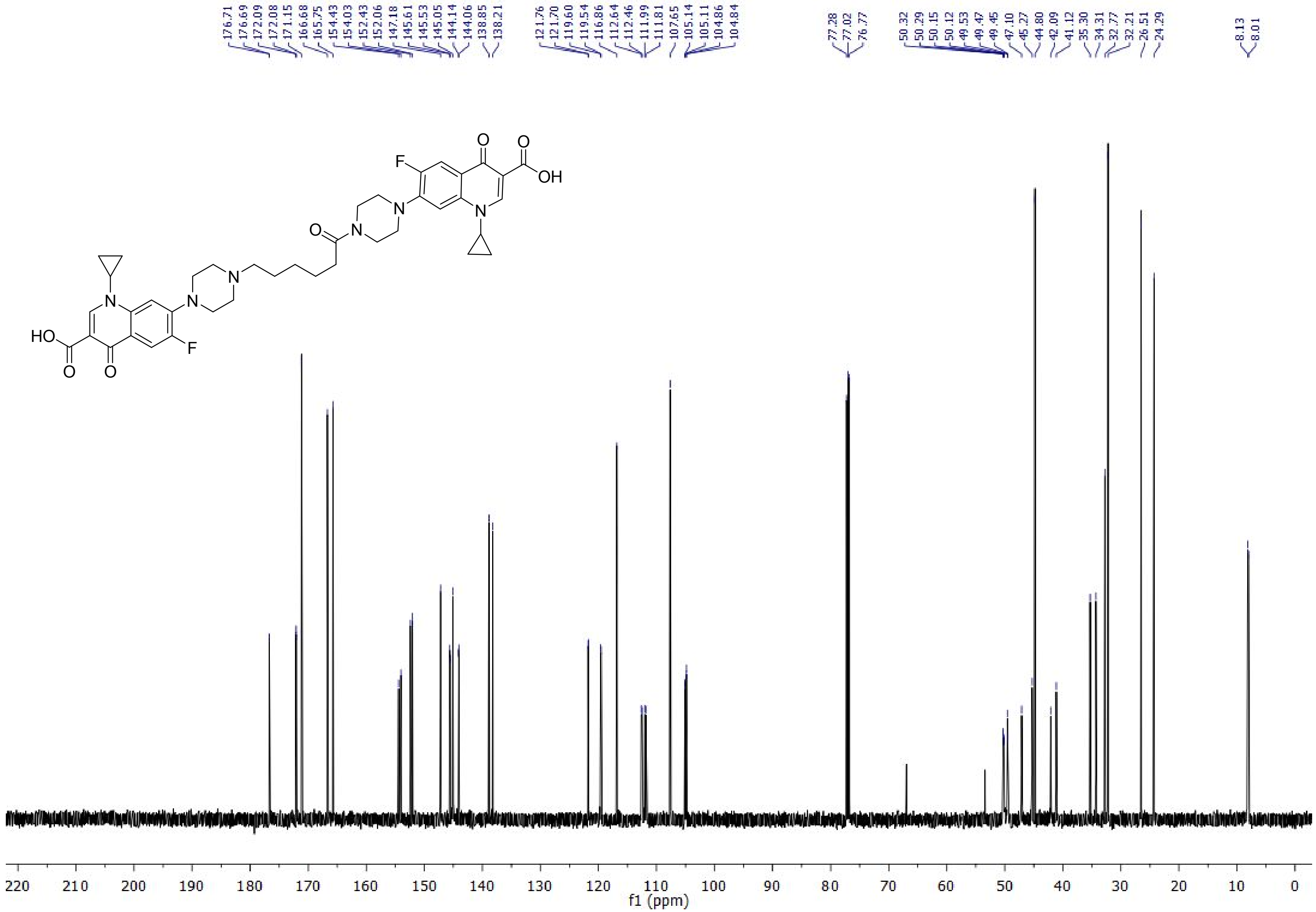
176.71
176.69
172.09
172.08
171.15
166.68
165.75
154.43
154.03
152.43
152.06
147.18
145.61
145.53
145.05
144.14
144.06
138.85
138.21
121.76
121.70
119.60
119.54
116.86
112.64
112.46
111.99
111.81
107.65
105.14
105.11
104.86
104.84
77.28
77.02
76.77
50.32
50.29
50.15
50.12
49.53
49.47
49.45
47.10
45.27
44.80
42.09
41.12
35.30
34.31
32.77
32.21
26.51
24.29
8.13
8.01
F
O
O
OH
N
N
N
O
N
HO
F
220 210 200 190 180 170 160 150 140 130 120 110 100 90 80 70 60 50 40 30 20 10 0
f1 (ppm)

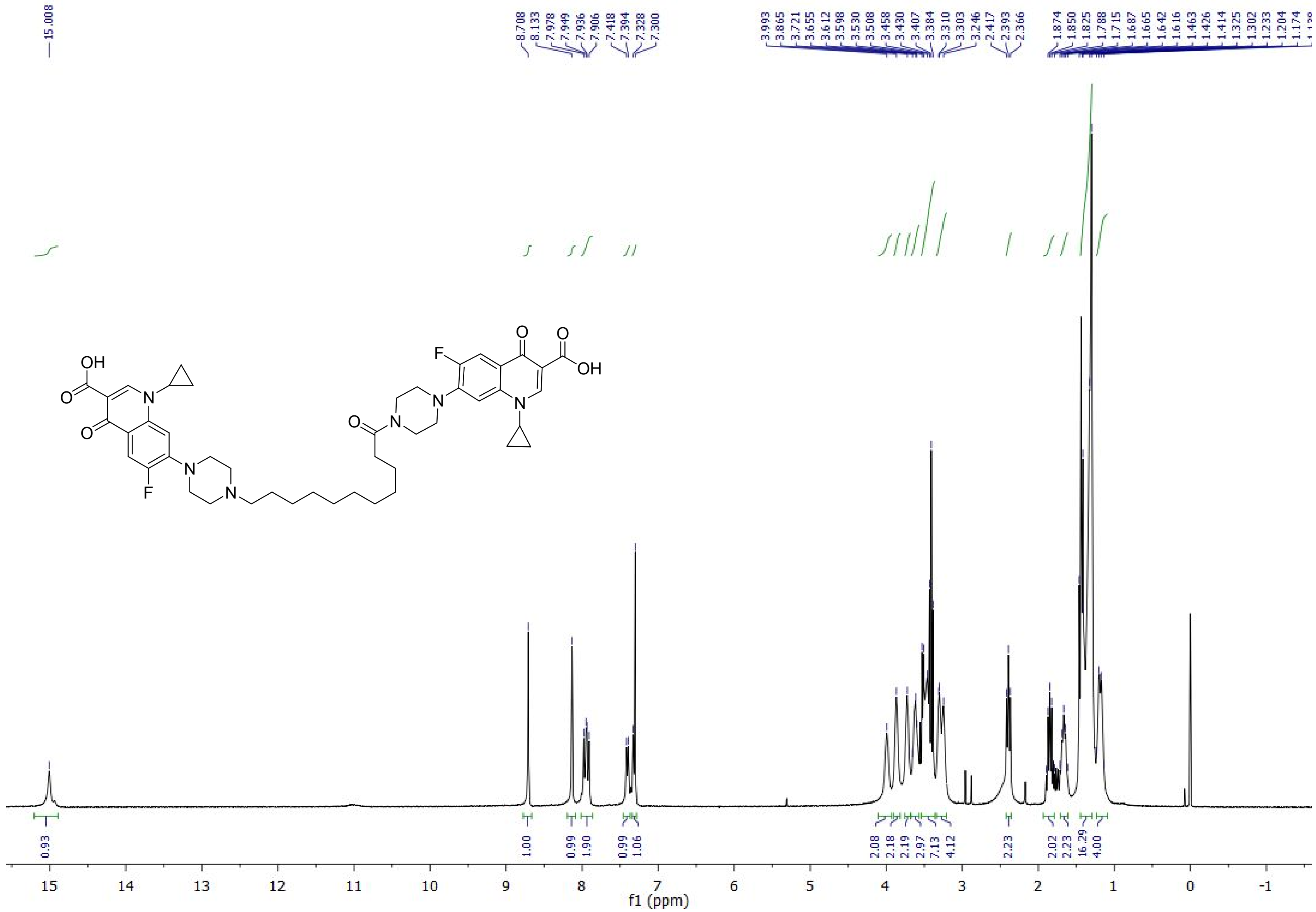

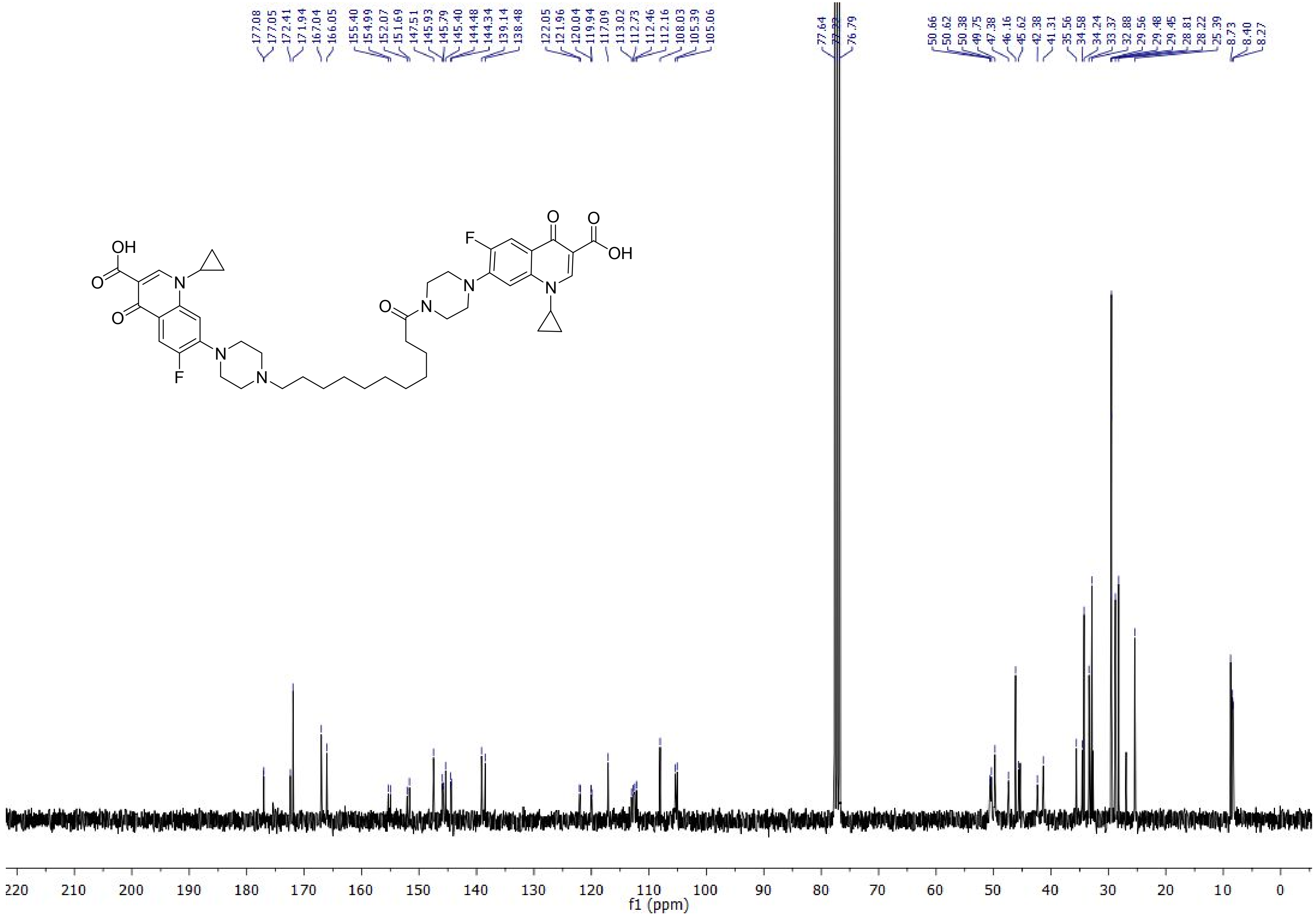

Supplement: Supplementary file 1 — ao3c00554_si_001.pdf [file ao3c00554_si_001.pdf]
